# A Reversible 900 nm Near‐Infrared Photoswitch for Invisibly Writable 2D and 3D Displays

**DOI:** 10.1002/anie.5802775

**Published:** 2026-06-10

**Authors:** Elias Ciekalski, Henry Dube

**Affiliations:** ^1^ Department of Chemistry and Pharmacy Friedrich‐Alexander‐Universität Erlangen‐Nürnberg Erlangen Germany

**Keywords:** indigoid, near‐infrared light, *peri*‐benzo[*a*]fluoranthenethioindigo, photochromic display, photoswitching

## Abstract

Molecular photoswitches interchange between different states in response to light irradiation and represent prominent tools to deliberately control changes at the smallest scales. A standing challenge for their development is pushing absorptions into the low‐energy end of the electromagnetic spectrum, which is highly desirable to avoid photodamage, allows mixing of photoresponsive components, and maximizes the available color range. Here we report on a novel photoswitch motif, *peri*‐benzo[*a*]fluoranthenethioindigo (PBFT), enabling direct excitation with near‐infrared (NIR) light up to 900 nm wavelengths in a fully reversible fashion. PBFT provides an outstanding property profile with high isomer yields, good thermal bistability, and strongly distinct color changes from pale green to deep blue. Application within a commercially available and easy‐to‐use polymer material allows to realize reversible and spatially resolved inscription of information with extremely long wavelengths of light. Writing information into versatile handheld 2D and 3D optical displays and optical memories is thus possible with non‐damaging irradiation, invisible to the naked eye.

## Graphical abstract

The *peri*‐benzo[*a*]fluoranthenethioindigo (PBFT) photoswitch enables direct excitation with near‐infrared light up to 900 nm wavelengths. It delivers a high‐performance property profile including high‐contrast color changes between bright green and deep blue. Application in commercial transparent candle wax allows generating handheld 2D and 3D optical displays that can be written with non‐damaging light, invisible to the naked eye.

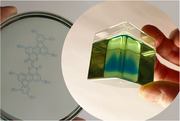

## 1 Introduction

Controlling changes of matter with single‐digit nano‐resolution is prominently enabled by molecular photoswitches, which represent indispensable precision tools applicable across the breadth of scientific disciplines. Consequentially, they are used in virtually every field from chemistry to biology, physics, or material sciences [1, 2]. Many different motifs of molecular photoswitches are available today, including classical azobenzene [3, 4], stilbene [5, 6], diarylethene [7], or spiropyranes [8, 9]. In addition, a suite of more recent types has become available, such as hydrazones [10], Stenhouse dyes [11], imidazolyl‐radicals [12, 13], homoaromatics [14], cycloaddition systems [15], or indigoids [16, 17, 18, 19]. Leaps in capability have been made in the wake of property tailoring, where especially shifting absorptions to the red and near‐infrared (NIR) end of the electromagnetic spectrum have allowed significant advances in applicability [20, 21, 22]. This is especially true for more complex photoresponses [23, 24], materials applications [25, 26, 27], as well as photopharmacology [28, 29, 30, 31, 32, 33, 34, 35], and catalysis [36, 37]. However, to achieve light responsiveness far within the red and NIR spectral region, direct excitation is very rare because red‐shifting absorptions oftentimes deteriorates other crucial properties such as thermal or photo‐stability [38, 39, 40, 41, 42]. A notable exception are recently developed diarylethene photoswitches using a specific acceptor synergistic conjugation [43, 44, 45]. In this overall context, indigoid photoswitches [16, 17, 18, 19] provide a convenient starting point as their intrinsic structures already give rise to all‐visible light responsiveness even for simpler derivatives such as indigo itself [40, 46, 47, 48], thioindigo [49], hemithioindigo [50, 51], hemiindigo [52, 53], or aurones [54]. A much‐overlooked sub‐class are represented by *peri*‐indigoids, which were first synthesized by Friedländer in the 1910s [55] and 1920s [56]. Only decades later was *peri*‐naphthothioindigo recognized as a potent photoswitch motif, but has not seen much recognition since [57, 58, 59, 60]. Recently, our group has revived this class of highly potent photochromes with *peri*‐anthracenethioindigo (**PAT**), which enabled proficient all‐red‐and NIR‐light responsive photoswitching [61, 62]. **PAT** can be photoisomerized with direct light excitation up to 830 nm, addressing the tailing absorption of its stable *E* isomer. Conversely, 590 nm orange or 625 nm red light excites the metastable *Z* isomer and leads to proficient reversed photoisomerization. A large optical window remains within the absorption covered by the two states, enabling well defined colors and facile combination with other chromophores. Only one other *peri*‐thioindigoid dye based on pyrene (*peri*‐pyrenethioindigo (**PPT**)) and developed by Mostoslavskii in 1978 possesses similar absorption and photoswitching features to our **PAT** photoswitch [63].

With such an outstanding property profile, we could use **PAT** to implement truly orthogonal and path‐independent photoswitching [64] by combining it with a second rhodanine‐based photoswitch, which was also developed in our laboratory [65]. However, direct excitation of the photoswitch was capped at 830 nm wavelength, where the absorption tails off.

**FIGURE 1:**
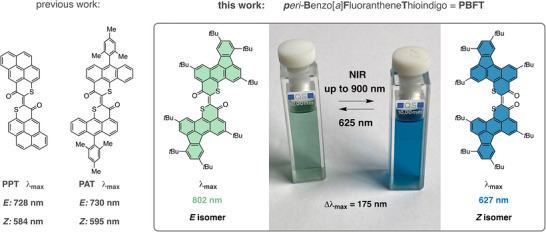
Peri‐benzo[*a*]fluoranthenethioindigo (**PBFT**) as low‐energy NIR light responsive photoswitch motif enabling direct excitation up to 900 nm wavelengths and fully reversible all‐red‐light addressability. In comparison to previous indigoid NIR photoswitches **PPT** and **PAT**, 70 nm redshifted absorption and maxima differences beyond 170 nm are achieved with **PBFT**. Note that *λ*_max_ values for **PPT** were measured in chlorobenzene solution while for **PBFT** and **PAT** the corresponding values were measured in benzene solution.

In this work, we report on yet another milestone in low‐energy molecular photoswitching with the development of the *peri*‐benzo[*a*]fluoranthenethioindigo (**PBFT**) motif (Figure 1). **PBFT** shifts the light‐addressability by another 70 nm compared to the structurally related **PAT** switch, with a maximum absorption of its stable *E* isomer at 802 nm and tailing of its absorption beyond 900 nm. The metastable *Z* isomer possesses a maximum at 627 nm, which gives rise to more than 170 nm maximum shifts between the two isomeric states. Fully reversible photoswitching with up to 900 nm NIR and 600 nm/650 nm orange/red light in very high yields gives rise to pronounced color changes and contrast between pale green and deep blue. This performance is transferrable to polymeric materials, allowing for optical memory applications like handheld 2D and 3D [66] displays using light with extremely low energy for recording. This enables “invisible” recording, where the light signal is not noticeable by the naked eye. With **PBFT**, we thus enable double bond photoisomerizations far in the NIR region, which will be of great benefit for any application of molecular photoswitches in the future.

## 2 Results and Discussion

**SCHEME 1:**
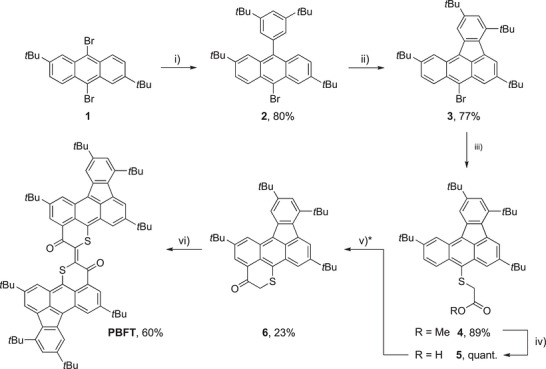
Synthesis of **PBFT**. Conditions: (i) (3,5‐di‐*te* *r* *t*‐butylphenyl)boronic acid, Pd(dba)_2_, DPEPhos, K_3_PO_4_, toluene/H_2_O, 80°C, 20 h; (ii) 2,3‐dichloro‐5,6‐dicyano‐1,4‐benzoquinone (DDQ), CH_2_Cl_2_, 0°C–23°C, 6 h; (iii) Methyl thioglycolate, Pd_2_(dba)_3_, Xantphos, *i*Pr_2_EtN, 1,4‐dioxane, 100°C, 12 h; (iv) NaOH, CH_2_Cl_2_/MeOH (2/1), 23°C, 1 h; (v) 1. PCl_5_, C_2_H_4_Cl_2_, 0°C, 10 min, 2. AlCl_3_, 0°C, 4 h, * note that the reduced single bond connected form of **PBFT** is produced already in this Friedel‐Crafts step, which is readily converted to **PBFT** in the next step; (vi) 10% NaOH_aq_, EtOH, aerated, 23°C, 1 h.

**PBFT** was synthesized in a short sequence by adapting earlier reported protocols for the generation of benzo[*a*]fluoranthenes [67] and **PAT** photoswitches [61] (Scheme 1). Starting from easily accessible dibromo‐3,7‐di‐*tert*‐butylanthracene (**1**) and commercially available (3,5‐di‐*tert*‐butylphenyl)boronic acid, a Suzuki cross‐coupling reaction yielded intermediate **2**. A Scholl reaction furnished the corresponding ring‐annulated brominated benzo[*a*]fluoranthene **3**. Subsequent cross‐coupling with methyl thioglycolate gave the corresponding thioether **4**. After hydrolysis of the ester group to yield **5**, intramolecular Friedel‐Crafts acylation led to the ring‐closed monomer **6**, which was then dimerized under basic conditions in air to give **PBFT** (see also Supporting Information for details). It should be emphasized that, different from our earlier reported synthesis of **PAT**s, in this scheme, no halogen exchange was needed for the thioether formation, saving one step in the synthesis. Interestingly, in the Friedel‐Crafts step of the synthesis, the leuco‐form of **PBFT** is formed already in a substantial amount, which is straightforwardly converted into **PBFT** under the basic aerated conditions of the last step (see Supporting Information for details).

**FIGURE 2:**
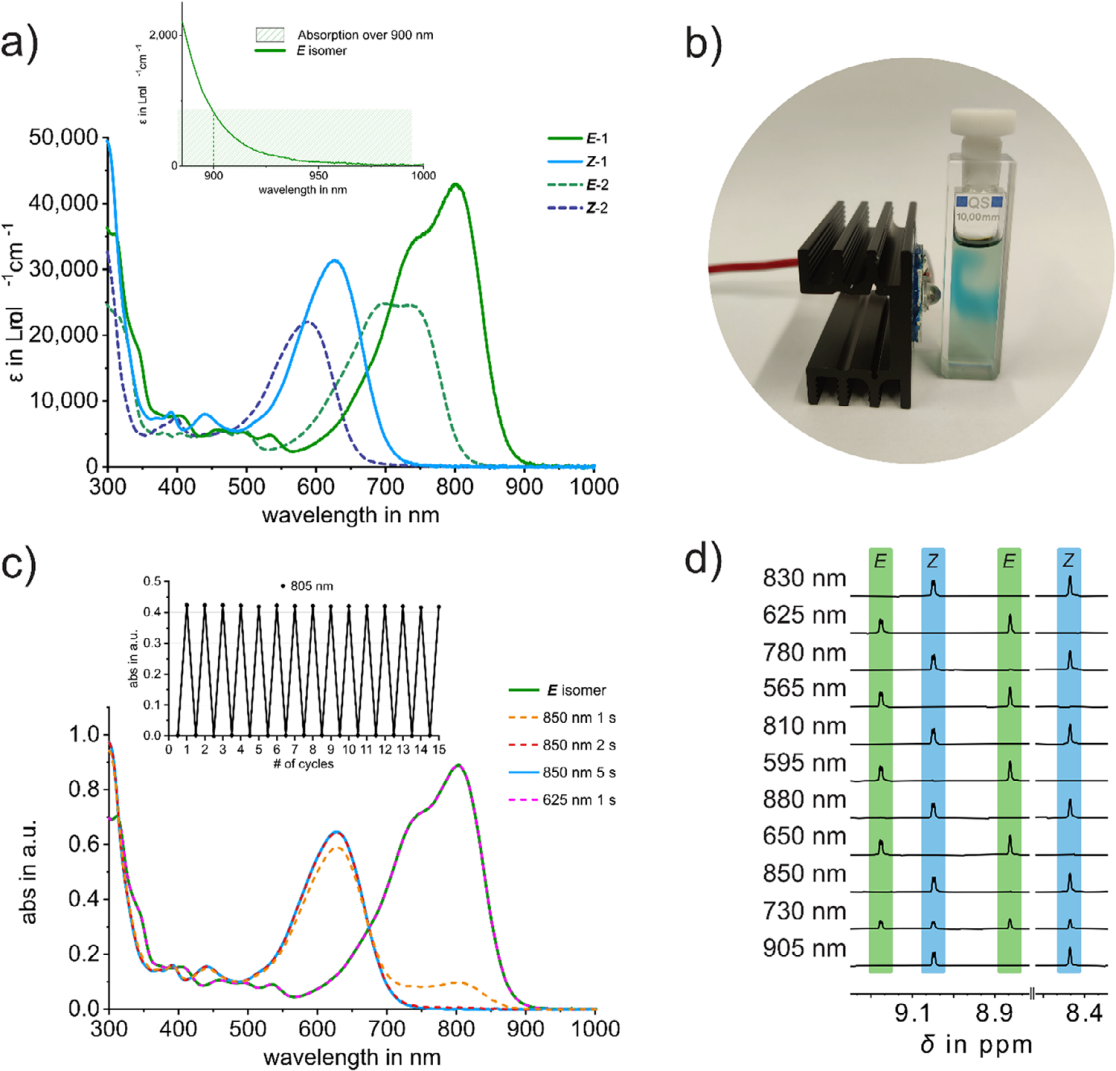
Photoswitching of **PBFT** in solution. (a) Molar absorptions of both isomers of **PBFT** in comparison to **PAT** in benzene solution at 22°C. The inset shows a zoom‐in on the absorption over 900 nm of the *E*‐**PBFT** isomer. (b) The cuvette photograph shows a snapshot of irradiating *E*‐**PBFT** in benzene solution with a 905 nm LED (switched on). Efficient local enrichment of the blue *Z* isomer is visible outpacing diffusion while the irradiating light is invisible to the naked eye. (c) Fast enrichment of both **PBFT** isomers with 850 and 625 nm LEDs. The inset shows reversible photoswitching with 850 and 625 nm light being monitored at 805 nm using UV/vis spectroscopy. (d) ^1^H NMR spectra (400 MHz, 22°C, deuterated benzene) showing efficient photoswitching of **PBFT** over a wide range of wavelengths. Irradiation wavelengths were alternated such that significant isomer changes are achieved in the photostationary state for each photoisomerization step.

After successful synthesis of **PBFT**, its (photo)physical and photochemical properties were quantified. Owing to the substantial number of *tert*‐butyl groups installed, the chromophore is very well soluble in organic solvents and can be conveniently investigated at different concentration ranges. UV/vis absorption spectroscopy in benzene solution (Figure 2a) delivered molar absorptions showing a 72 nm redshift for the longest wavelength absorption maximum of the thermodynamically stable *E* isomer of **PBFT** (802 nm, *ε* = 43 000 L mol^−1^ cm^−1^) as compared to previously reported **PAT** structures [61] (i.e., 701 nm, *ε* = 24 800 L mol^−1^ cm^−1^ and 730 nm, *ε* = 24 800 L mol^−1^ cm^−1^ at the dual maximum of mesitylene‐substituted original **PAT** photoswitch). The absorption of *E*‐**PBFT** tails out beyond 900 nm and possesses a shoulder at around 730 nm. A significantly reduced redshift of only 32 nm is observed for the metastable *Z* isomer of **PBFT** (627 nm, *ε* = 31 200 L mol^−1^ cm^−1^) as opposed to *Z*‐**PAT** (595 nm, *ε* = 22 000 L mol^−1^ cm^−1^), which overall leads to significantly more pronounced maxima separation for **PBFT** of 175 nm (Figure 2). Compared to **PAT**, the molar absorptions of **PBFT** are significantly increased, which gives rise to intense colors even in diluted solutions. Further it is noticeable, that a broad spectral window is opened between 350 and 550 nm by both isomers of **PBFT**. Importantly, this spectral window region shows lower molar absorptions relative to the absorption maxima as compared to the opened spectral window of **PAT**. Such feature is of special importance for combinations with other light‐responsive components reducing photonic cross talk. Only small solvatochromism was observed for **PBFT**, revealing a moderate polarization in the Franck‐Condon state.

Because of the very substantial absorption separation between the *E* and *Z* isomers of **PBFT**, highly proficient photoswitching is observed. Like **PAT**, **PBFT** exhibits negative photochromism, where the metastable *Z* isomer absorbs hypsochromically from the stable *E* isomer. This makes it straightforward to distinguish photothermal effects from true photochemical conversion, as the former would lead to strongly diminished metastable isomer accumulation, which we do not observe. Light between 780 nm and 905 nm is effective for quantitative *E* to *Z* photoisomerization, while light between 560 nm and 680 nm allows for the reverse *Z* to *E* photoisomerization in very high yields, that is, >90% isomer composition in the respective photostationary state. Although irradiation with a 905 nm LED only affects the tailing absorption end of **PBFT**s *E* isomer, photoisomerization proceeds smoothly as well. Despite the 850 nm or 905 nm light being invisible to the naked eye, photoisomerization can be followed easily due to the strong color contrast between the two isomers (Movie S1 and S2, Figure 2b). This directly shows the applicability of **PBFT** as an NIR light sensor. No precaution against oxygen is needed, and the photoisomerization can be repeated multiple times without visible deterioration of performance in aerated solutions (Figure 2c). Photoswitching of **PBFT** was repeated at higher concentrations using ^1^H NMR spectroscopy for quantification (Figure 2d). Again, quantitative *E* to *Z* and *Z* to *E* conversion was observed upon irradiation with NIR and red LED light under these conditions.

Thermal bistability was quantified in different solvents for **PBFT**, revealing very good performance with typical half‐lives of one and a half hours at 22°C for the metastable *Z* isomer in the dark (also see Supporting Information). Noticeable differences are observed when changing the solvent from apolar benzene to very polar pyridine, which accelerated thermal *Z* to *E* isomerization (note that pyridine can also accelerate thermal double bond isomerizations by acting as a nucleophile rather than by increasing polarity [68]). This performance makes **PBFT** very well suited for most photoswitch applications and enables full light‐control over its switching states under ambient conditions.

Comparison between **PBFT** and **PAT** [61] shows the effect of ring annulation on the photophysical and photochemical properties. Upon annulation, one additional benzene ring is connected in a fully conjugated fashion to the anthracene core structure. The effect on the absorption spectrum is quite similar to the parent benzo[*a*]fluoranthene behavior, which exhibits a ca. 50 nm redshift of its absorption as compared to unsubstituted anthracene. The further redshift by ca. 20 nm in **PBFT** is likely attributable to the +I effects of the additional *tert*‐butyl substituents. No significant conjugation effect of the mesitylene moiety in **PAT** is observed before ring annulation, which can straightforwardly be explained by the severe twisting between the mesitylene and the anthracene. This strong twist is the result of severe steric repulsion inhibiting planarization and conjugation between the two ring systems. Therefore, it can be concluded that the *peri*‐thioindigo motif is responsible for shifting the overall absorption far into the red region of the spectrum; however, most of the additional redshift in **PBFT** as compared to **PAT** is owed to the benzo[*a*]fluoranthene annulation.

To obtain quantitative data for the photoswitching efficiency of **PBFT**, we conducted quantum yield measurements for both switching directions using 720 nm light for irradiation (see Supporting Information for details). We obtained sizable quantum yields of 4%–5% for the *E* to *Z* and 42%–48% for the *Z* to *E* photoisomerization, respectively. It should be noted that these values have to be taken with a bit of caution, as thermal *Z* to *E* isomerization does happen during the measurement time. Thus, the value for *E* to *Z* photoisomerization is likely underestimated, while the *Z* to *E* and photoisomerization is likely overestimated. With these values, the quantum yields of **PBFT** are comparable to those of **PAT**.

**FIGURE 3:**
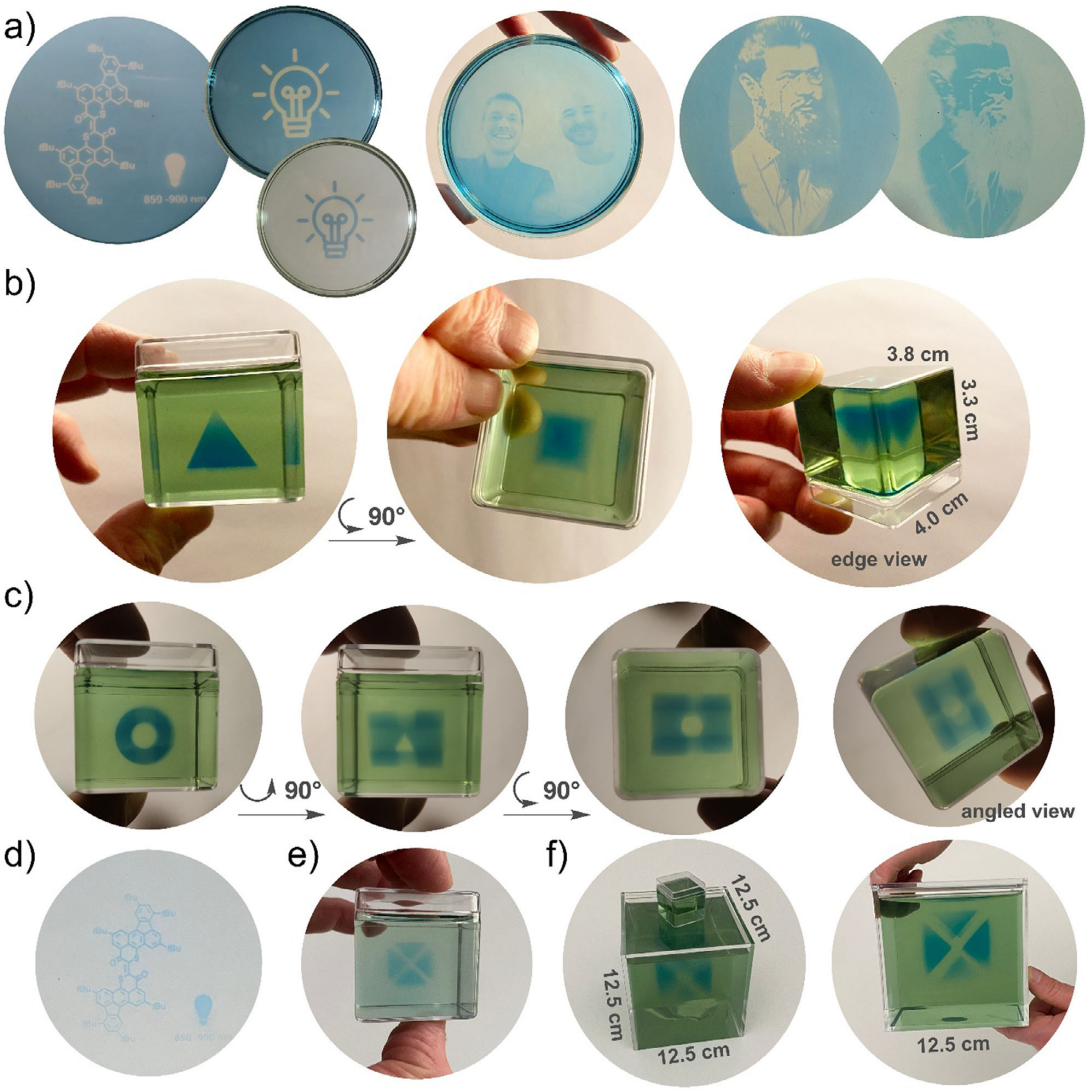
Application of **PBFT** in optical memories, 2D, and 3D displays using invisible writing with NIR light at 22°C. (a) 2D inscription of information into a commercially available transparent candle‐gel material that was mixed with **PBFT**. High contrast is achieved allowing to also inscribe photographs of the authors (middle) and Paul Friedländer (right, both as positive and negative). (b) 3D display application using the same material and simple photomasks. Inscription of a 3D pyramid shape used 850 nm light in one direction while deletion used 625 nm light at 90° to the inscription direction. (c) 3D display showing a 3D round tube with two intersecting differently shaped punctures. For writing in one direction, 850 nm light was used, while twofold deletion used 625 nm light, each at 90° to the inscription direction. (d) 2D inscription using very low concentration of the **PBFT** to obtain an almost colorless background. (e) 3D inscription using very low concentration of the **PBFT** to obtain an almost colorless background. (f) Large scale NIR writable 3D display with 12.5 cm × 12.5 cm × 12.5 cm dimensions and size comparison with the smaller cube display used in (b) and (c).

For applicability demonstrations of **PBFT** and its outstanding optical properties, we set out to create a material able to record and erase information photonically with very long wavelengths of light. In contrast to dedicatedly and specifically tailored polymeric materials that have been developed recently for 3D handheld photochromic displays [27], we sought here for an easy to process, cheap and high‐performing solid material for photoswitch embedding. This material should preferentially be commercial, available in large quantities, and low‐priced. An ideal candidate was found with transparent candle gel (Rayher Hobby 3130200 Candle Gel), which can be purchased in hundreds of g to kg scale at low cost. The candle gel is of soft jelly‐like consistency owed to being a mixture of a polystyrene‐based block polymers, mineral oil, and octadecyl 3‐(3,5‐di‐tert‐butyl‐4‐hydroxyphenyl)propionate. We expected this softness would allow the large volume changes occurring during photoswitching of **PBFT** to proceed smoothly, different to hard polymer matrices like polystyrene alone. This also required a hard plastic hosting vessel like petri dishes or plastic cubes to contain the gel. The gel liquifies at 90°C and can straight‐forwardly be mixed with a CH_2_Cl_2_ solution of **PBFT**. After cooling to 22°C, a transparent solidified green gel was obtained with tunable color intensity depending on the **PBFT** concentration. To showcase performance, low and high concentrations of **PBFT** in the candle gel were used covering two opposite ends of appearance. The materials were used for 2D optical memory applications in Petri dishes as well as for the creation of NIR‐light responsive and rewritable 3D handheld displays in commercially available plastic cubes. To this end, facile photoswitching with an 850 nm flashlight and 625 nm light enabled to inscribe and erase information reversibly into the solid materials, respectively (Figure 3, Movies S3–S10, and Supporting Information). Notably, also the thermal stability of the metastable *Z*‐**PBFT** isomer improved significantly within the candle gel, which allows storing of the inscribed information in the photochromic materials for up to 24 h in the dark (see Supporting Information). Even at high temperature of 97°C it is still possible to photoisomerize dissolved **PBFT** to its *Z* isomer and after turning off the light more than 30 s are needed to return fully to the *E* isomer Movie (S11).

The strong color contrast facilitates clear readability and long penetration depth of light into the photochromic material so that sharp 2D images and even the nuances of photographs could easily be inscribed and erased with low‐energy light irradiation at 22°C (see Figure 3a and Movie S3). To showcase the high performance of reversible information inscription, the structural formula of *E*‐**PBFT** as well as positive and negative images of a bulb were recorded in the handheld Petri dish (Figure 3a). Further, photographs of the authors and a positive and negative photograph of Paul Friedländer were recorded onto the same plate (Figure 3a). An animated 2D movie showing the rotation of **PBFT** itself during transformation from the *E* to the *Z* isomer as structural formula was created from individual frames obtained by repeated inscriptions into the same material (Movie S3).

Recording of 3D information into handheld polymeric cubes was conducted using a simple printed‐out photomask approach and the same candle gel material at 22°C. Writing of a blue 3D object across the length of the cube (4.0 cm × 3.3 cm × 3.8 cm) was done using a high‐power 850 nm flashlight (see Supporting Information and Movie 4 for further details). Deletion and shape refining in the other 2 dimensions was accomplished using a black cutout photomask at 90° to the initial inscription under focused 625 nm LED irradiation (see Supporting Information for details). Full deletion of the inscription was also done with a 625 nm LED. In this way, different 3D objects with defined shapes within the same handheld transparent cube could be inscribed (Figure 3b,c,e, Movies S5–S9). To elaborate the 3D object, a tube with two differently shaped intersecting openings was created (Figure 3c, Movie S8). To this end the tube was inscribed with 850 nm light first. Angled at 90° the tube was “cut” and a triangular opening was introduced using 625 nm light. Angled again at 90° a second round opening was inscribed in the third dimension showcasing refined shape control over the inscribed object.

Further, we tested lower concentrations of **PBFT** in order to obtain almost colorless background in the 2D display (Figure 3d). Because of the high color contrast, inscribed information is still easily visible in this case. Likewise, a much brighter background is achieved in the corresponding 3D displays with well discernible objects inscribed (Figure 3e, Movie S10).

Lastly, we increased the size of the cube significantly to dimensions of 12.5 cm × 12.5 cm × 12.5 cm (Figure 3f, Movie S12). At this large size viable inscription of 3D objects is still easily possible using two conjoint flashlights within 5 min of irradiation time and printed photomasks. Erasing at 90° to the inscription was easily done using a projector displaying plane yellow color and cardboard shadow masks (see Supporting Information for details). With this large 3D displays in hand, we could also record a 3D movie (Movies S13 (red/cyan view) and S14 (side by side view)) showing the dimensionality of the object directly. To test the limits for NIR light penetration through the material, two such large 3D cubes were stacked on top of each other and the 850 nm flashlight was placed directly on top. Within 1 min full penetration of the light to the bottom was observed (Movie S15, Figure S40) showing full viability of the photoswitching along 25 cm path length. Long‐term stability tests showed that the materials remain fully functional over the course of more than 6 months.

In summary, we report on the **PBFT** structural motif, which can directly be excited with > 900 nm light to undergo facile photoswitching. Very high isomer accumulation is achieved in both switching states using NIR and red‐light irradiation. The combination of its very advantageous properties, that is, facile and quantitative photoswitching, responsiveness to light invisible to the naked eye, strong absorption, good thermal and photostability, and high color contrast between green and blue makes **PBFT** an outstanding candidate for low‐energy light‐responsive applications. Potent applicability is demonstrated by the realization of NIR active optical memory devices for reversible inscription, NIR light detection, and erasure of information as well as 2D and 3D hand‐held displays that also can be operated with red and NIR light. We are expecting **PBFT** to become a versatile and impactful photoswitch motif that will be of great interest for anyone using light‐responsive molecular tools.

## Author Contributions

**Elias Ciekalski**: conceptualization, methodology, investigation, formal analysis, visualization, writing – original draft, writing – review and editing, data curation, validation. **Henry Dube**: conceptualization, investigation, validation, supervision, funding acquisition, formal analysis, visualization, project administration, resources, writing – original draft, writing – review and editing, methodology.

## Conflicts of Interest

The authors declare no conflicts of interest.

## Supporting information




**Supporting File 1**: anie72953‐sup‐0001‐MovieS1‐S15.zip.

**Supporting File 2**: anie72953‐sup‐0002‐SuppMat.pdf.

## Data Availability

The data that support the findings of this study are available in the Supporting Information of this article or directly from the authors upon reasonable request.

## References

[1] Z. L. Pianowski , Molecular Photoswitches. Chemistry, Properties, and Applications (Wiley‐VCH, 2022), 10.1002/9783527827626.

[2] B. L. Feringa and W. R. Browne , Molecular Switches (Wiley‐VCH, 2011), 10.1002/9783527634408.

[3] H. M. Bandara and S. C. Burdette , “Photoisomerization in Different Classes of Azobenzene,” Chemical Society Reviews 41 (2012): 1809–1825, 10.1039/c1cs15179g. 22008710

[4] S. Crespi , N. A. Simeth , and B. König , “Heteroaryl Azo Dyes as Molecular Photoswitches,” Nature Reviews Chemistry 3 (2019): 133–146, 10.1038/s41570-019-0074-6.

[5] D. H. Waldeck , “Photoisomerization Dynamics of stilbenes,” Chemical Reviews 91 (1991): 415–436, 10.1021/cr00003a007.

[6] D. Villarón and S. J. Wezenberg , “Stiff‐Stilbene Photoswitches: From Fundamental Studies to Emergent Applications,” Angewandte Chemie International Edition 59 (2020): 13192–13202, 10.1002/anie.202001031. 32222016 PMC7496324

[7] M. Irie , T. Fukaminato , K. Matsuda , and S. Kobatake , “Photochromism of Diarylethene Molecules and Crystals: Memories, Switches, and Actuators,” Chemical Reviews 114 (2014): 12174–12277, 10.1021/cr500249p. 25514509

[8] L. Kortekaas and W. R. Browne , “The Evolution of Spiropyran: Fundamentals and Progress of an Extraordinarily Versatile Photochrome,” Chemical Society Reviews 48 (2019): 3406–3424, 10.1039/C9CS00203K. 31150035

[9] B. S. Lukyanov and M. B. Lukyanova , “Spiropyrans: Synthesis, Properties, and Application. (Review),” Chemistry of Heterocyclic Compounds 41 (2005): 281–311, 10.1007/s10593-005-0148-x.

[10] B. Shao and I. Aprahamian , “Hydrazones as New Molecular Tools,” Chemistry 6 (2020): 2162–2173, 10.1016/j.chempr.2020.08.007.

[11] M. Clerc , S. Sandlass , O. Rifaie‐Graham , et al., “Visible Light‐Responsive Materials: The (Photo)Chemistry and Applications of Donor–Acceptor Stenhouse Adducts in Polymer Science,” Chemical Society Reviews 52 (2023): 8245–8294, 10.1039/d3cs00508a. 37905554 PMC10680135

[12] M. Bagheri , M. Mirzaee , S. Hosseini , and P. Gholamzadeh , “The Photochromic Switchable Imidazoles: Their Genesis, Development, Synthesis, and Characterization,” Dyes and Pigments 203 (2022): 110322, 10.1016/j.dyepig.2022.110322.

[13] Y. Kobayashi and J. Abe , “Recent Advances in Low‐power‐threshold Nonlinear Photochromic Materials,” Chemical Society Reviews 51 (2022): 2397–2415, 10.1039/d1cs01144h. 35262107

[14] T. Tran Ngoc , N. Grabicki , E. Irran , O. Dumele , and J. F. Teichert , “Photoswitching Neutral Homoaromatic Hydrocarbons,” Nature Chemistry 15 (2023): 377–385, 10.1038/s41557-022-01121-w. PMC9986110 36702883

[15] V. X. Truong and C. Barner‐Kowollik , “Photodynamic Covalent Bonds Regulated by Visible Light for Soft Matter Materials,” Trends in Chemistry 4 (2022): 291–304, 10.1016/j.trechm.2022.01.011.

[16] S. Wiedbrauk and H. Dube , “Hemithioindigo—An Emerging Photoswitch,” Tetrahedron Letters 56 (2015): 4266–4274, 10.1016/j.tetlet.2015.05.022.

[17] C. Petermayer and H. Dube , “Indigoid Photoswitches: Visible Light Responsive Molecular Tools,” Accounts of Chemical Research 51 (2018): 1153–1163, 10.1021/acs.accounts.7b00638. 29694014

[18] G. Kaplan , Z. Seferoğlu , and D. V. Berdnikova , “Photochromic Derivatives of Indigo: Historical Overview of Development, Challenges and Applications,” Beilstein Journal of Organic Chemistry 20 (2024): 228–242, 10.3762/bjoc.20.23. 38352070 PMC10862137

[19] A. Gernet , L. Toursel , L. M. Balivet , L. Pagès , and L. Jean , “Hemiindigoids as Prominent Photoswitch Scaffolds,” ChemPhotoChem 9 (2024): e202400261, 10.1002/cptc.202400261.

[20] D. Bléger and S. Hecht , “Visible‐Light‐Activated Molecular Switches,” Angewandte Chemie International Edition 54 (2015): 11338–11349, 10.1002/ange.201500628. 26096635

[21] Z. Zhang , W. Wang , M. O'Hagan , J. Dai , J. Zhang , and H. Tian , “Stepping out of the Blue: From Visible to Near‐IR Triggered Photoswitches,” Angewandte Chemie International Edition 61 (2022): e202205758, 10.1002/anie.202205758. 35524420

[22] M. Dong , A. Babalhavaeji , S. Samanta , A. A. Beharry , and G. A. Woolley , “Red‐Shifting Azobenzene Photoswitches for In Vivo Use,” Accounts of Chemical Research 48 (2015): 2662–2670, 10.1021/acs.accounts.5b00270. 26415024

[23] J. Andréasson and U. Pischel , “Molecules With a Sense of Logic: A Progress Report,” Chemical Society Reviews 44 (2015): 1053–1069, 10.1039/c4cs00342j. 25520053

[24] J. Andréasson and U. Pischel , “Molecules for Security Measures: From Keypad Locks to Advanced Communication Protocols,” Chemical Society Reviews 47 (2018): 2266–2279, 10.1039/c7cs00287d. 29487931

[25] P. Lu , D. Ahn , R. Yunis , et al., “Wavelength‐selective Light‐Matter Interactions in Polymer Science,” Matter 4 (2021): 2172–2229, 10.1016/j.matt.2021.03.021.

[26] A. Goulet‐Hanssens , F. Eisenreich , and S. Hecht , “Enlightening Materials With Photoswitches,” Advanced Materials 32 (2020): e1905966, 10.1002/adma.201905966. 31975456

[27] Q. Qi , J. T. Plank , A. R. Lippert , and I. Aprahamian , “A Photoswitchable Handheld Volumetric 3D Display,” Chemistry 10 (2024): 3575–3581, 10.1016/j.chempr.2024.07.012.

[28] J. Broichhagen , J. A. Frank , and D. Trauner , “A Roadmap to Success in Photopharmacology,” Accounts of Chemical Research 48 (2015): 1947–1960, 10.1021/acs.accounts.5b00129. 26103428

[29] K. Hüll , J. Morstein , and D. Trauner , “In Vivo Photopharmacology,” Chemical Reviews 118 (2018): 10710–10747, 10.1021/acs.chemrev.8b00037. 29985590

[30] M. M. Lerch , M. J. Hansen , G. M. van Dam , W. Szymanski , and B. L. Feringa , “Emerging Targets in Photopharmacology,” Angewandte Chemie International Edition 55 (2016): 10978–10999, 10.1002/anie.201601931. 27376241

[31] W. A. Velema , W. Szymanski , and B. L. Feringa , “Photopharmacology: Beyond Proof of Principle,” Journal of the American Chemical Society 136 (2014): 2178–2191, 10.1021/ja413063e. 24456115

[32] M. J. Fuchter , “On the Promise of Photopharmacology Using Photoswitches: A Medicinal Chemist's Perspective,” Journal of Medicinal Chemistry 63 (2020): 11436–11447, 10.1021/acs.jmedchem.0c00629. 32511922

[33] C. Brieke , F. Rohrbach , A. Gottschalk , G. Mayer , and A. Heckel , “Light‐Controlled Tools,” Angewandte Chemie International Edition 51 (2012): 8446–8476, 10.1002/anie.201202134. 22829531

[34] J. Bargstedt , M. Reinschmidt , L. Tydecks , T. Kolmar , C. M. Hendrich , and A. Jäschke , “Photochromic Nucleosides and Oligonucleotides,” Angewandte Chemie International Edition 63 (2024): e202310797, 10.1002/anie.202310797. 37966433

[35] S. Jia and E. M. Sletten , “Spatiotemporal Control of Biology: Synthetic Photochemistry Toolbox With Far‐Red and Near‐Infrared Light,” Acs Chemical Biology 17 (2022): 3255–3269, 10.1021/acschembio.1c00518. 34516095 PMC8918031

[36] R. Göstl , A. Senf , and S. Hecht , “Remote‐controlling Chemical Reactions by Light: Towards Chemistry With High Spatio‐Temporal Resolution,” Chemical Society Reviews 43 (2014): 1982–1996, 10.1039/c3cs60383k.. 24413363

[37] R. Dorel and B. L. Feringa , “Photoswitchable Catalysis Based on the Isomerisation of Double Bonds,” Chemical Communications 55 (2019): 6477–6486, 10.1039/c9cc01891c. 31099809

[38] P. Lentes , E. Stadler , F. Röhricht , et al., “Nitrogen Bridged Diazocines: Photochromes Switching Within the Near‐Infrared Region With High Quantum Yields in Organic Solvents and in Water,” Journal of the American Chemical Society 141 (2019): 13592–13600, 10.1021/jacs.9b06104. 31365240

[39] K. Klaue , Y. Garmshausen , and S. Hecht , “Taking Photochromism Beyond Visible: Direct One‐Photon NIR Photoswitches Operating in the Biological Window,” Angewandte Chemie International Edition 57 (2018): 1414–1417, 10.1002/anie.201709554. 29243389

[40] C. Y. Huang , A. Bonasera , L. Hristov , et al., “N,N ′‐Disubstituted Indigos as Readily Available Red‐Light Photoswitches With Tunable Thermal Half‐Lives,” Journal of the American Chemical Society 139 (2017): 15205–15211, 10.1021/jacs.7b08726. 29019401

[41] C. D. Huang and S. Hecht , “A Blueprint for Transforming Indigos to Photoresponsive Molecular Tools,” Chemistry – A European Journal 29 (2023): e202300981, 10.1002/chem.202300981. 37099319

[42] K. Klaue , W. Han , P. Liesfeld , F. Berger , Y. Garmshausen , and S. Hecht , “Donor–Acceptor Dihydropyrenes Switchable With Near‐Infrared Light,” Journal of the American Chemical Society 142 (2020): 11857–11864, 10.1021/jacs.0c04219. 32476422

[43] Z. Li , J. R. Zhang , X. K. Tian , et al., “Green‐/NIR‐light‐controlled Rapid Photochromism Featuring Reversible Thermally Activated Delayed Fluorescence and Photoelectronic Switching,” Chemical Science 13 (2022): 9381–9386, 10.1039/d2sc02662g. 36093018 PMC9383870

[44] Y. Wang , Y. Tang , Y. Zheng , et al., “A Visible and NIR Light‐Driven Photoswitch With High Fluorescence On/Off Contrast and Near‐Quantitative Cyclization Yield for Photonic Applications,” Advanced Functional Materials 36 (2025): e25706, 10.1002/adfm.202525706.

[45] Z. Li , X. Ma , J. Song , et al., “570 nm/770 nm Light‐Excited Deep‐Red Fluorescence Switch Based on Dithienylethene Derived From BF_2_‐Curcuminoid,” Chemical Science 16 (2025): 1762–1771, 10.1039/d4sc05473c. 39720129 PMC11664253

[46] W. R. Brode , E. G. Pearson , and G. M. Wyman , “The Relation Between the Absorption Spectra and the Chemical Constitution of Dyes. XXVII. Cis‐Trans Isomerism and Hydrogen Bonding in Indigo Dyes,” Journal of the American Chemical Society 76 (1954): 1034–1036, 10.1021/ja01633a033.

[47] J. Weinstein and G. M. Wyman , “Spectroscopic Studies on Dyes. II. The Structure of N,N'‐Dimethylindigo,” Journal of the American Chemical Society 78 (1956): 4007–4010, 10.1021/ja01597a038.

[48] L. A. Huber , P. Mayer , and H. Dube , “Photoisomerization of Mono‐Arylated Indigo and Water‐Induced Acceleration of Thermal Cis‐to‐Trans Isomerization,” ChemPhotoChem 2 (2018): 458–464, 10.1002/cptc.201700228.

[49] G. M. Wyman and W. R. Brode , “The Relation Between the Absorption Spectra and the Chemical Constitution of Dyes XXII. Cis‐Trans Isomerism in Thioindigo Dyes,” Journal of the American Chemical Society 73 (1951): 1487–1493, 10.1021/ja01148a023.

[50] V. A. Izmail'skii and M. A. Mostoslavskii , “Absorption Spectra of 3‐Oxo‐2,3‐Dihydrothianaphthene and Its Derivatives. II. Isomerism of 2‐Benzylidene‐3‐Oxo‐2,3‐Dihydrothionaphthene,” Ukr Khem Zh 27 (1961): 234–237.

[51] V. Josef , F. Hampel , and H. Dube , “Heterocyclic Hemithioindigos: Highly Advantageous Properties as Molecular Photoswitches,” Angewandte Chemie International Edition 61 (2022): e202210855, 10.1002/anie.202210855. 36040861 PMC9826360

[52] M. A. Mostoslavskii , M. D. Kravchenko , and I. N. Shevchuk , “Spectra of 3‐Keto‐2,3‐Dihydrothionaphthene and Its Derivatives. XI. Infrared Spectra and Spatial Structure of Some Indogenides and Thioindogenides,” Zhurnal Fizicheskoi Khimii 44 (1970): 1008–1012.

[53] C. Petermayer , S. Thumser , F. Kink , P. Mayer , and H. Dube , “Hemiindigo: Highly Bistable Photoswitching at the Biooptical Window,” Journal of the American Chemical Society 139 (2017): 15060–15067, 10.1021/jacs.7b07531. 28944664

[54] D. V. Berdnikova , “Aurones: Unexplored Visible‐Light Photoswitches for Aqueous Medium,” Chemistry – A European Journal 30 (2024): e202304237, 10.1002/chem.202304237. 38302861

[55] P. Friedländer and N. Woroshzow , “Über Thioindigofarbstoffe der Naphthalinreihe,” Justus Liebigs Annalen Der Chemie 388 (1912): 1–23, 10.1002/jlac.19123880102.

[56] P. Friedländer and A. Simon , “Über die Einwirkung von Schwefelchlorür auf Anthracen,” Berichte der Deutschen Chemischen Gesellschaft 55 (1922): 3969–3980, 10.1002/cber.19220551142.

[57] M. A. Mostoslavskii , V. A. Izmail'skii , and M. M. Shapkina , “Effect of Solvents on the Processes of Photochemical and Thermal Cis‐trans Isomerization of Perinaphthioindigo,” Zh Vses Khim O‐va im 7 (1962): 108–109.

[58] T. Takahashi , Y. Taniguchi , K. Umetani , H. Yokouchi , M. Hashimoto , and T. Kano , “Cis‐Trans Photoisomerization of Perinaphthothioindigo for Use as a Photo‐Imaging Sensor Using Fluorescence Under He‐Ne Laser Excitation,” Japanese Journal of Applied Physics 24 (1985): 173, 10.1143/JJAP.24.173.

[59] K. Fukunishi , M. Kobayashi , A. Morimoto , M. Kuwabara , H. Yamanaka , and M. Nomura , “Cis/Trans Isomerization of Perinaphthothioindigo Dye Adsorbed on Silica Gel,” Bulletin of the Chemical Society of Japan 62 (1989): 3733–3735, 10.1246/bcsj.62.3733.

[60] A. G. Lvov and D. V. Berdnikova , “Rubizhne Institute—A Birthplace of Photochromic Molecules,” Chemical Record 24 (2024): e202400143, 10.1002/tcr.202400143. 39491506

[61] L. Köttner , E. Ciekalski , and H. Dube , “Peri‐Anthracenethioindigo: A Scaffold for Efficient All‐Red‐Light and Near‐Infrared Molecular Photoswitching,” Angewandte Chemie International Edition 62 (2023): e202312955, 10.1002/anie.202312955. 37806956

[62] M. Hartinger , M. Herm , C. Schüßlbauer , et al., “From Triplet to Twist: The Photochemical E/Z ‐Isomerization Pathway of the Near‐Infrared Photoswitch Peri ‐Anthracenethioindigo,” Angewandte Chemie International Edition 64 (2025): e202510626, 10.1002/anie.202510626. 40548828 PMC12435429

[63] V. D. Paramonov , M. A. Mostoslavskii , and I. N. Shevchuk , “Effect of Naphthalene Fragment Annelation on the Absorption Spectrum and Quantum Yield of Photoisomerization of Perinaphththioindigo,” Zh Phiz Khim 33 (1978): 2676–2677.

[64] L. Köttner and H. Dube , “Path‐Independent All‐Visible Orthogonal Photoswitching for Applications in Multi‐Photochromic Polymers and Molecular Computing,” Angewandte Chemie International Edition 63 (2024): e202409214, 10.1002/anie.202409214. 38958439

[65] L. Köttner , F. Wolff , P. Mayer , E. Zanin , and H. Dube , “Rhodanine‐Based Chromophores: Fast Access to Capable Photoswitches and Application in Light‐Induced Apoptosis,” Journal of the American Chemical Society 146 (2024): 1894–1903, 10.1021/jacs.3c07710.. 38207286

[66] S. K. Patel , J. Cao , and A. R. Lippert , “A Volumetric Three‐dimensional Digital Light Photoactivatable Dye Display,” Nature Communications 8 (2017): 15239, 10.1038/ncomms15239. PMC5508202 28695887

[67] S. Toyota , S. Ban , M. Hara , M. Kawamura , H. Ikeda , and E. Tsurumaki , “Synthesis and Properties of Rubicene‐Based Aromatic π‐Conjugated Compounds as Five‐Membered Ring Embedded Planar Nanographenes,” Chemistry – A European Journal 29 (2023): e202301346, 10.1002/chem.202301346. 37278362

[68] J. J. van der Wal , J. D. Steen , A.‐K. Rückert , et al., “Acid‐ and Nucleophile‐Gated Photoisomerization of Phosphaindirubin,” Angewandte Chemie International Edition 65 (2026): e19686, 10.1002/anie.202519686. 41320767 PMC12955533

