# Supplementary material for: A Reversible 900 nm Near‐Infrared Photoswitch for Invisibly Writable 2D and 3D Displays
Source: Angew Chem Int Ed Engl. 2026 Jun 10;65(31):e5802775. doi: 10.1002/anie.5802775 (PMC13411626; doi:10.1002/anie.5802775)
Supplement: Supplementary file 2 — Supporting File 2: anie72953‐sup‐0002‐SuppMat.pdf. [file ANIE-65-e5802775-s001.pdf]

Supporting Information

A Reversible 900 nm Near-Infrared Photoswitch for Invisibly Writable 2D and 3D Displays

Elias Ciekalski,[a] *Henry Dube* [a]*

[a] Friedrich-Alexander-Universität Erlangen-Nürnberg, Department of Chemistry and Pharmacy, Nikolaus-Fiebiger-Str. 10, 91058 Erlangen, Germany.

* E-mail: henry.dube@fau.de

Table of Contents

Materials and general methods 2
Synthesis of PBFT 4
9-Bromo-2,6-di-*tert*-butyl-10-(3,5-di-*tert*-butylphenyl)anthracene[S1] (2) 5
8-Bromo-2,4,6,11-tetra-*tert*-butylbenzo[*a*]aceanthrylene[S1] (3) 6
Methyl 2-((2,4,6,11-tetra-tert-butylbenzo[*a*]aceanthrylen-8-yl)thio)acetate[S3] (4)...... 7
2-((2,4,6,11-Tetra-tert-butylbenzo[*a*]aceanthrylen-8-yl)thio)acetic acid (5)................ 8
2,8,11,13-Tetra-tert-butylbenzo[*de*]fluoreno[9,1-gh]thiochromen-6(5H)-one[S4] (6) ... 9
2,2',8,8',11,11',13,13'-Octa-tert-butyl-[5,5'-bibenzo[*de*]fluoreno[9,1-gh]thiochromene]-6,6'(5H,5'H)-dione (7a, 7b) 10
Peri-benzo[*a*]fluoranthenthioindigo (PBFT).. 11
Thermal isomerization of PBFT 13
Photophysical and photochemical properties of PBFT 25
Isomer composition in the pss monitored by ^{1}H NMR spectroscopy 25
Isomer composition in the pss monitored by UV/Vis spectroscopy 27
Molar absorption coefficients of PBFT 29
Quantum Yield Determination of PBFT 32
Photoisomerization of PBFT followed by UV/Vis spectroscopy.. 35
Photostability of PBFT 36
Photoisomerization of PBFT with 905 nm NIR light.. 39
Incorporation of PBFT into transparent 2D and 3D materials and reversible information inscription 40
NMR spectra.. 48
References 59

Materials and general methods

Solvents and reagents were obtained from BLDPharm, Sigma-Aldrich, abcr, Fischer Scientific, or TCI in the qualities puriss., p.a., or purum and used as received. Technical grade solvents were purified by distillation on a rotary evaporator (Heidolph Hei-Vap Expert). Reaction monitoring and chromatography fraction analysis was done using thin layer chromatography (TLC) on Supelco Silica 60 F_{254} TLC plates and detected under irradiation with UV light (254 nm or 366 nm). For display and optical memory applications commercially available Rayher Hobby 3130200 Candle Gel was used.

Reactions using dry solvents were carried out under nitrogen atmosphere. Dry Schlenk flasks were evacuated and backfilled with nitrogen three times to preserve dry and air-free conditions. Syringes and needles used for dry solvents were flushed with nitrogen prior use. Degassing of solvents was achieved by nitrogen bubbling for 15 minutes. Reactions not requiring water-free conditions were executed under atmospheric conditions.

Medium pressure liquid chromatography was performed on a Biotage Selekt medium pressure liquid chromatography system. Separation was achieved either on silica gel 60 (Macherey-Nagel Silica 60 M, 0.04 – 0.063 mm) using Macherey-Nagel Chromabond Flash DL empty columns or prepacked Chromabond Flash RS SiOH cartridges.

High performance liquid chromatography was carried out on a Shimadzu HPLC system equipped with a LC-20AP solvent delivery module, CTO-20A column oven, a SPDM20A photodiode array UV-Vis detector, and a CBM-20A system controller using a preparative (SiOH, 110 Å pore size, 5 µm particle size, 21 mm x 250 mm) silica column.

Supercritical fluid chromatography was done on a Waters SFC Prep 150 AP system with UV-Vis triggered (Waters 2998 Photodiode Array Detector) purification using a Waters Viridis Silica 2-Ethylpyridine OBD Prep Column (2-ethylpyridine silane, 100 Å pore size, 5 µm particle size, 10 mm x 150 mm) silica column.

Mass spectrometry was conducted on a Bruker microTOF II device (APPI, ESI) or Bruker ultrafleXtreme (MALDI-TOF). Found masses from high resolution mass spectrometry experiments are given in m/z units.

Infrared spectra were recorded on a Thermo Scientific Nicolet Summit X FTIR Spectrometer equipped with the Everest ATR Accessory.

^{1}H NMR and ^{13}C NMR spectra were measured on a Bruker Avance NEO HD 400 MHz, Bruker Avance Neo HDX 500 MHz, or Bruker Avance Neo HDX 600 MHz NMR spectrometer. Deuterated NMR spectroscopy solvents were obtained from Sigma-Aldrich and used without further purification. Tetramethylsilan (TMS) was used as the external standard; chemical shifts (*δ*) are reported in parts per million (ppm) relative to TMS. The residue solvent signals were used as internal reference for the following solvents: C_6D_6: δ_H = 7.16 ppm, δ_C = 128.06 ppm; CD_2Cl_2: δ_H = 5.32 ppm, δ_C = 53.84 ppm; $CDCl_3$: δ_H = 7.26 ppm, δ_C = 77.16 ppm; DMSO-d_5: δ_H = 2.50 ppm, δ_C = 39.52 ppm. Resonance multiplicity is given as follows: s (singlet), d (doublet), t (triplet), m (multiplet).

Photoisomerization experiments were carried out using LEDs from Thorlabs Inc. and Roithner Lasertechnik GmbH (905 nm, 880 nm, 850 nm, 830 nm, 810 nm, 780 nm, 730 nm, 680 nm, 650 nm, 625 nm, 595 nm, 565 nm) and a DARKBEAM ir Flashlight (Infrared 850nm Lights LED Mini, 5W) for irradiation.

UV/VIS spectra were recorded on an Agilent Cary 60 UV-VIS spectrometer using spectral grade solvents from Sigma-Aldrich. Molar absorption coefficients (*ε*) are given in $L\cdot mol^{-1}\cdot cm^{-1}$, absorption wavelengths (*λ*) in nm.

Synthesis of PBFT

9,10-Dibromo-2,6-di-*tert*-butylanthracene **1** was prepared according to published procedures.[S1] Synthesis of **2** and **3** also followed published protocols.[S2] Synthesis of **4** was achieved following a modified procedure from literature.[S3]

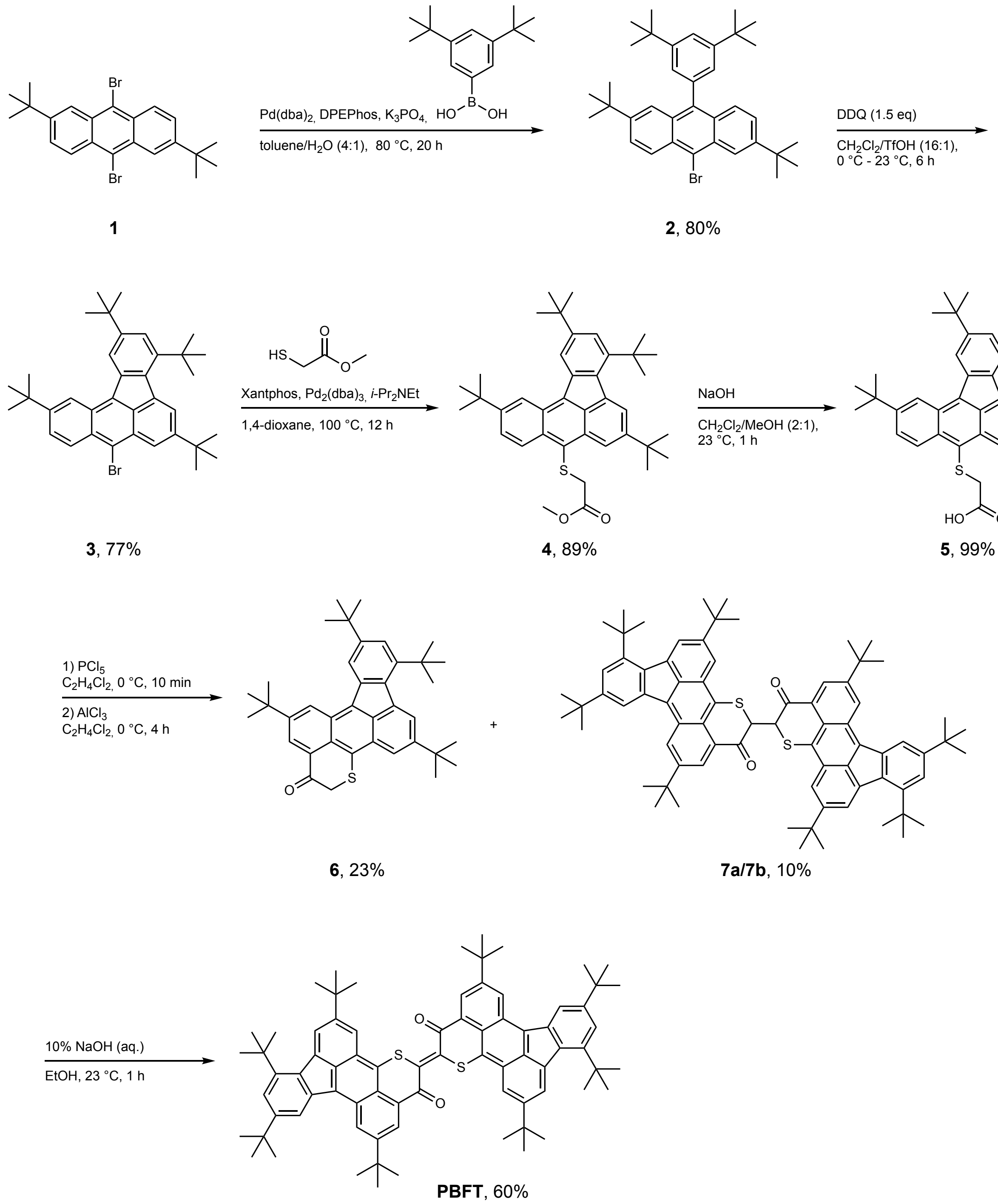

Scheme S1 Synthesis route for **PBFT** starting from anthracene **1**.

9-Bromo-2,6-di-*tert*-butyl-10-(3,5-di-*tert*-butylphenyl)anthracene[S1] (2)

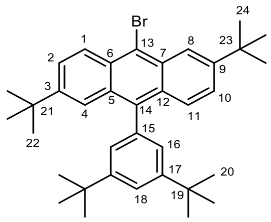

A dry Schlenk flask containing a mixture of 9,10-dibromo-2,6-di-*tert*-butylanthracene **1** (2.00 g, 4.46 mmol, 2.0 equiv.), (3,5-di-*tert*-butylphenyl)boronic acid (677 mg, 2.23 mmol, 1.0 equiv.), K_3PO_4 (1.42 g, 6.69 mmol, 3.0 equiv.), $Pd(dba)_2$ (257 mg, 446 µmol, 0.20 equiv.), and DPEPhos (300 mg, 558 µmol, 0.25 equiv.) was evacuated and backfilled with nitrogen three times. A nitrogen-purged mixture of toluene (40 mL) and H_2O (10 mL) was added to the flask, and the resulting suspension was purged with nitrogen for additional 15 min. The reaction mixture was stirred at 80 °C for 20 h. After letting the mixture cool down to 23 °C, it was poured into water (100 mL) and extracted with CH_2Cl_2 (3 x 100 mL). The combined organic phases were dried over $MgSO_4$, concentrated *in vacuo* and purified using column chromatography (SiO_2, *iso*-hexane) to afford **2** as slightly yellow solid (1.98 g, 3.55 mmol, 80%).

^{1}H NMR (400 MHz, CDCl3) *δ* (ppm) = 8.52 (dd, *J* = 9.3, 0.6 Hz, 1H, H-C(1)), 8.48 (dd, *J* = 2.0, 0.6 Hz, 1H, H-C(8)), 7.72 (dd, *J* = 9.2, 0.6 Hz, 1H, H-C(11)), 7.67 (dd, *J* = 9.3, 2.0 Hz, 1H, H-C(2)), 7.58 – 7.52 (m, 2H, H-C(4), H-C(18)), 7.47 (dd, *J* = 9.2, 2.0 Hz, 1H, H-C(10)), 7.25 (d, *J* = 1.8 Hz, 2H, H-C(16)), 1.48 (s, 9H, H-C(24)), 1.39 (s, 18H, H-C(20)), 1.27 (s, 9H, H-C(22)).

^{13}C NMR (101 MHz, CDCl3) *δ* (ppm) = 150.7 (C(17)), 149.1 (C(9)), 147.3 (C(3)), 138.5 (C(14)), 137.6 (C(15)), 131.0 (C(5)), 129.9 (C(7)), 129.8 (C(12)), 129.2 (C(6)), 127.5 (C(1), C(11)), 126.3 (C(2)), 125.8 (C(16)), 124.9 (C(10)), 122.3 (C(10)), 122.1 (C(8)), 121.9 (C(13)), 121.0 (C(4), C(18)), 35.4 (C(23)), 35.1 (C(19)), 35.0 (C(21)), 31.7 (C(20)), 31.1 (C(24)), 30.9 (C(22)).

R_f = 0.60 (SiO_2, *iso*-hexane).

MS (APPI), $[MH]^+$: m/z calc.: 557.2778 for $[C_{36}H_{46}Br]^+$, found: 557.2794.

8-Bromo-2,4,6,11-tetra-*tert*-butylbenzo[*a*]aceanthrylene[S1] (3)

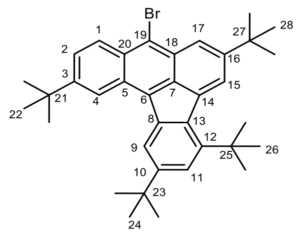

2 (400 mg, 717 µmol) was dissolved in nitrogen-purged CH_2Cl_2 (100 mL) and cooled to 0 °C. DDQ (244 mg, 1.08 mmol, 1.5 equiv.) was added and the reaction mixture stirred for 10 min at 0 °C. TfOH (7 mL) was added and the reaction stirred without additional cooling for 6 h. The reaction was quenched with aqueous $NaHCO_3$ (150 mL) and extracted with CH_2Cl_2 (3 x 100 mL). The organic phases were combined, dried over Mg_2SO_4, and the solvent was removed under reduced pressure. Product **3** was purified using column chromatography (SiO_2, *iso*-hexane) and isolated as a yellow foam (298 mg, 536 µmol, 77%).

^{1}H NMR (400 MHz, CDCl3) *δ* (ppm) = 8.83 (d, *J* = 1.8 Hz, 1H, H-C(17)), 8.57 (d, *J* = 9.3 Hz, 1H), H-C(1), 8.47 (d, *J* = 1.2 Hz, 1H, H-C(17)), 8.45 (d, *J* = 1.7 Hz, 1H, H-C(4)), 8.17 (d, *J* = 1.2 Hz, 1H), 7.71 (dd, *J* = 9.4, 1.9 Hz, 1H), 7.52 (d, *J* = 1.6 Hz, 1H), 1.76 (s, 9H), 1.56 (s, 9H), 1.55 (s, 9H), 1.51 (s, 9H).

^{13}C NMR (101 MHz, $CDCl_3$) *δ* (ppm) = 151.0, 150.7, 149.7, 147.4, 142.4, 136.9, 133.2, 131.1, 131.0, 130.9, 129.2, 129.0, 128.4, 127.5, 125.8, 123.0, 122.2, 120.6, 119.4, 118.91, 36.3, 35.8, 35.4, 35.4, 31.7, 31.7, 31.4, 31.0, 30.3.

NMR data consistent with literature.[S2]

R_f = 0.50 (SiO_2, *iso*-hexane).

HRMS (APPI), $[MH]^+$: m/z calc.: 555.2621 for $[C36H_{44}Br]^+$, found: 555.2626.

Methyl 2-((2,4,6,11-tetra-tert-butylbenzo[*a*]aceanthrylen-8-yl)thio)acetate[S3] (4)

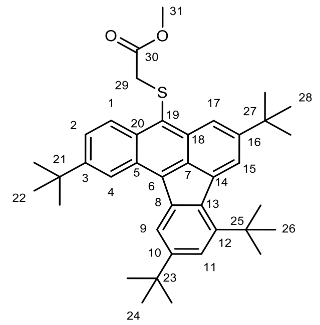

To a flame-dried Schlenk flask were added **3** (470 mg, 846 µmol, 1.0 equiv.), Xantphos (48.9 mg, 84.6 µmol, 0.10 equiv.), $Pd_2(dba)_3$ (46.5 mg, 50.8 µmol, 0.06 equiv.), methyl thioglycolate (90.8 µL, 1.02 mmol, 1.20 equiv.), *i*-Pr_2NEt (219 µL, 1.69 mmol, 2.0 equiv.), and dry 1,4-dioxane (20 mL) under nitrogen flow. After purging for 15 minutes with nitrogen, the reaction mixture was stirred at 100 °C for 12 h. The reaction mixture was cooled down to ambient temperature and poured into water (50 mL) and extracted with CH_2Cl_2 (3 x 30 mL). The crude product was purified by column chromatography (SiO_2, *iso*-hexane:EtOAc = 90:10) to afford ester **4** as a yellow resin (491 mg, 1.51 mmol, 89%).

^{1}H NMR (400 MHz, $CDCl_3$) δ (ppm) = 8.89 (d, *J* = 9.4 Hz, 1H, H-C(1)), 8.81 (d, *J* = 2.0 Hz, 1H, H-C(4)), 8.46 (d, *J* = 1.2 Hz, 1H, H-C(17))), 8.42 (d, *J* = 1.7 Hz, 1H, H-C(15)), 8.40 (d, J = 1.3 Hz, 1H, H-C(9))), 7.66 (dd, *J* = 9.3, 1.9 Hz, 1H, H-C(2)), 7.46 (d, *J* = 1.7 Hz, 1H, H-C(11)), 3.51 (s, 2H, H-C(29)), 3.28 (s, 3H, H-C(31)), 1.70 (s, 9H, H-C(26)), 1.49 (s, 9H, H-C(28)), 1.48 (s, 9H, H-C(22)), 1.44 (s, 9H, H-C(24)).

^{13}C NMR (101 MHz, $CDCl_3$) δ (ppm) = 170.4 (C(30)), 150.8 (C(16)), 150.6 (C(10)), 149.3 (C(3)), 147.3 (C(12)), 142.4 (C(8) or C(14)), 137.2 (C(18)), 135.1 (C(20)), 133.7 (C(13)), 133.4 (C(6)), 131.2 (C(8) or C(14)), 130.5 (C(7)), 128.7 (C(1)), 128.3 (C(15)), 127.9 (C(5)), 127.8 (C(19)), 125.6 (C(2)), 122.5 (C(11)), 119.8 (C(17)), 119.7 (C(9)), 119.3 (C(4)), 52.2 (C(31)), 39.1 (C(29)), 36.2 (C(27)), 35.8 (C(25)), 35.4 (C(21)), 35.4 (C(23)), 31.7 (C(24)), 31.5 (C(28)), 31.1 (C(22)), 30.3 (C(26)).

R_f = 0.60 (SiO_2, *iso*-hexane:EtOAc = 9:1).

HRMS (APPI), $[MH]^+$: m/z calc.: 581.3448 for $[C_{39}H_{49}O_2S]^+$, found: 581.3451.

2-((2,4,6,11-Tetra-tert-butylbenzo[*a*]aceanthrylen-8-yl)thio)acetic acid (5)

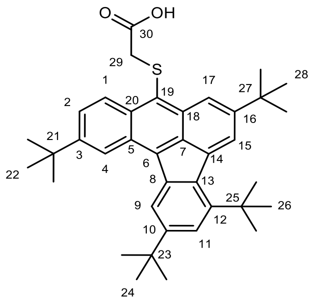

Ester **4** (877 mg, 1.51 mmol) was dissolved in CH_2Cl_2 (30 mL) and a solution of NaOH (0.5 N) in MeOH (15 mL) was added. The mixture was stirred at 23 °C for 1 h. The reaction was quenched by the addition of a saturated solution of aqueous NH_4Cl (50 mL). The product was extracted with CH_2Cl_2 (3 x 50 mL). The combined organic phases were adsorbed on silica and impurities were removed with CH_2Cl_2 over a short silica plug. The residue was washed off the silica plug using MeOH and the obtained product solution was concentrated *in vacuo*. Product **5** was afforded as an orange solid (856 mg, 1.50 mmol, 99%).

^{1}H NMR (400 MHz, DMSO-d_6) δ (ppm) = 8.95 (d, J = 9.4 Hz, 1H, H-C(1)), 8.78 (d, J = 1.9 Hz, 1H, H-C(4)), 8.57 (d, J = 1.1 Hz, 1H, H-C(17)), 7.88 (dd, J = 9.4, 1.8 Hz, 1H, H-C(2)), 7.52 (d, J = 1.5 Hz, 1H, H-C(11)), 3.57 (s, 2H, H-C(29)), 1.71 (s, 9H, H-C(26)), 1.52 (s, 9H, H-C(28)), 1.51 (s, 9H, H-C(22)), 1.47 (s, 9H, H-C(24)).

^{13}C DEPTQ NMR (101 MHz, DMSO-d_6) δ (ppm) = 170.6 (C(30)), 150.6 (C(10)), 150.3 (C(16)), 149.9 (C(3)), 147.2 (C(12)), 141.5 (C(14) or C(8)), 136.2 (C(18)), 134.4 (C(20)), 132.6 (C(13)), 131.5 (C(6)), 130.4 (C(14) or C(8)), 129.8 (C(19)), 129.5 (C(7)), 128.2 (C(1)), 128.1 (C(15)), 127.9 (C(5)), 126.1 (C(2)), 122.4 (C(11)), 120.2 (C(17)), 119.1 (C(9)), 118.3 (C(4)), 40.8 (C(29)), 35.9 (C(27)), 35.4 (C(25)), 35.2 (C(21)), 35.0 (C(23)), 31.3 (C(24)), 31.1 (C(28)), 30.6 (C(22)), 29.8 (C(26)).

R_f = 0.66 (SiO_2, CH_2Cl_2:MeOH = 9:1), 0.00 (SiO_2, *iso*-hexane:EtOAc = 9:1).

HRMS (APPI), $[MH]^+$: m/z calc.: 567.3291 for $[C_{38}H_{47}O_2S]^+$, found: 567.3302.

2,8,11,13-Tetra-tert-butylbenzo[*de*]fluoreno[9,1-gh]thiochromen-6(5H)-one[S4] (6)

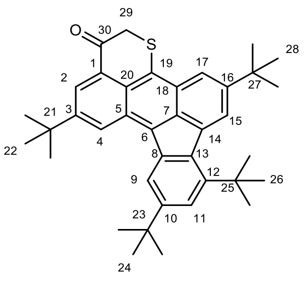

Acid **5** (150 mg, 265 µmol, 1.0 equiv.) was suspended in dry $C_2H_4Cl_2$ (15 mL) and PCl_5 (110 mg, 523 µmol, 2.0 equiv.) was added at 0° C. The conversion to the acyl chloride was monitored by TLC: a small sample of reaction mixture was taken out and mixed with a small amount of methanol to immediately form the ester **4**. After 10 min, full conversion to the acyl chloride was confirmed. $AlCl_3$ (52.9 mg, 397 µmol, 1.5 equiv.) was added at 0 °C and the reaction stirred at this temperature for 1 h. Subsequently, $AlCl_3$ (17.6 mg, 132 µmol, 0.5 equiv.) was added after 1 h. Additional $AlCl_3$ (17.6 mg, 132 µmol, 0.5 equiv.) was added 1 h after the last addition and the reaction was stirred for 1 h. The reaction was quenched with water and extracted with CH_2Cl_2 (3 x 50 mL). The combined organic phases were dried over $MgSO_4$ and the solvent removed under reduced pressure. The crude product was either separated via HPLC (SiO_2, *iso*-hexane:EtOAc = 99:1) or used in the next step after purification on MPLC (SiO_2, *iso*-hexane:EtOAc = 100:0 to 90:10). Product **5** was afforded as a bright red-orange solid (33.2 mg, 60.5 µmol, 23%).

^{1}H NMR (400 MHz, $CDCl_3$) δ (ppm) = 9.14 (d, J = 2.1 Hz, 1H, H-C(4)), 8.51 (d, J = 1.2 Hz, 1H, H-C(15)), 8.40 (d, J = 2.0 Hz, 1H, H-C(2)), 8.39 (d, J = 1.7 Hz, 1H, H-C(9)), 7.53 (d, J = 1.7 Hz, 1H, H-C(17)), 7.53 (d, J = 1.7 Hz, 1H, H-C(11)), 3.94 (s, 2H, H-C(29)), 1.76 (s, 9H, H-C(26)), 1.56 (s, 9H, H-C(28)), 1.55 (s, 9H, H-C(22)), 1.52 (s, 9H, H-C(24)).

^{13}C NMR (101 MHz, $CDCl_3$) δ (ppm) = 191.4 (C(30)), 150.9 (C(16)), 150.8 (C(10)), 148.7 (C(3)), 147.6 (C(12)), 142.4 (C(8)), 137.1 (C(14)), 133.0 (C(13)), 130.6 (C(1)), 130.2 (C(7)), 130.1 (C(6)), 129.0 (C(15)), 128.5 (C(20)), 128.5 (C(5) or C(18)), 126.9 (C(19)), 126.6 (C(2)), 126.6 (C(5) or C(18)), 126.2 (C(4)), 122.3 (C(11)), 119.1 (C(9)), 117.5 (C(17)), 36.3 (C(29)), 36.3 (C(27)), 35.8 (C(25)), 35.6 (C(21)), 35.4 (C(23)), 31.7 (C(24)), 31.4 (C(28)), 31.0 (C(22)), 30.3 (C(26)).

R_f = 0.33 (SiO2, *iso*-hexane : EtOAc 95:5).

HRMS (ESI), $[MNa]^+$: m/z calc.: 550.3006 for $[C_{38}H_{44}NaOS]^+$, found: 550.3020.

2,2',8,8',11,11',13,13'-Octa-tert-butyl-[5,5'-bibenzo[*de*]fluoreno[9,1-gh]thiochromene]-6,6'(5H,5'H)-dione (7a, 7b)

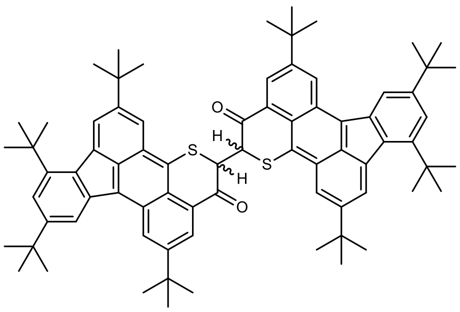

See synthesis of **6**, where **7a** and **7b** form as a side product. The formation of **7a** and **7b** could be reduced substantially by portion-wise addition of $AlCl_3$ (see procedure for **6**). Alternatively, the formation of **7a** and **7b** could be increased by adding 4.0 equiv. of $AlCl_3$ in one portion at 0 °C. The diastereomeric mixtures could be separated via HPLC (SiO_2, *iso*-hexane:EtOAc = 99:1) but no further analysis was pursued. Products **7a** and **7b** were afforded as dark red solids (10%).

7a:

^{1}H NMR (500 MHz, $CDCl_3$) *δ* (ppm) = 9.16 (d, *J* = 2.1 Hz, 1H), 8.48 (d, *J* = 1.3 Hz, 1H), 8.43 (d, *J* = 2.0 Hz, 1H), 8.39 (d, *J* = 1.7 Hz, 1H), 8.20 – 8.15 (m, 1H), 7.52 (d, *J* = 1.7 Hz, 1H), 4.72 (s, 1H), 1.75 (s, 9H), 1.59 (s, 9H), 1.56 (s, 9H), 1.52 (s, 9H).

^{13}C NMR (126 MHz, $CDCl_3$) *δ* (ppm) = 190.7, 151.1, 150.8, 149.0, 147.53, 142.3, 137.2, 133.0, 130.6, 130.5, 130.4, 129.1, 128.2, 128.1, 127.8, 127.1, 126.3, 124.8, 122.4, 119.2, 117.5, 47.8, 36.3, 35.8, 35.7, 35.4, 31.7, 31.7, 31.4, 31.3, 31.2, 31.2, 31.1, 31.0, 30.3.

R_f = 0.23 (SiO2, *iso*-hexane:EtOAc = 95:5).

7b:

^{1}H NMR (400 MHz, $CDCl_3$) *δ* (ppm) = 9.08 (d, *J* = 2.1 Hz, 1H), 8.46 – 8.44 (m, 1H), 8.32 (d, *J* = 1.6 Hz, 1H), 8.00 (d, *J* = 1.1 Hz, 1H), 7.48 (d, *J* = 1.6 Hz, 1H), 4.38 (s, 1H), 1.72 (s, 9H), 1.57 (s, 9H), 1.52 (s, 9H), 1.48 (s, 9H).

^{13}C NMR (101 MHz, $CDCl_3$) *δ* (ppm) = 189.9, 151.1, 150.8, 148.8, 147.5, 142.2, 137.1, 133.0, 130.7, 130.4, 130.1, 129.1, 128.0, 127.9, 127.8, 127.2, 126.2, 124.0, 122.4, 119.2, 117.5, 47.1, 36.3, 35.8, 35.7, 35.6, 35.3, 31.6, 31.6, 31.3, 31.2, 31.1, 30.2.

R_f = 0.20 (SiO_2, *iso*-hexane:EtOAc = 95:5).

HRMS (ESI), $[MH]^+$: m/z calc.: 1095.6142 for $[C_{76}H_{87}O_2S_2]^+$, found: 1095.6125; $[MNa]^+$: m/z calc.: 1117.5961 for $[C_{76}H_{86}NaO_2S_2]^+$, found: 1117.5983.

Peri-benzo[*a*]fluoranthenthioindigo (PBFT)

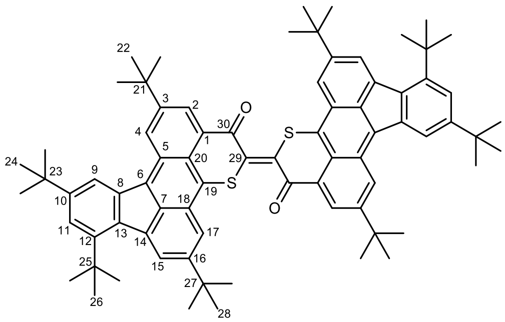

Monomer **6** (60.0 mg, 109 µmol, 2.0 equiv.) was dissolved in EtOH (7 mL) and an aqueous solution of NaOH (10% w/w, 7 mL, 18.9 mmol, 173 equiv.) was added. The reaction was stirred in an open flask for 1 h. **PBFT** precipitated from the solution as a brown solid, which was filtered off and washed thoroughly with methanol and ethyl acetate. If monomer **7** was purified, **PBFT** was obtained as a pure substance after filtration. If monomer **7** was not purified by column chromatography, the target molecule **PBFT** could be purified by SFC using a Waters Viridis Silica 2-Ethylpyridine OBD Prep Column (2-ethylpyridine silane, 100 Å pore size, 5 µm particle size, 10 mm x 150 mm, CO_2:CH_2Cl_2 = 8:2). **PBFT** was obtained as a brown solid (36.0 mg, 32.9 µmol, 60%).

E isomer

^{1}H NMR (400 MHz, CD_2Cl_2) δ (ppm) = 9.21 (d, J = 2.0 Hz, 1H, H-C(4)), 8.95 (d, J = 1.9 Hz, 1H H-C(2)), 8.57 (d, J = 1.2 Hz, 1H, H-C(15)), 8.46 (s, 1H, H-C(17)), 8.41 (d, J = 1.6 Hz, 1H, H-C(9)), 7.56 (d, J = 1.7 Hz, 1H, H-C(11)), 1.77 (s, 9H, H-C(26)), 1.66 (s, 9H, H-C(22)) 1.66 (s, 9H, H-C(28)), 1.53 (s, 9H, H-C(24)).

^{13}C(DEPTQ) NMR (151 MHz, CD_2Cl_2) δ (ppm) = 182.3 (C(30)), 151.6 (C(3) or C(12)), 151.4 (C(23)), 150.5 (C(16)), 148.0 (C(12) or C(3)), 142.4, 137.5, 133.2 (C(13)), 131.3, 130.8, 130.5 (C(7)), 130.0 (C(6)), 129.7 (C(2)), 129.4 (C(15)), 128.3 (C(1)), 126.6 (C(4)), 125.9 (C(19)), 125.7 (C(20)), 125.4, 122.9 (C(11)), 119.5 (C(9)), 116.1 (C(17)), 36.7 (C(27)), 36.0 (C(25)), 36.0 (C(21)), 35.6 (C(23)), 31.6 (C(24)), 31.5 (C(22)), 31.1 (C(28)), 30.3 (C(26)).

Quarternary carbons C5, C8, C14, C18, and C29 were not assigned due to no visible couplings.

R_f = 0.81 (SiO_2, *iso*-hexane:CH_2Cl_2 = 1:1).

HRMS (MALDI-TOF), $[MH]^+$: m/z calc.: 1092.5913 for [$C_{76}H_{84}O_2S_2$], found: 1092.5909.

Additional measurement in benzene-d_6:

^{1}H NMR (400 MHz, C_6D_6) δ (ppm) = 9.29 (d, J = 2.0 Hz, 1H), 9.18 (d, J = 1.9 Hz, 1H), 8.86 (d, J = 1.1 Hz, 1H), 8.67 (d, J = 1.2 Hz, 1H), 8.64 (d, J = 1.7 Hz, 1H), 7.72 (d, J = 1.6 Hz, 1H), 1.78 (s, 9H), 1.54 (s, 9H), 1.45 (s, 9H), 1.39 (s, 9H).

***Z* isomer**

^{1}H NMR (400 MHz, CD_2Cl_2) δ (ppm) = 9.22 (d, J = 2.0 Hz, 1H, H-C(4)), 8.69 (d, J = 1.9 Hz, 1H, H-C(2)), 8.57 (d, J = 1.2 Hz, 1H, H-C(15)), 8.44 (d, J = 1.5 Hz, 1H, H-C(9)), 8.29 (d, J = 1.1 Hz, 1H, H-C(17)), 7.57 (d, J = 1.6 Hz, 1H, H-(11)), 1.78 (s, 9H, H-C(26)), 1.64 (s, 18H, H-C(22)), 1.64 (s, 18H, H-C(28)), 1.53 (s, 9H, H-C(24)).

^{13}C(DEPTQ) NMR (151 MHz, CD_2Cl_2) δ (ppm) = 181.9, 151.8, 151.3, 150.1, 147.9, 142.2, 137.3, 132.9, 132.2, 131.5, 130.4, 130.4, 129.5, 128.9, 128.2, 126.0, 125.9, 125.2, 122.9, 119.5, 116.3, 36.5, 35.8, 35.4, 31.5, 31.1, 30.9, 30.1.

Additional measurement in benzene-d_6:

^{1}H NMR (400 MHz, C_6D_6) δ (ppm) = 9.28 (d, J = 2.1 Hz, 1H), 9.05 (d, J = 1.9 Hz, 1H), 8.66 (d, J = 1.1 Hz, 1H), 8.65 (s, 1H), 8.44 (d, J = 1.1 Hz, 1H), 7.73 (d, J = 1.6 Hz, 1H), 1.79 (s, 9H), 1.54 (s, 18H), 1.40 (s, 9H).

Thermal isomerization of PBFT

The thermal isomerization of the metastable *Z* isomer of **PBFT** to its stable *E* isomer occurs within minutes to hours at 22 °C. To investigate the thermal stability of the *Z* isomer, a sample was first enriched to the metastable *Z* isomer photochemically via NIR irradiation. The thermal back isomerization was then followed in distinct time intervals in the dark by either NMR spectroscopy in benzene-d_6 or by UV/Vis spectroscopy in benzene, CH_2Cl_2, pyridine, and THF solutions. From the decay kinetics, the first-order rate constant for the *Z* to *E* isomerization could be determined.

By inserting the rate constant *k* of the reaction into the *Eyring* equation (eq. 1), the Gibbs energy of activation $\Delta G^{\ddagger}$ can be obtained:

$$k = \frac{k_B\, T}{h} e^{\frac{-\Delta G^{*}}{R\, T}} \qquad \text{eq. 1}$$

With k = rate constant of the reaction

k_B = Boltzmann constant ($1.381 \cdot 10^{-23}$ J · K^{-1})

T = temperature in K

h = Planck constant ($6.626 \cdot 10^{-34}$ J · s)

Rearranging eq. 1 and inserting *k* gives the value for $\Delta G^{\ddagger}$:

$$\Delta G^{\ddagger}(in\, J\, mol^{-1}) = -\ln\left(\frac{k\, h}{k_B\, T}\right) R\, T \qquad \text{eq. 2}$$

Dividing ln(2) by the rate constant *k* gives the half-life time for the metastable *Z*-**1** isomer:

$$t_{1/2} = \frac{\ln(2)}{k} \qquad \text{eq. 3}$$

The relative energy difference ΔG between both isomers is obtained by inserting the equilibrium constant K = ([*Z*]/[*E*]) into eq. 4:

$$-\Delta G = \ln(K)\, R\, T \qquad \text{eq. 4}$$

For a lower limit, it was assumed that the NMR experiment does not observe 5% of the metastable *Z* isomer after thermal back-isomerization, which means $K = ([Z]/[E]) = (5/95)$. This rather conservative estimate leads to a value of $\Delta G = \geq 1.7$ kcal/mol.

Table S1 Gibbs energy of activation $\Delta G^{\ddagger}$ of the thermal isomerization of the *Z*-**PBFT** isomer to the *E*-**PBFT** isomer, the half-life $t_{1/2}$ of the *Z* isomer, and lower limit for the relative energy difference measured by UV/Vis spectroscopy. [a] measured by ^{1}H NMR spectroscopy, values in round brackets are linearly extrapolated to 25 °C.

	**$\Delta G^{\ddagger}$ (therm. *Z*/*E*)** [kcal mol^{-1}]	**T** [°C]	**$t_{1/2}$** [h]	**E_{end}** [%]	**ΔG** [kcal mol^{-1}]	**c** [mol L^{-1}]
benzene-d_6	22.5	15 (25)[a]	3.40 (0.90)[a]	≥95	≥1.7	~$5 \cdot 10^{-3}$
benzene	22.6	22	1.78	≥95	≥1.7	~$2 \cdot 10^{-3}$
CH_2Cl_2	22.0	22	0.61	≥95	≥1.7	~$2 \cdot 10^{-5}$
pyridine	21.6	22	0.32	≥95	≥1.7	$1 \cdot 10^{-5}$
THF	22.6	22	1.62	≥95	≥1.7	$1 \cdot 10^{-5}$

Figure S1 – Figure S12 show the thermal back-isomerization of metastable *Z*-**PBFT**. The thermal decay was monitored by either NMR or UV/Vis spectroscopy in the dark at the given temperature.

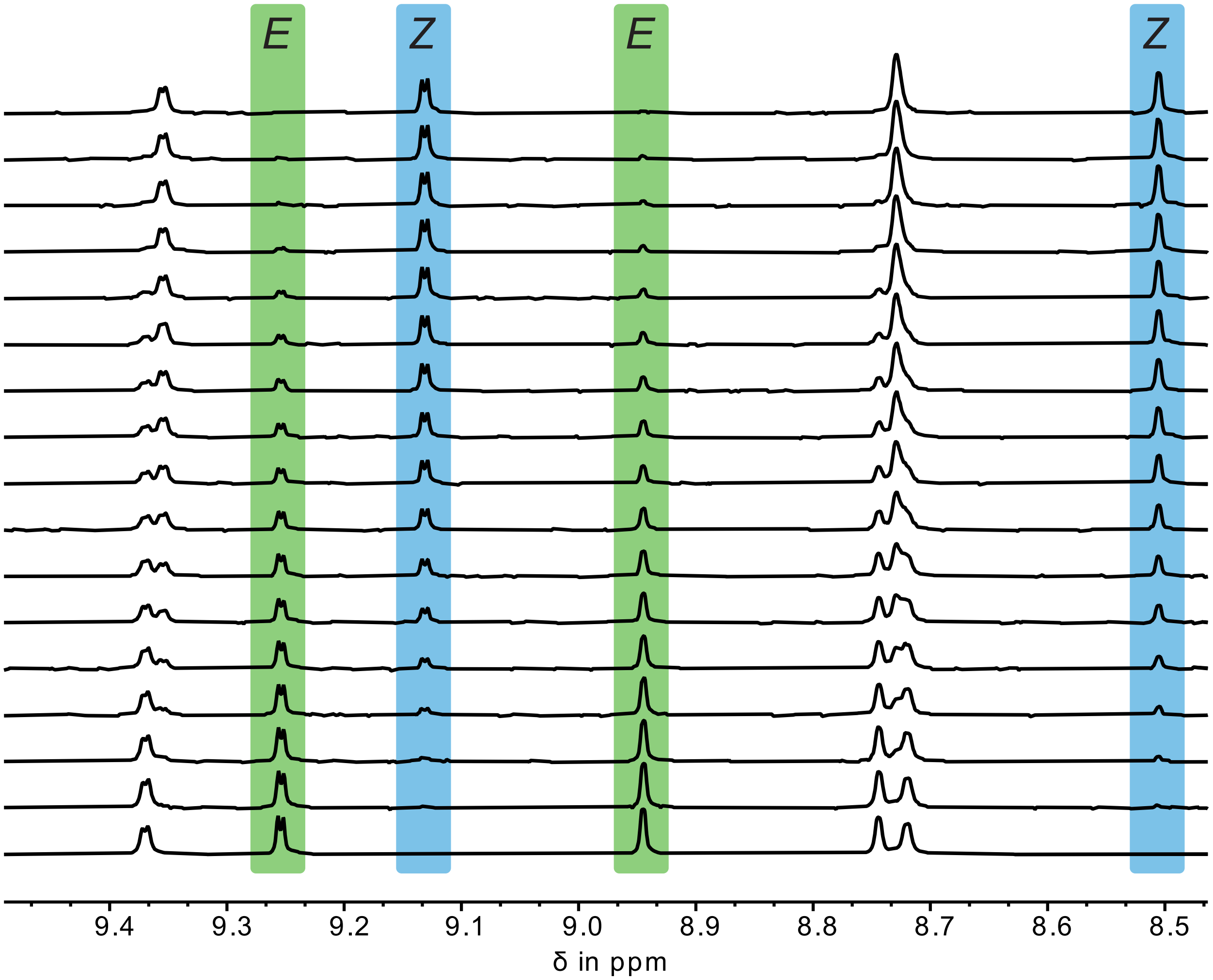

Figure S1 Thermal isomerization of *Z*-**PBFT** to *E*-**PBFT** at 15 °C over 19 h followed by 400 MHz ^{1}H NMR spectroscopy in benzene-d_6 in the dark.

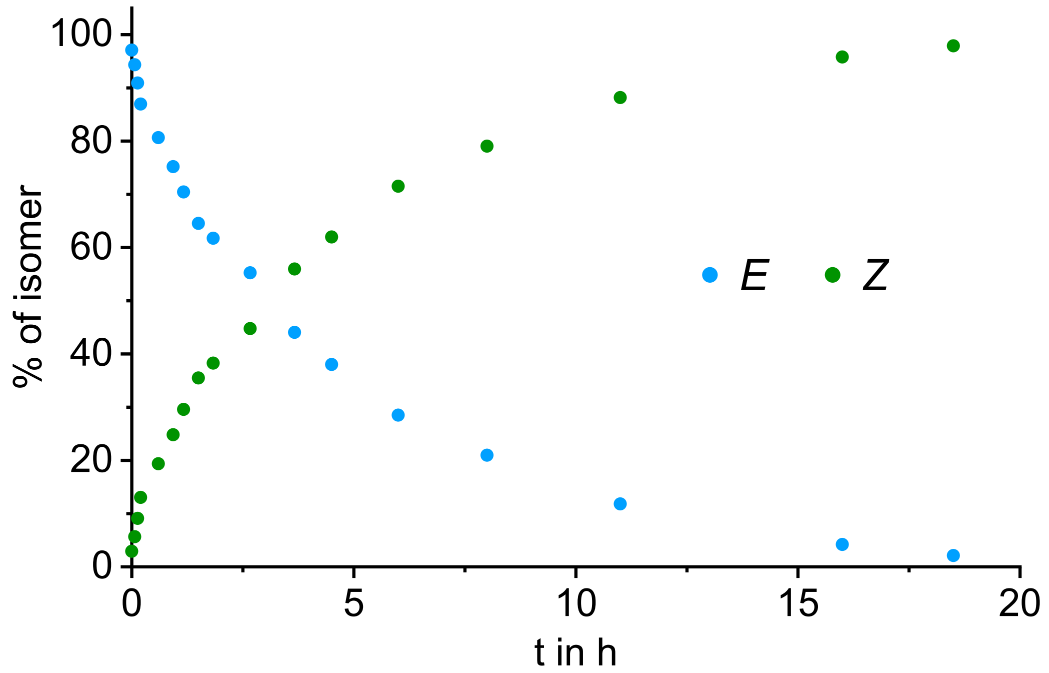

Figure S2 Thermal isomerization of *Z*-**PBFT** to *E*-**PBFT** in benzene-d_6 at 15 °C in the dark followed by ^{1}H NMR spectroscopy. Isomeric ratios are plotted versus time.

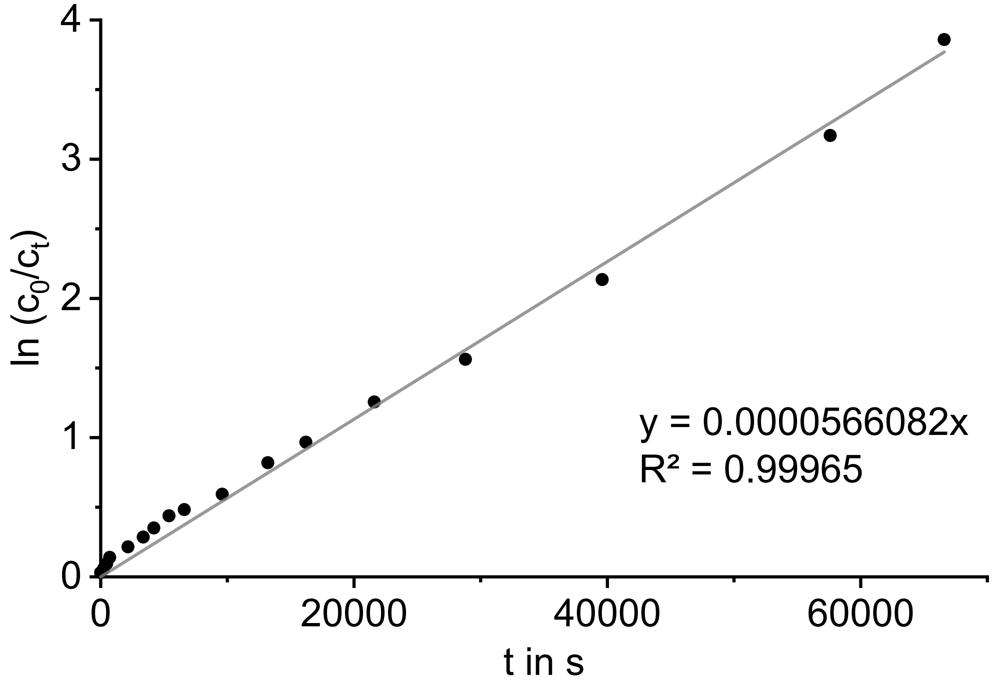

Figure S3 Kinetic analysis of decreasing *Z*-**PBFT** isomer in benzene-d_6 followed by ^{1}H NMR spectroscopy at 15 °C in the dark.

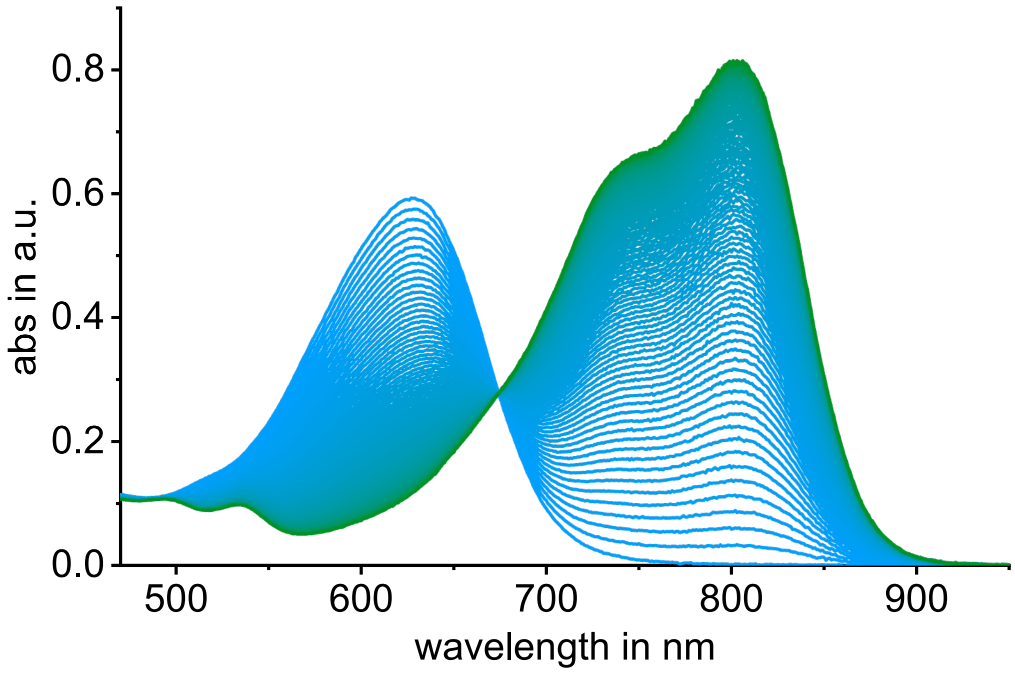

Figure S4 Thermal isomerization of PBFT *Z*-**PBFT** to stable *E*-**PBFT** followed by UV/Vis spectroscopy. Spectra were taken in 5-minute intervals in benzene solution at 22 °C in the dark.

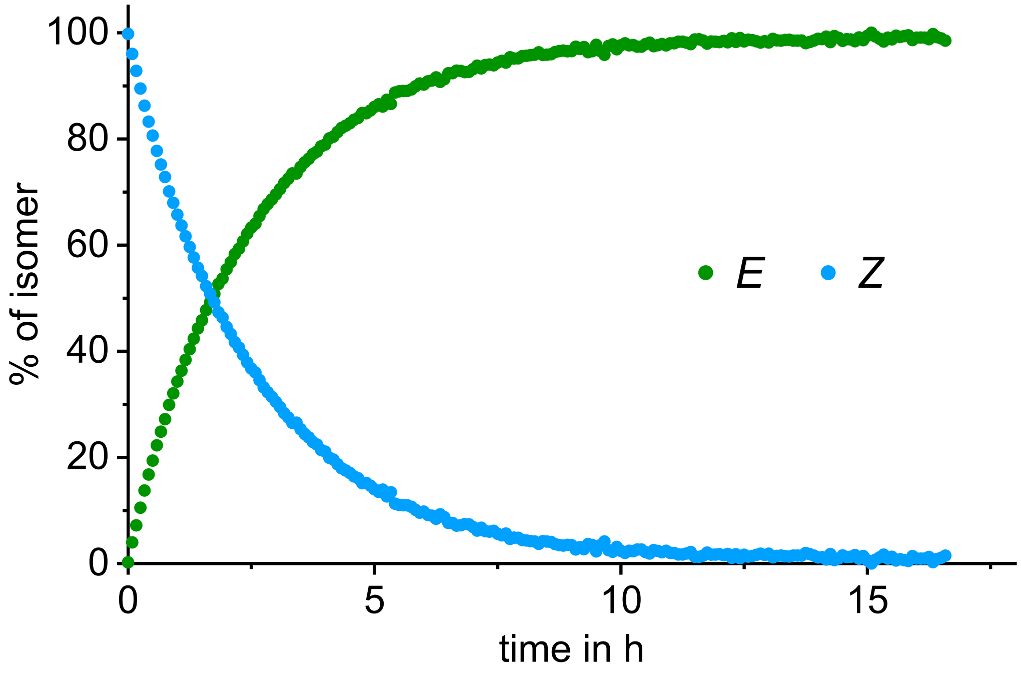

Figure S5 Thermal isomerization of PBFT *Z*-**PBFT** to stable *E*-**PBFT** followed by UV/Vis spectroscopy. Changing isomeric ratios are plotted during the thermal conversion of metastable *Z*-**PBFT** to stable *E*-**PBFT** in benzene solution at 22 °C in the dark.

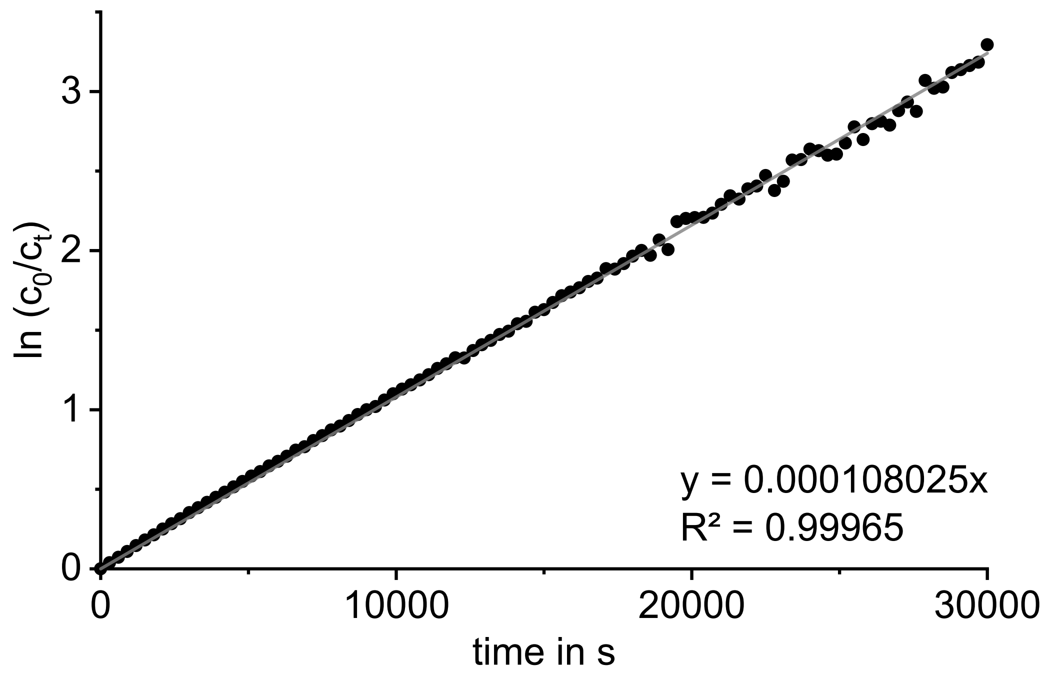

Figure S6 Kinetic analysis of decreasing *Z*-**PBFT** isomer followed by UV/Vis spectroscopy in benzene solution at 22 °C in the dark.

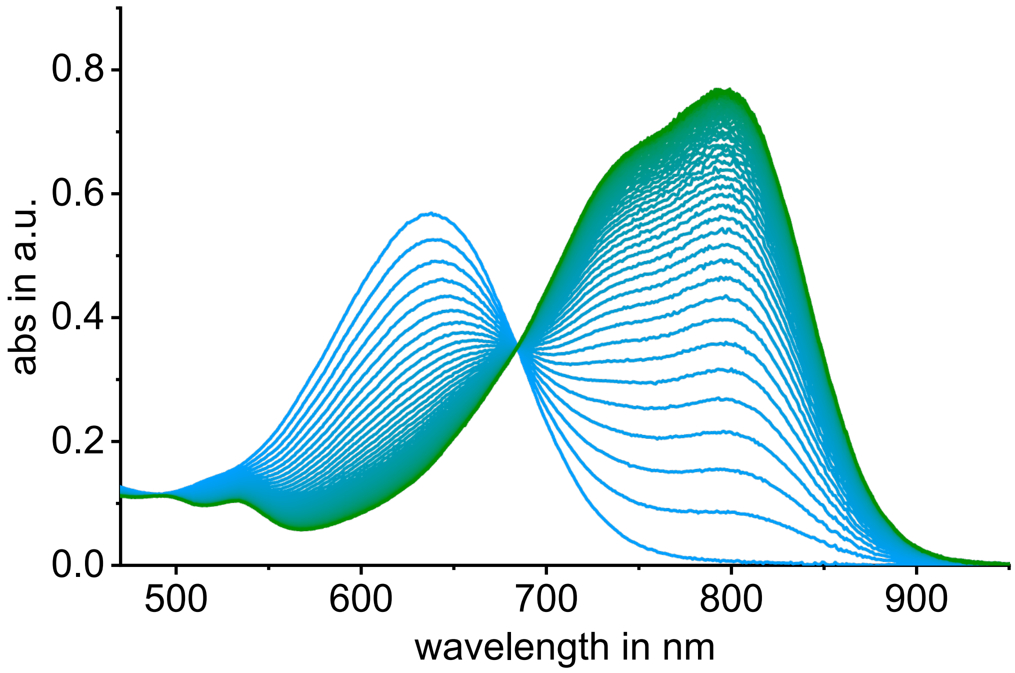

Figure S7 Thermal isomerization of PBFT *Z*-**PBFT** to stable *E*-**PBFT** followed by UV/Vis spectroscopy. Spectra were taken in 5-minute intervals in CH_2Cl_2 solution at 22 °C in the dark.

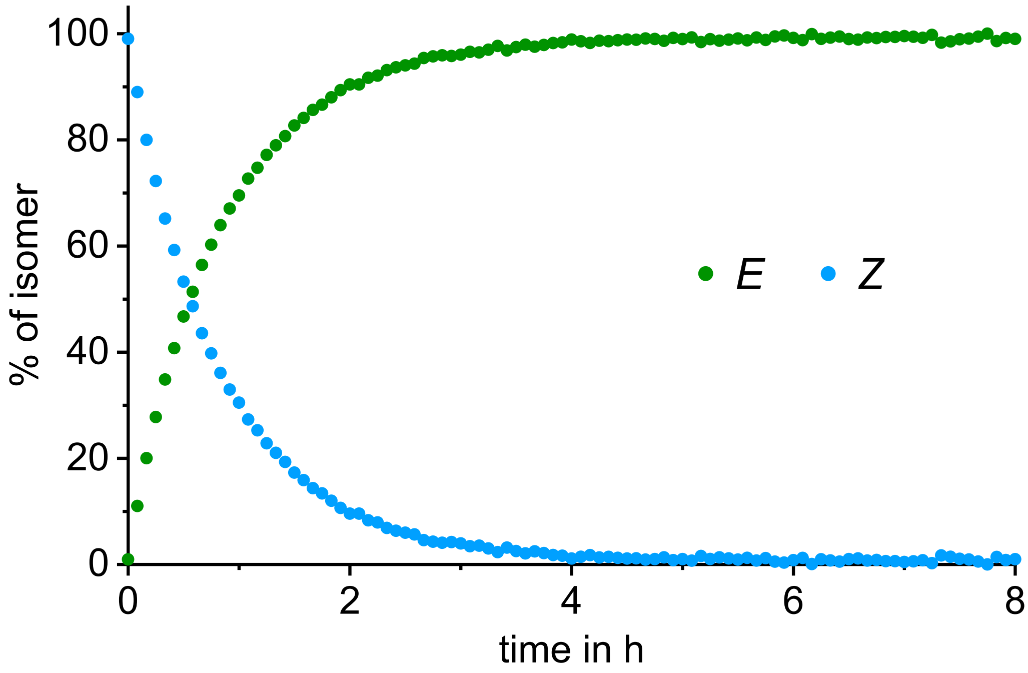

Figure S8 Thermal isomerization of PBFT *Z*-**PBFT** to stable *E*-**PBFT** followed by UV/Vis spectroscopy. Changing isomeric ratios are plotted during the thermal conversion of metastable *Z*-**PBFT** to stable *E*-**PBFT** in CH_2Cl_2 solution at 22 °C in the dark.

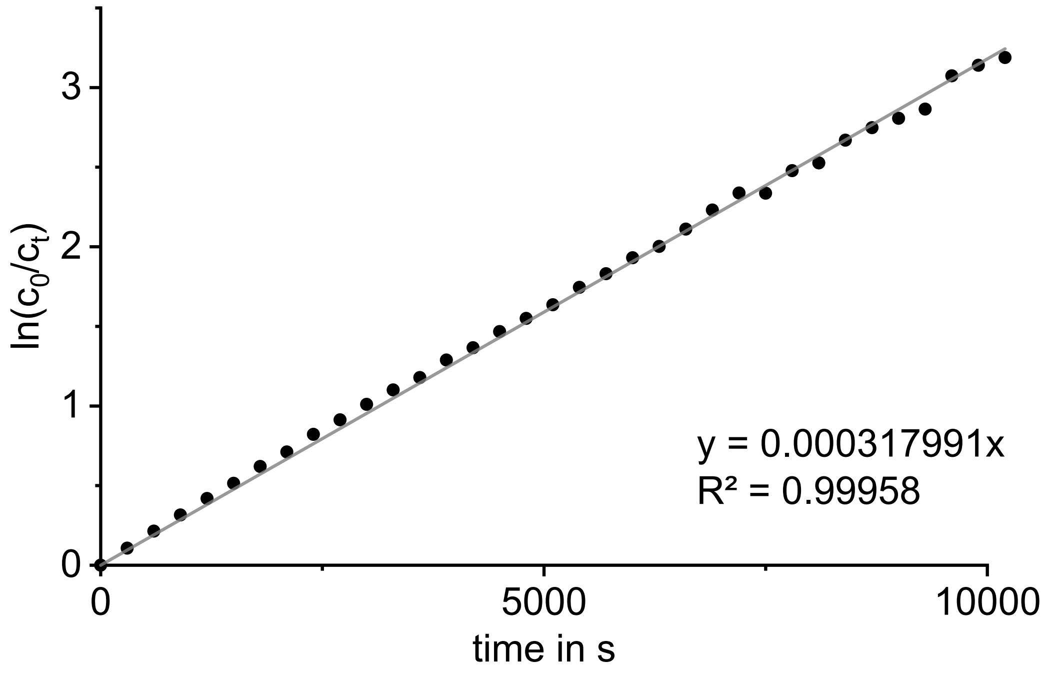

Figure S9 Kinetic analysis of decreasing *Z*-**PBFT** isomer followed by UV/Vis spectroscopy in CH_2Cl_2 solution at 22 °C in the dark.

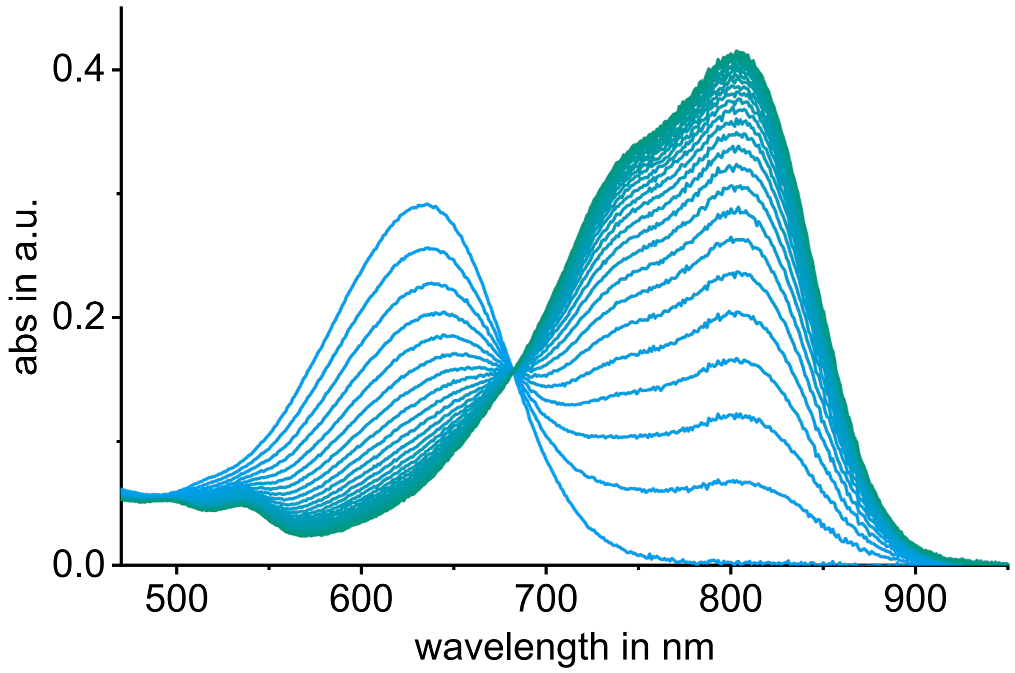

Figure S10 Thermal isomerization of PBFT *Z*-**PBFT** to stable *E*-**PBFT** followed by UV/Vis spectroscopy. Spectra were taken in 5-minute intervals in pyridine solution at 22 °C in the dark.

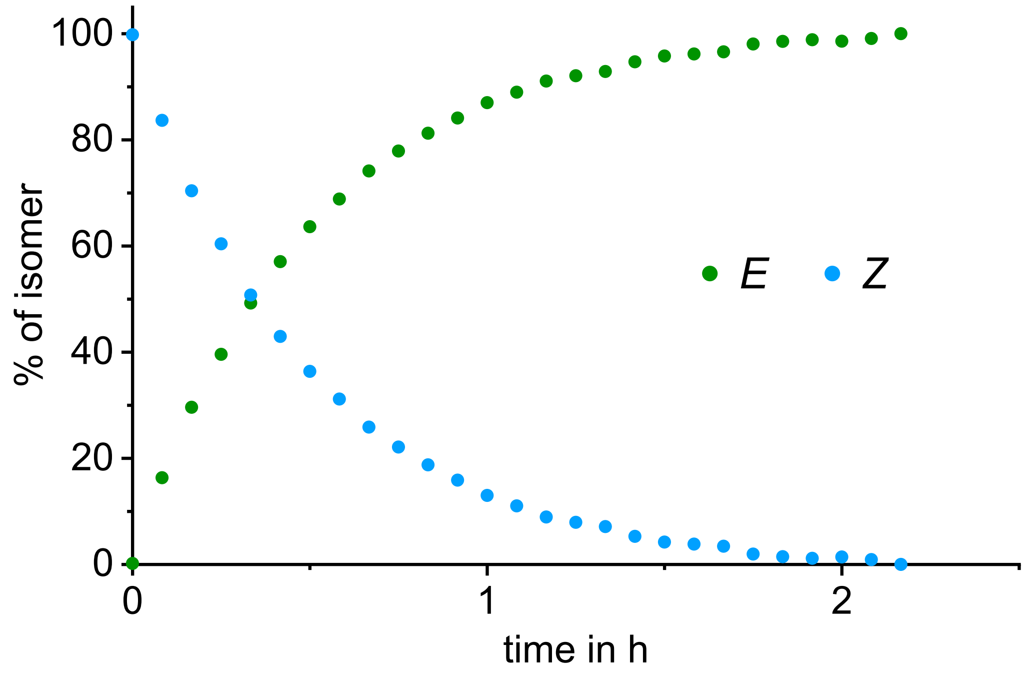

Figure S11 Thermal isomerization of PBFT *Z*-**PBFT** to stable *E*-**PBFT** followed by UV/Vis spectroscopy. Changing isomeric ratios are plotted during the thermal conversion of metastable *Z*-**PBFT** to stable *E*-**PBFT** in pyridine solution at 22 °C in the dark.

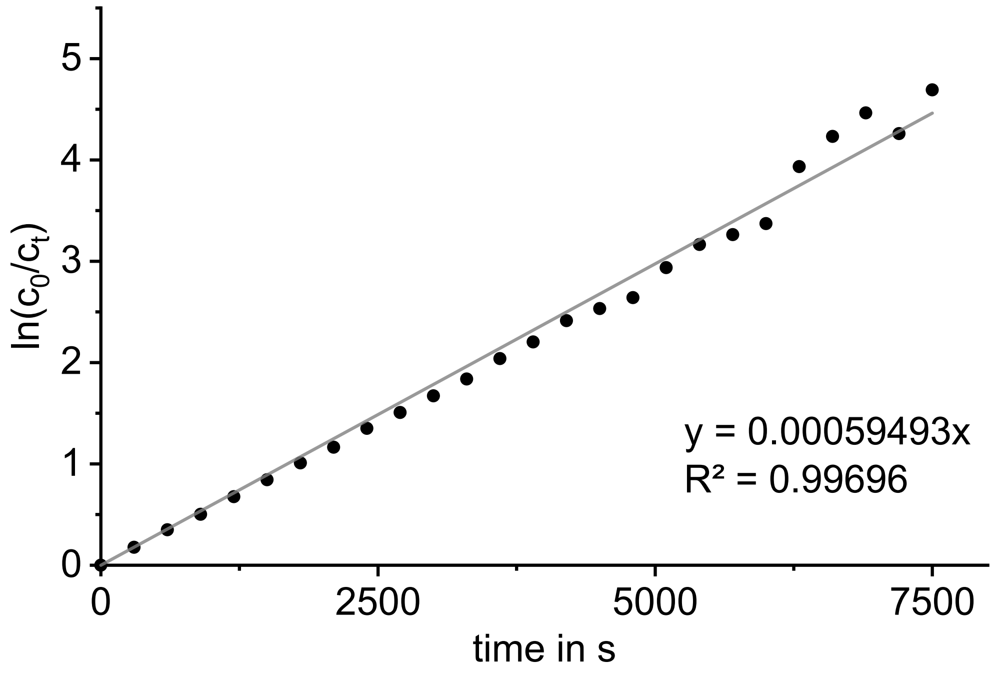

Figure S12 Kinetic analysis of decreasing *Z*-**PBFT** isomer followed by UV/Vis spectroscopy in pyridine solution at 22 °C in the dark.

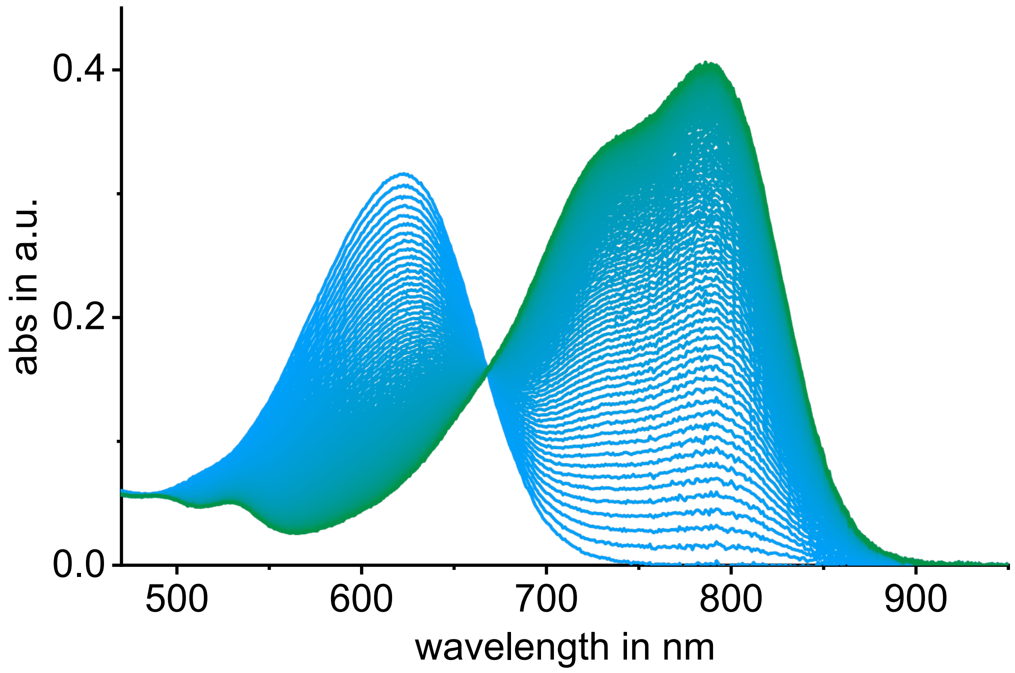

Figure S13 Thermal isomerization of PBFT *Z*-**PBFT** to stable *E*-**PBFT** followed by UV/Vis spectroscopy. Spectra were taken in 5-minute intervals in THF solution at 22 °C in the dark.

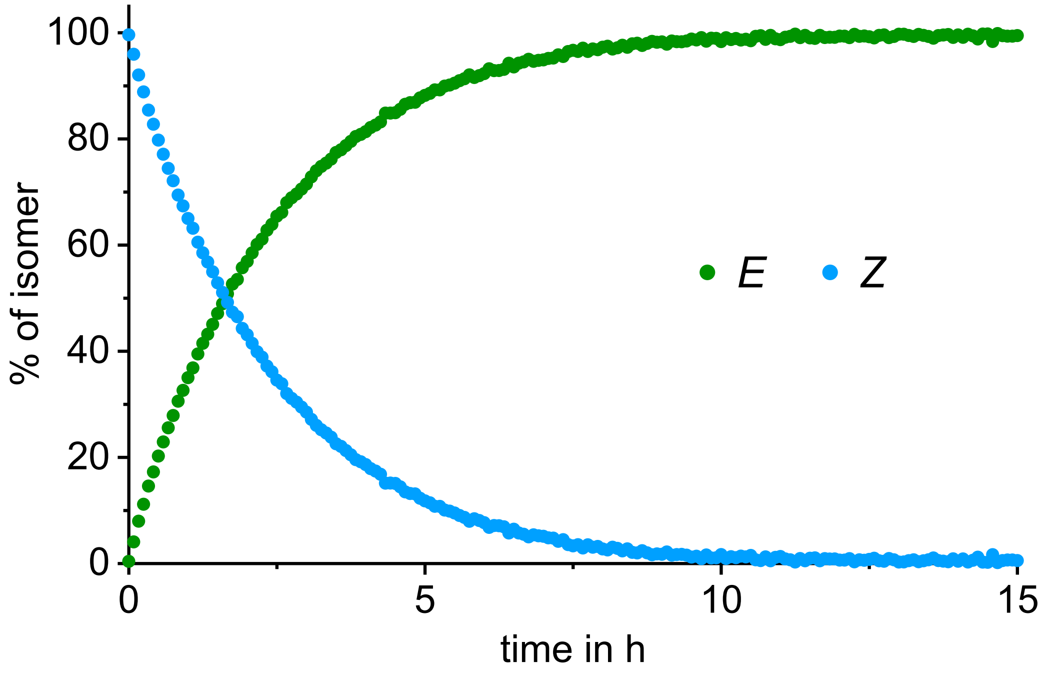

Figure S14 Thermal isomerization of PBFT *Z*-**PBFT** to stable *E*-**PBFT** followed by UV/Vis spectroscopy. Changing isomeric ratios are plotted during the thermal conversion of metastable *Z*-**PBFT** to stable *E*-**PBFT** in THF solution at 22 °C in the dark.

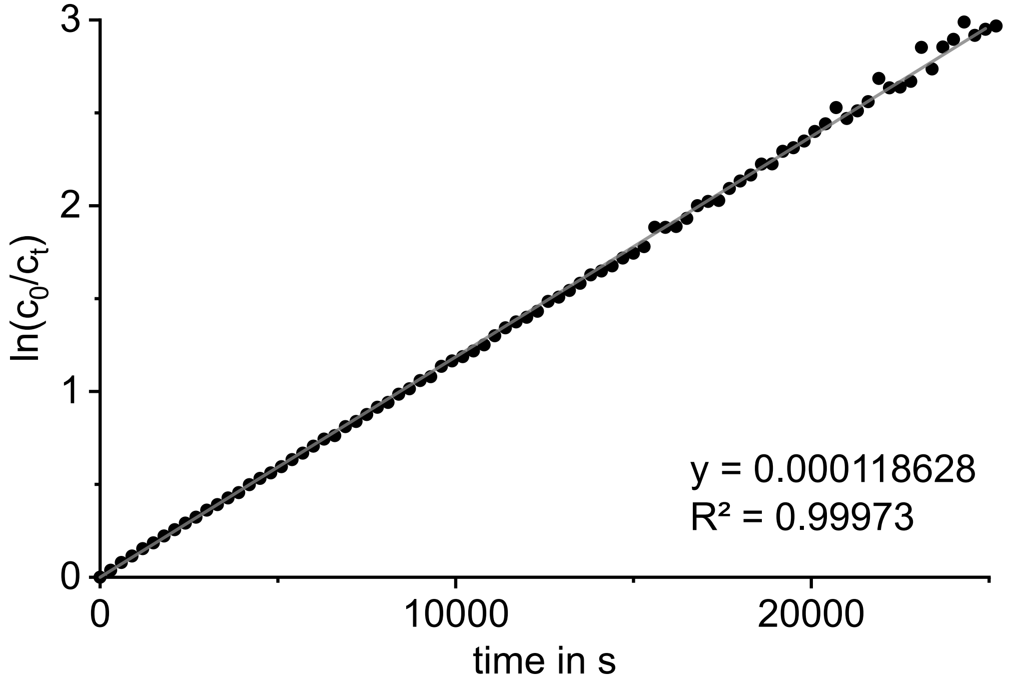

Figure S15 Kinetic analysis of decreasing *Z*-**PBFT** isomer followed by UV/Vis spectroscopy in THF solution at 22 °C in the dark.

Photophysical and photochemical properties of PBFT

Isomer composition in the pss monitored by ^{1}H NMR spectroscopy

To evaluate the isomer composition in the photostationary state (pss), a sample of **PBFT** in deuterated benzene (c = ~4 • 10^{-3} mol L^{-1}) was prepared in an NMR tube. The sample was irradiated outside the NMR spectrometer under cooling to 0 °C in an ice-cooling bath with different wavelengths of light until no further isomeric change was detected and the pss was reached. The isomeric ratio after each irradiation was determined by ^{1}H NMR spectroscopy. The measurement was carried out immediately after irradiating the cooled sample to prevent thermal isomerization.

Table S2 Isomeric ratios of **PBFT** in the pss at different wavelengths of irradiation as obtained from integrated ^{1}H NMR spectra. Irradiation was conducted outside the NMR spectrometer at 0 °C. The samples were measured in benzene-d_6 at 23 °C.

λ E → Z	***E : Z***	***λ Z → E***	***E : Z***
905 nm	0 : 100	680 nm	92:8
880 nm	0 : 100	650 nm	100:0
850 nm	0 : 100	625 nm	100:0
830 nm	0 : 100	595 nm	99:1
810 nm	0 : 100	565 nm	100:0
780 nm	0 : 100		
730 nm	49 : 51		

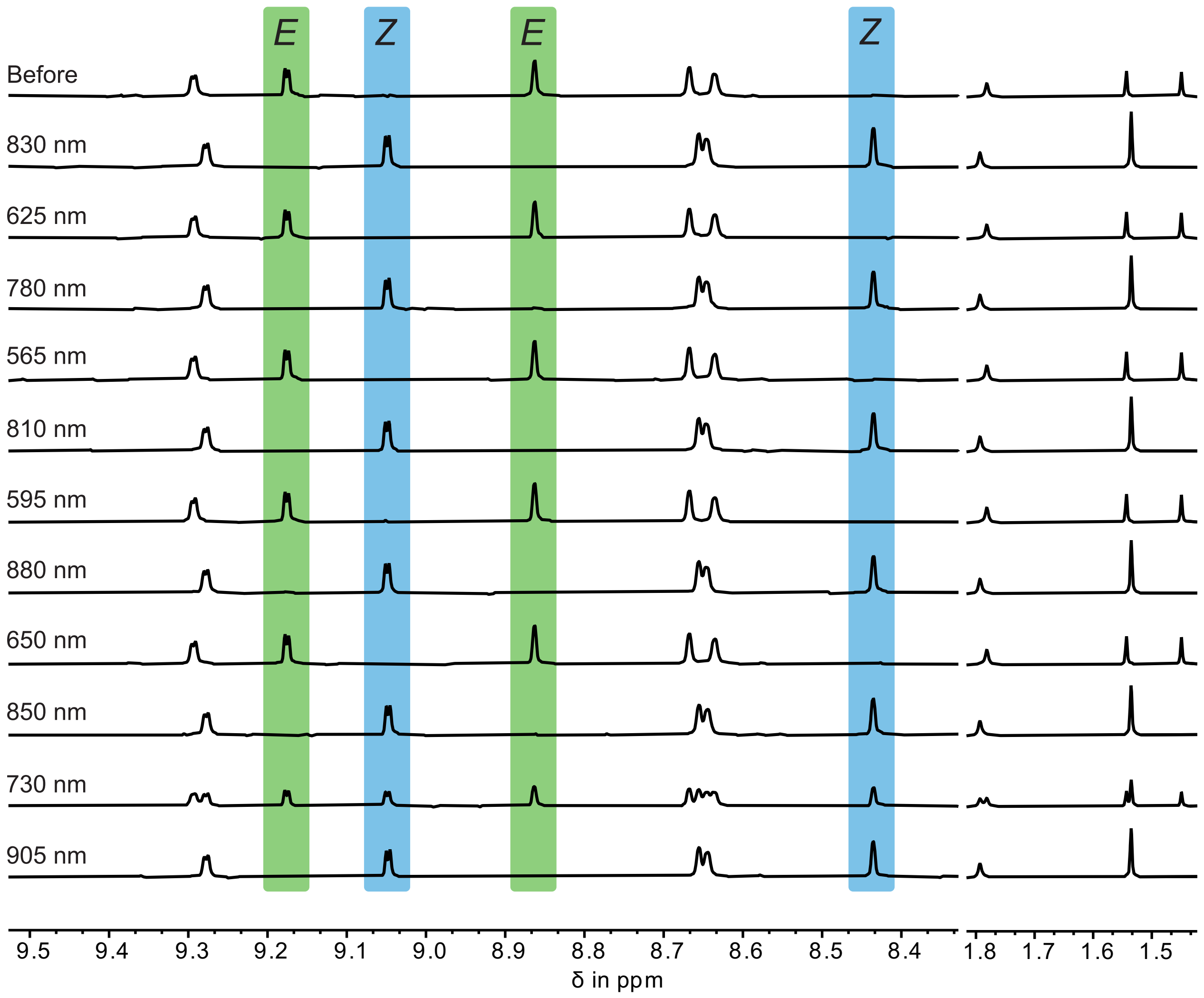

Figure S16 ^{1}H NMR spectra (400 MHz, benzene-d_6, 23 °C inside the spectrometer) of *E*-**PBFT** and *Z*-**PBFT** enriched solutions. Each irradiation was carried out at 0 °C until the pss was reached and no further change in the isomeric ratio could be observed.

Isomer composition in the pss monitored by UV/Vis spectroscopy

Using the molar absorption coefficients obtained at λ_{max} for both **PBFT** isomers *E* and *Z*, the isomer composition in the photostationary state at different wavelengths of irradiation could be calculated. A sample of **PBFT** was dissolved in benzene, CH_2Cl_2, pyridine, or THF (c = ~4 • 10^{-3} mol L^{-1}) and irradiated with LEDs of different wavelengths to either enrich the *Z* or *E* isomer. Each irradiation was carried out until no further change was detected and the corresponding UV/Vis spectrum was used for obtaining the isomer ratio in the pss.

Table S3 Isomeric mixtures of **PBFT** obtained after reaching the pss upon irradiation with different LEDs. The ratios were obtained at 22 °C using UV/Vis spectroscopy and measured in benzene, CH_2Cl_2, pyridine, and THF (c = ~4 • 10^{-5} mol L^{-1}).

	λ E → Z	***E : Z***	***λ Z → E***	***E : Z***
	905 nm	1:99	650 nm	97:3
	880 nm	0:100	625 nm	100:0
	850 nm	0:100	595 nm	100:0
benzene	830 nm	0:100	565 nm	98:2
	810 nm	0:100		
	780 nm	2:98		
	730 nm	38:62		
	905 nm	3:97	650 nm	95:5
	880 nm	0:100	625 nm	97:3
	850 nm	0:100	595 nm	97:3
CH_2Cl_2	830 nm	0:100	565 nm	96:4
	810 nm	3:97		
	780 nm	7:93		
	905 nm	2:98	650 nm	96:4
	880 nm	0:100	625 nm	96:4
	850 nm	0:100	595 nm	96:4

pyridine	830 nm	0:100	565 nm	94:6
	810 nm	2:98		
	780 nm	4:96		
THF	905 nm	7:93	650 nm	95:5
	880 nm	0:100	625 nm	100:0
	850 nm	0:100	595 nm	99:1
	830 nm	0:100	565 nm	99:1
	810 nm	0:100		
	780 nm	99:1		

Molar absorption coefficients of PBFT

The molar absorption coefficients ε of both **PBFT** isomers were obtained via UV/Vis spectroscopy using the *Lambert-Beer* law. Cuvettes with solutions of known concentrations ($c = 1 \cdot 10^{-5}$ mol L^{-1}) of **PBFT** were prepared with four different solvents (benzene, CH_2Cl_2, pyridine, THF) and spectra of the pure *E* isomers were measured. Subsequently, the *Z* isomer was enriched quantitatively using proper wavelengths for irradiation and spectra of the pure *Z* isomers were obtained.

Table S4 Comparison of molar absorption coefficients ε of both **PBFT** isomers *E* and *Z* and maximum wavelengths λ_{max} of the most red-shifted absorption band in benzene, CH_2Cl_2, pyridine, and THF ($c = 1 \cdot 10^{-5}$ mol L^{-1}) measured at 22 °C. The absolute difference between both maxima $\Delta\lambda_{max}$ is also given for each solvent.

	***E* isomer**		***Z* isomer**		
	λ_{max} [nm]	ε [L mol^{-1} cm^{-1}]	λ_{max} [nm]	ε [L mol^{-1} cm^{-1}]	$\Delta\lambda_{max}$ nm
benzene	802	43000	627	31200	175
pyridine	804	41500	634	29300	170
CH2Cl2	795	42300	638	31200	157
THF	788	40700	622	31900	157

Spectra of the determined molar absorption coefficients are shown in Figure S17-S20.

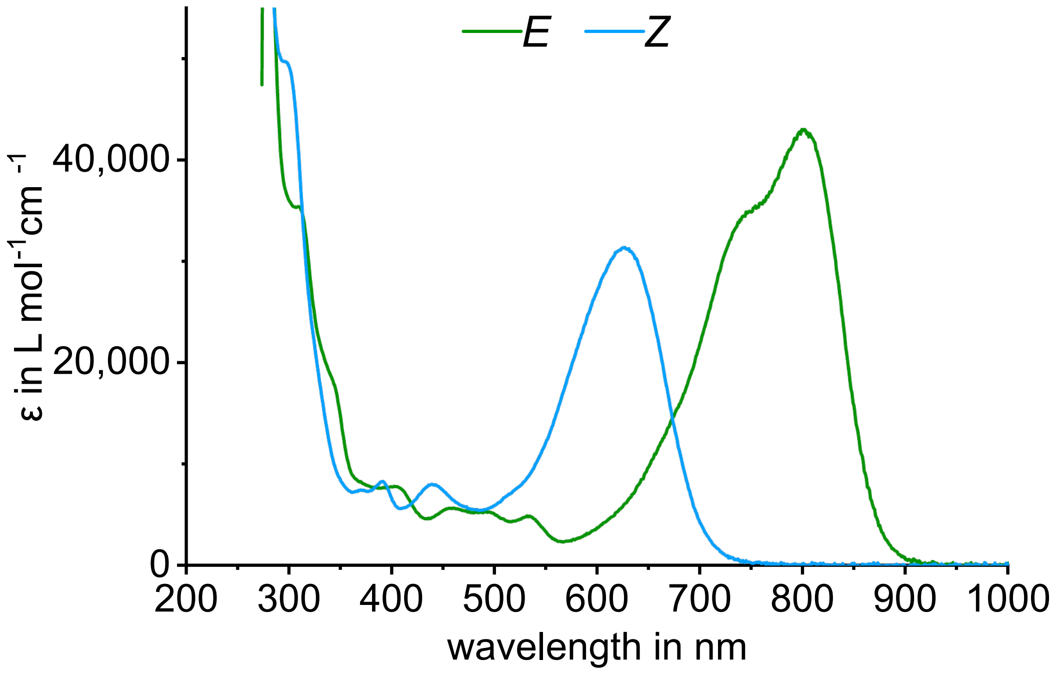

Figure S17 Molar absorption coefficients *ε* of *E*-**PBFT** (green) and *Z*-**PBFT** (blue) in benzene solution at 22 °C.

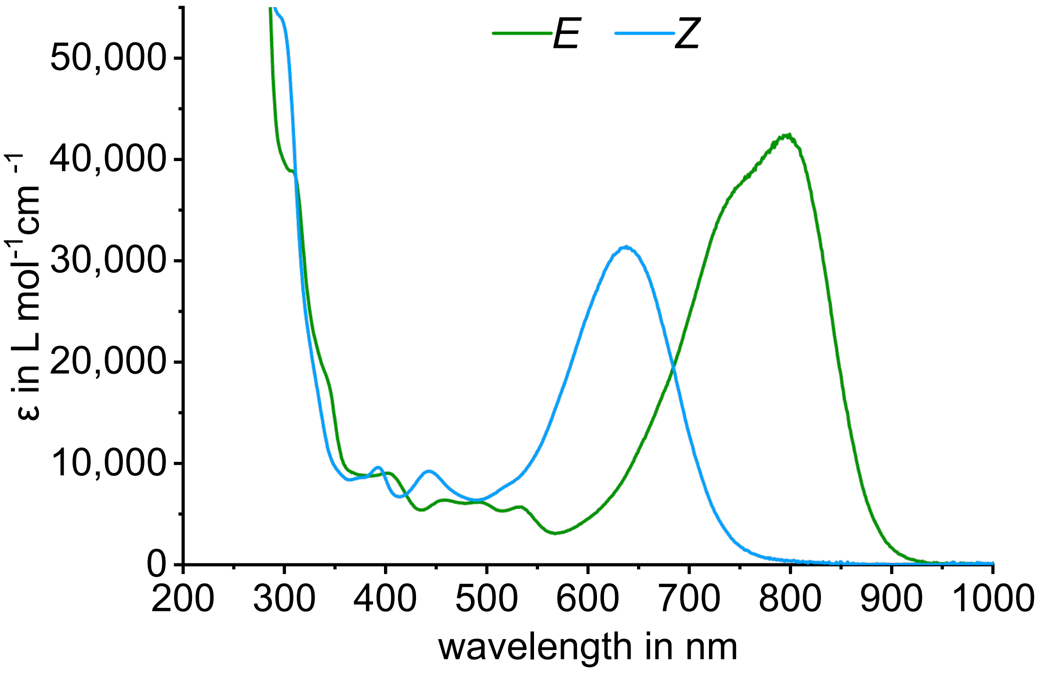

Figure S18 Molar absorption coefficients *ε* of *E*-**PBFT** (green) and *Z*-**PBFT** (blue) in CH_2Cl_2 solution at 22 °C.

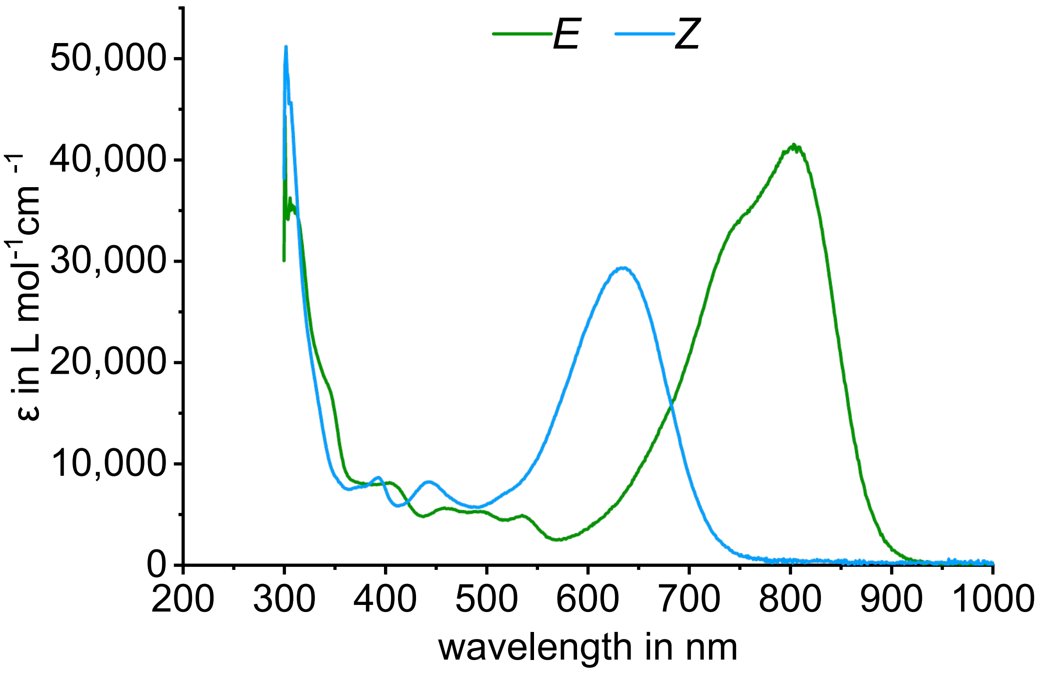

Figure S19 Molar absorption coefficients *ε* of *E*-**PBFT** (green)and *Z*-**PBFT** (blue) in pyridine solution at 22 °C.

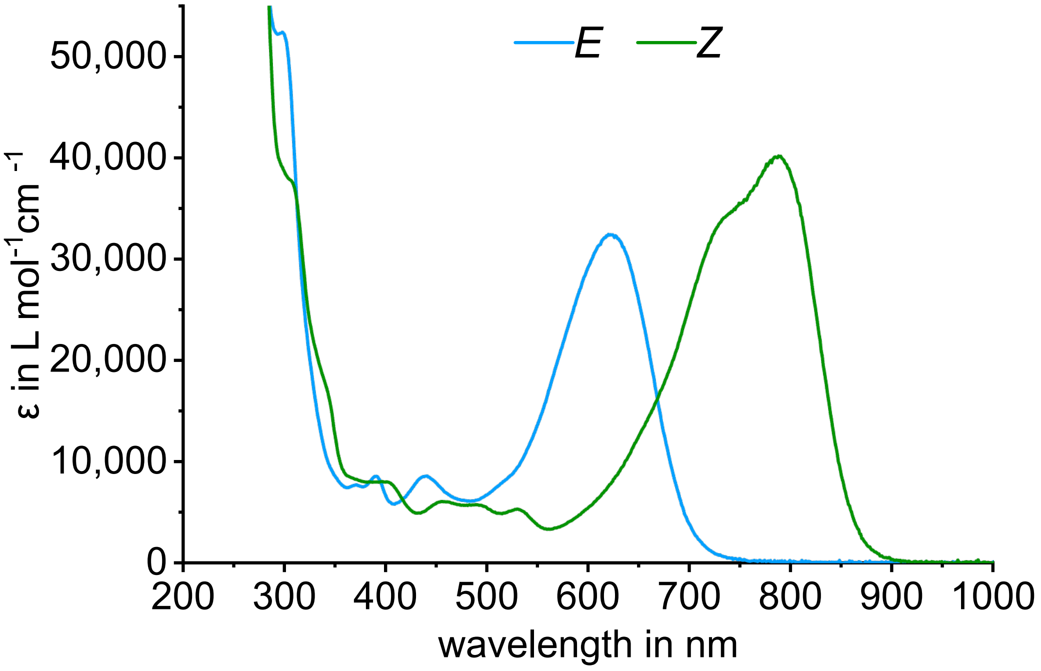

Figure S20 Molar absorption coefficients *ε* of *E*-**PBFT** (green) and *Z*-**PBFT** (blue) in THF solution at 22 °C.

Quantum Yield Determination of PBFT

Quantum yields were determined using a setup developed by the group of *E. Riedle*.[S5] The photochemical quantum yields $\Phi_{E\rightarrow Z}$ for the *E*/*Z* photoisomerization and $\Phi_{Z\rightarrow E}$ for the *Z*/*E* photoisomerization of **PBFT** were determined using the equation 5:

$$\Phi = \frac{N\ (isomerized\ molecules)}{N\ (absorbed\ photons)} \qquad \text{eq. 5}$$

For the measurements, a cuvette was prepared with a solution of **PBFT** in benzene (2.25 mL) and irradiated at 720 nm for specific time intervals calculated by the setup's software. After each irradiation step, a UV/Vis spectrum was recorded. The results for both isomerization processes are shown in Table S5.

Table S5 Photochemical quantum yields for both photoisomerizations *E* to *Z* and *Z* to *E* of **PBFT** starting from both pure isomers respectively.

solvent	$\Phi_{E\rightarrow Z}$	$\Phi_{Z\rightarrow E}$	λ_{LED}
benzene (starting from E)	4%	42%	720 nm
benzene (starting from Z)	5%	48%	720 nm

Starting from pure *E* or *Z* isomer, the sample (c = $2\cdot10^{-5}$ mol L^{-1}) was irradiated with a 720 nm (1.86 V, 0.291 A) LED for distinct time intervals. After each interval, a UV/Vis spectrum was recorded at 23 °C. The overall irradiation time was about 680 s for the *E*/*Z* isomerization and about 570 s for the *Z*/*E* isomerization process. The recorded UV/Vis spectra as well as the changes in concentration over time are shown in Figure S21-S24.

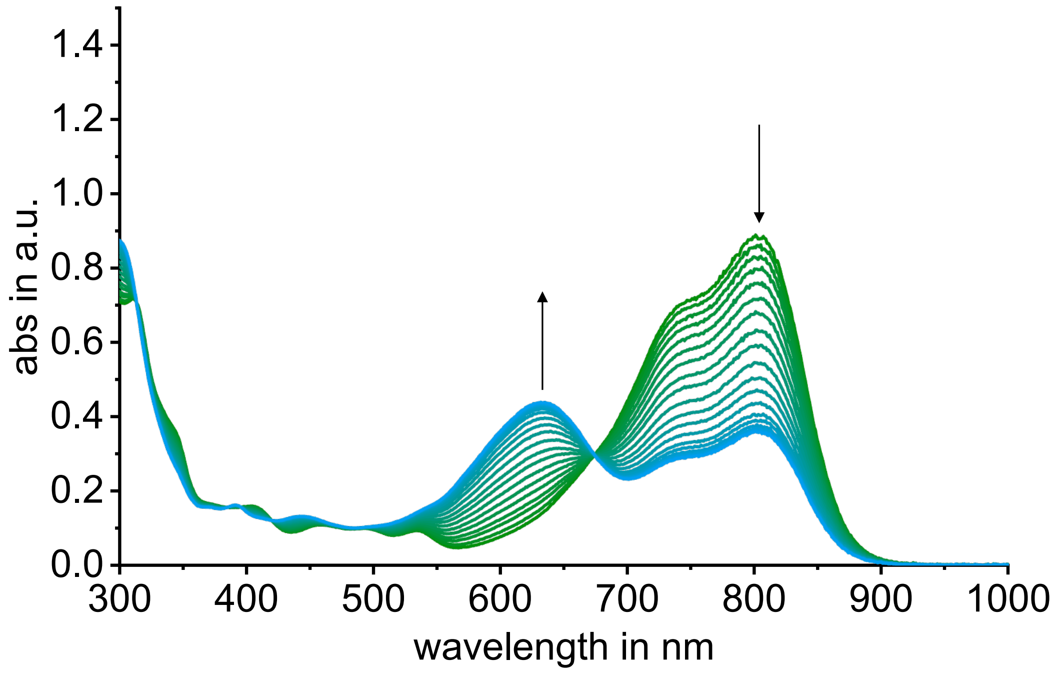

Figure S21 UV/Vis spectra of **PBFT** recorded after each irradiation interval using a 720 nm LED starting from pure *E* isomer.

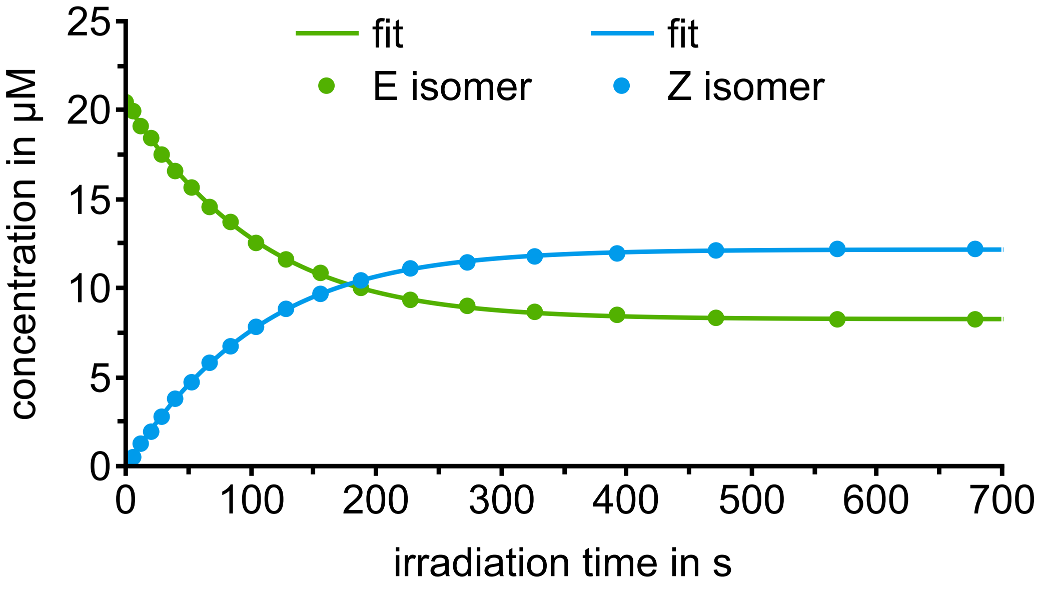

Figure S22 Chancing concentration of *E* and *Z* isomers of **PBFT** over time during the stepwise irradiation with 720 nm light.

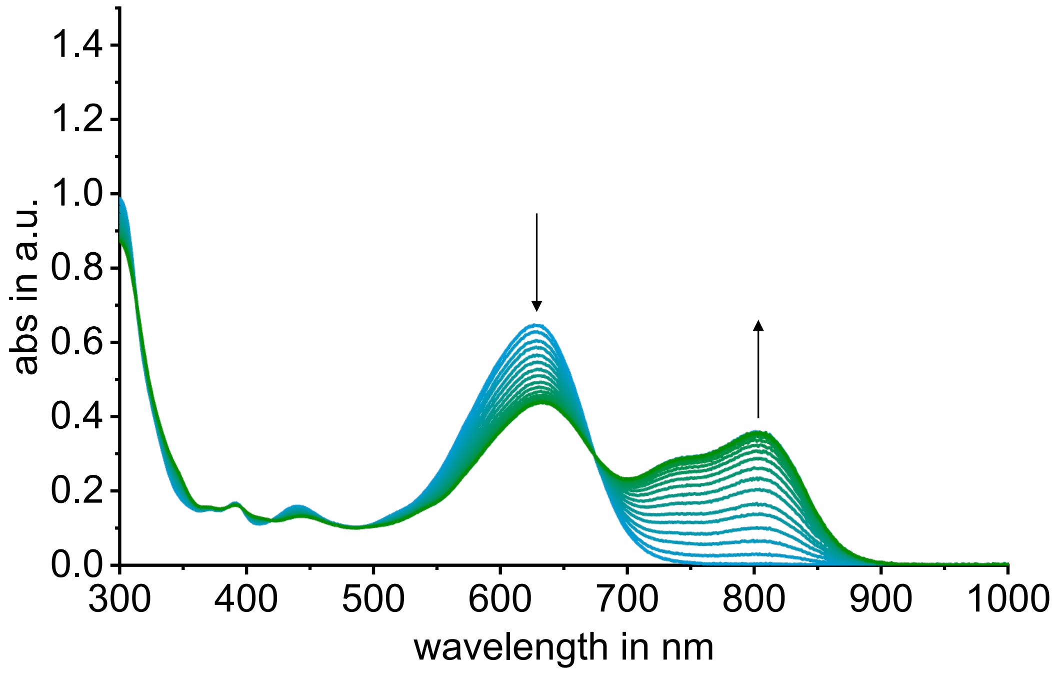

Figure S23 UV/Vis spectra of **PBFT** recorded after each irradiation interval using a 720 nm LED starting from pure *Z* isomer.

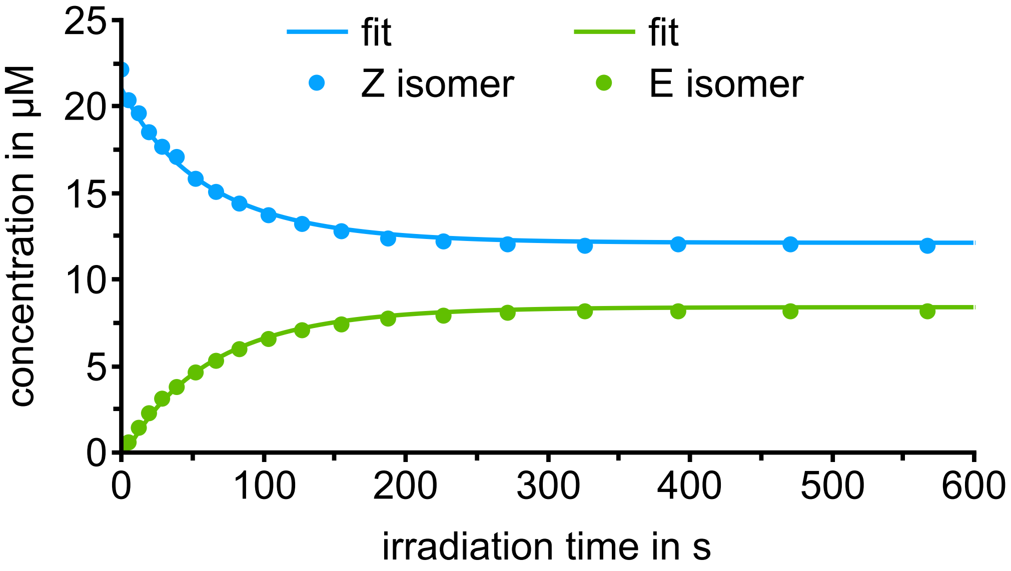

Figure S24 Changing concentration of *E* and *Z* isomers of **PBFT** over time during the stepwise irradiation with 720 nm light.

Photoisomerization of PBFT followed by UV/Vis spectroscopy

Figure S25 shows the absorption of **PBFT** after irradiation of *E*-**PBFT** with 850 nm light and *Z*-**PBFT** with 625 nm light for the given time intervals. A sample of pure *E*-**PBFT** ($2.1 \cdot 10^{-5}$ mol L^{-1}) was irradiated to enrich *Z*-**PBFT** followed by UV/Vis spectroscopy measurement. The *Z*-**PBFT** to *E*-**PBFT** photoisomerization was executed similarly with 625 nm light.

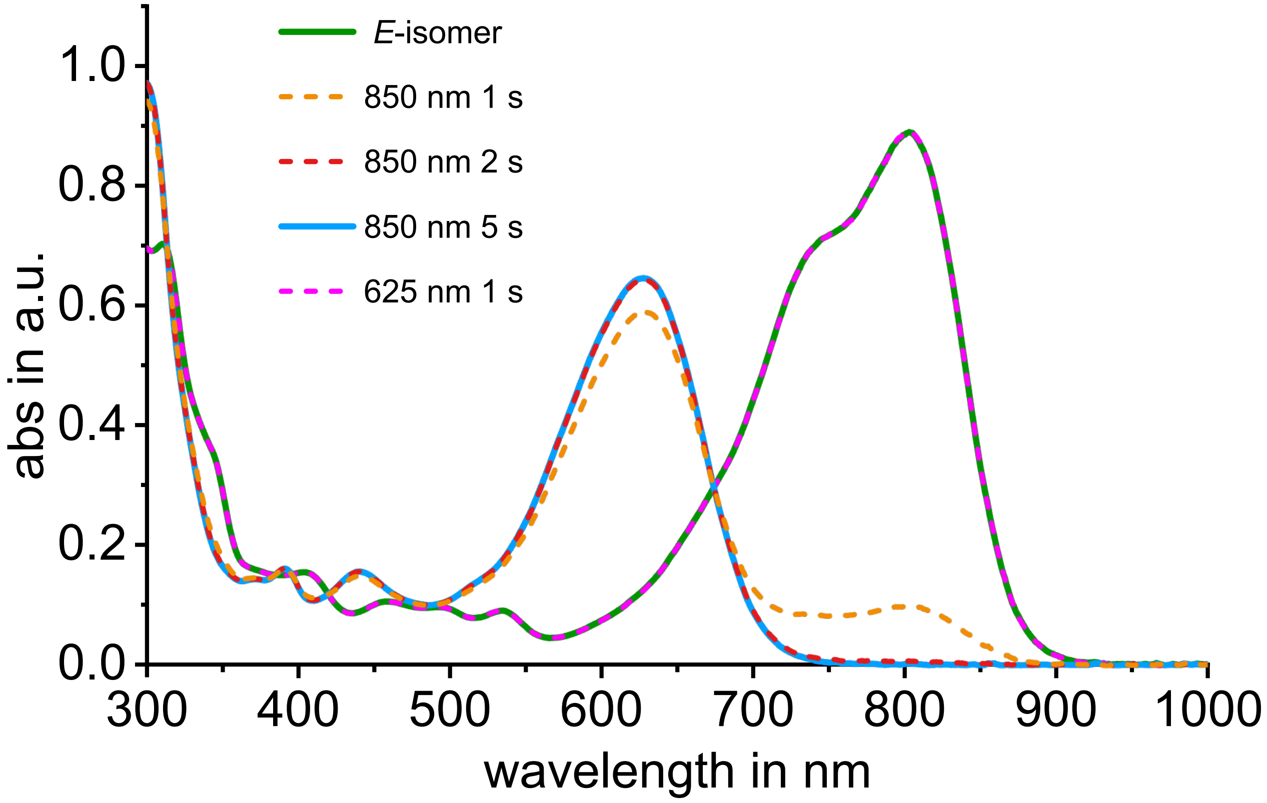

Figure S25 Absorption spectra of **PBFT** obtained after irradiation with different LEDs for the given time intervals at 22 °C. First, the pure *E* isomer was measured. The sample was then irradiated with 850 nm to enrich the *Z* isomer followed by UV/Vis spectroscopy. Likewise, the *E* isomer was enriched using 625 nm light.

Photostability of PBFT

Information about the photochemical stability of **PBFT** was obtained by subjecting it to fifteen photoswitching cycles. Four samples in UV/Vis concentration were prepared in four different spectroscopic solvents. Enriching the *Z* isomer was accomplished by irradiating the corresponding sample with 850 nm light. To accumulate the *E* isomer, the sample was irradiated with 625 nm light. Each irradiation was carried out until the pss was reached, followed by the recording of a UV/Vis spectrum. This sequence was repeated 15 times and the absorbance at a specific wavelength and the isosbestic point at each cycle were plotted and are shown in Figures S26-S29 below.

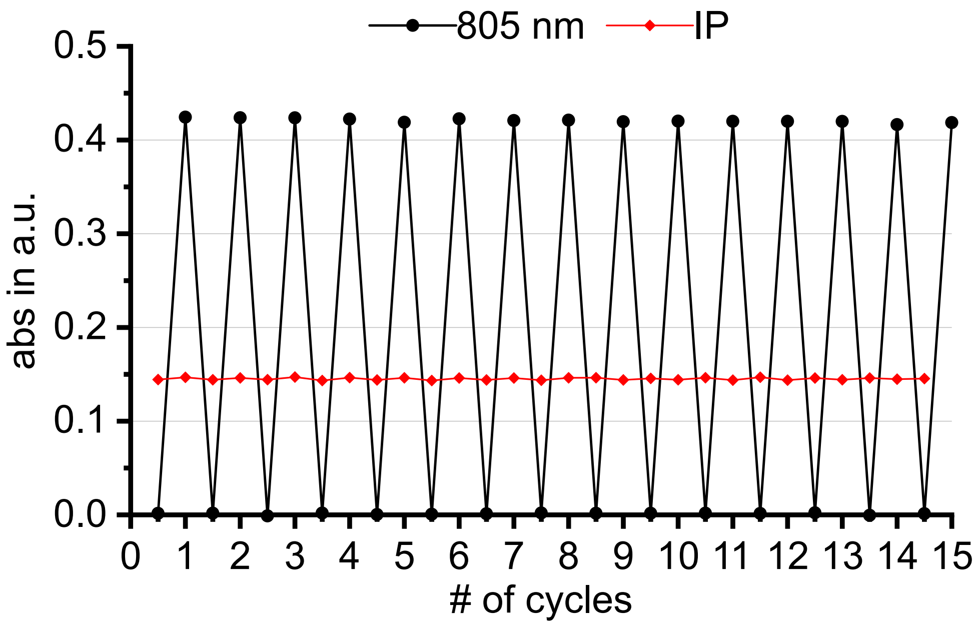

Figure S26 Absorption at 805 nm and the isosbestic point at 673 nm measured for each switching cycle of **PBFT** in benzene solution. Alternating irradiation with 850 nm and 625 nm light was conducted for each cycle until the respective pss was reached.

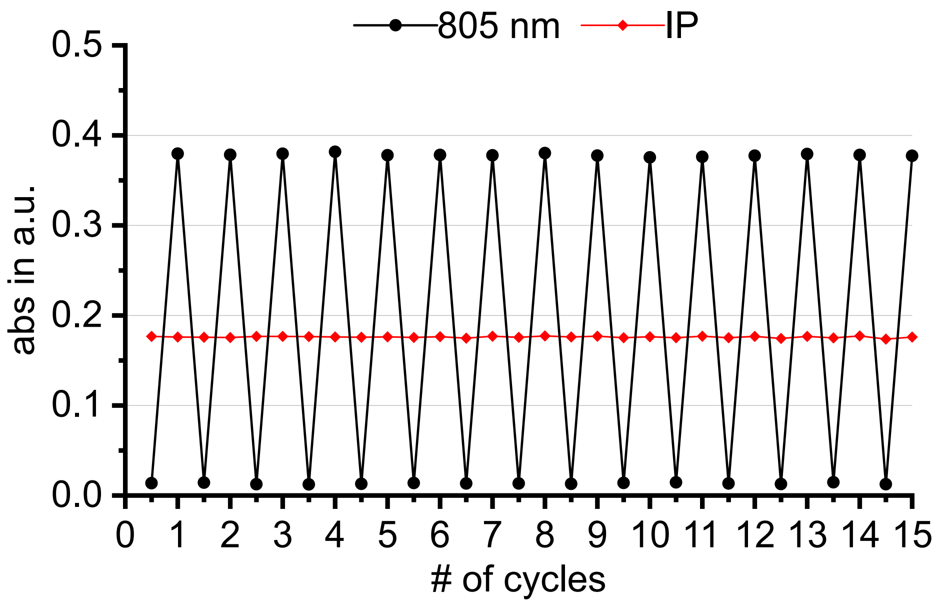

Figure S27 Absorption at 805 nm and the isosbestic point at 683 nm measured for each switching cycle of **PBFT** in CH_2Cl_2 solution. Alternating irradiation with 850 nm and 625 nm light was conducted for each cycle until the respective pss was reached.

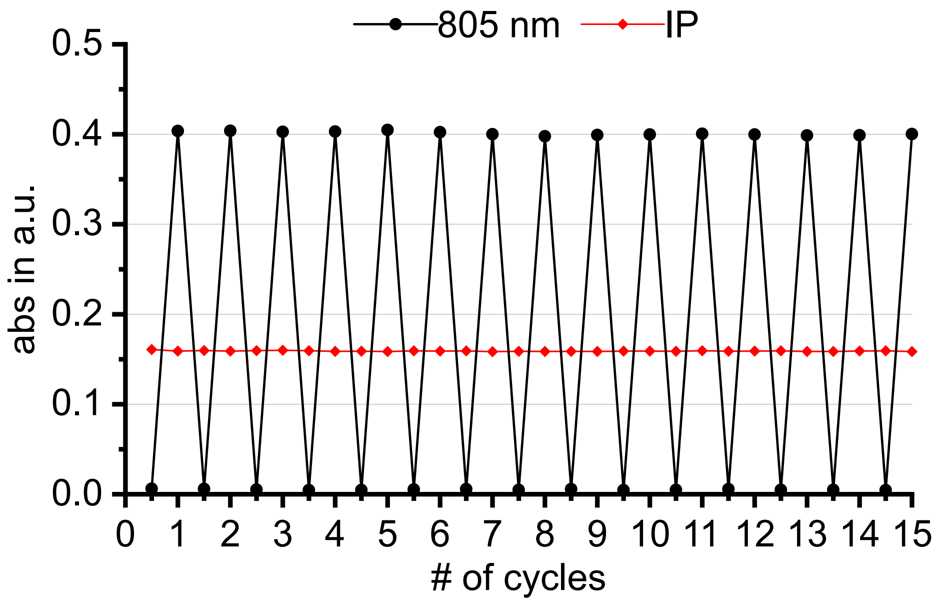

Figure S28 Absorption at 805 nm and the isosbestic point at 682 nm measured for each switching cycle of **PBFT** in pyridine solution. Alternating irradiation with 850 nm and 625 nm light was conducted for each cycle until the respective pss was reached.

.

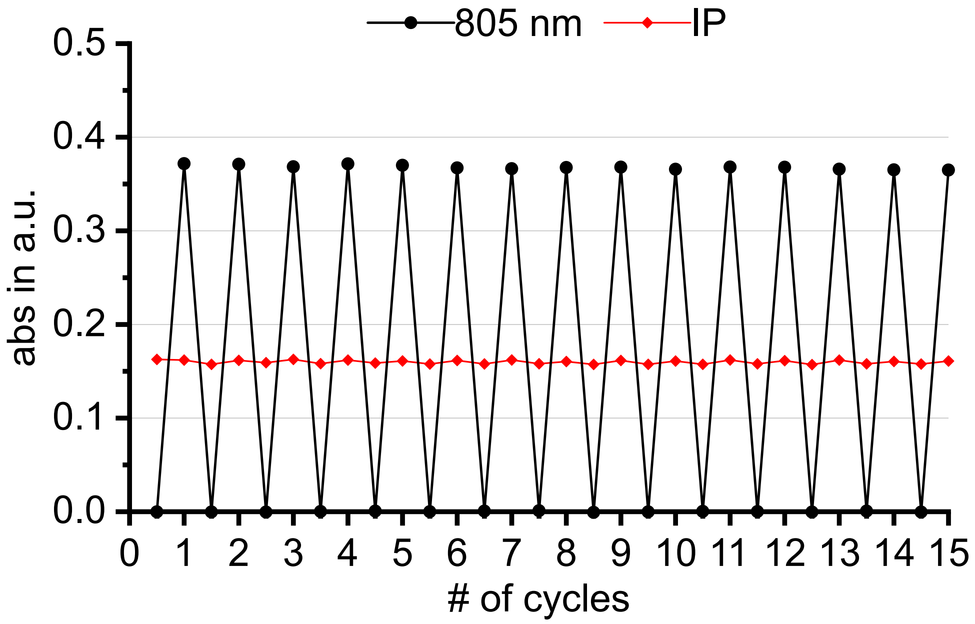

Figure S29 Absorption at 805 nm and the isosbestic point at 667 nm measured for each switching cycle of **PBFT** in THF solution. Alternating irradiation with 850 nm and 625 nm light was conducted for each cycle until the respective pss was reached.

Photoisomerization of PBFT with 905 nm NIR light

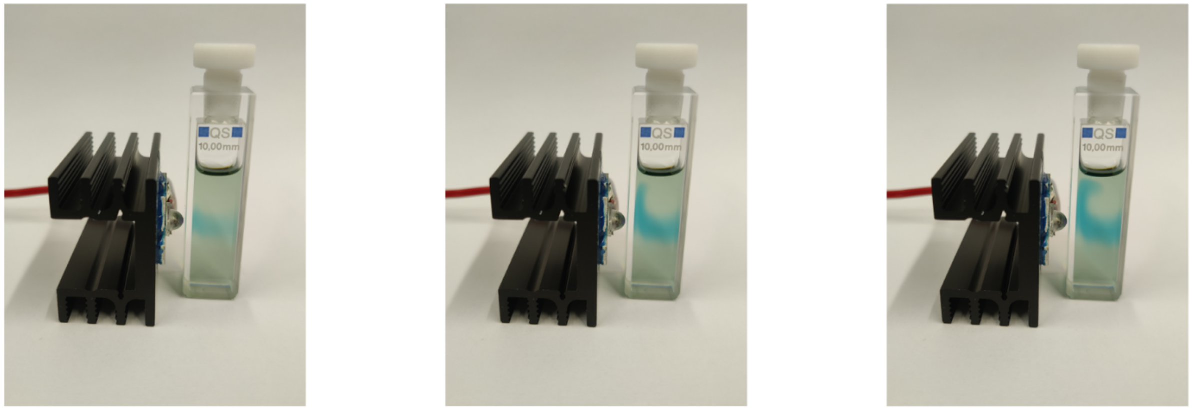

Figure S30 Irradiation of PBFT *E*-**PBFT** in benzene solution with a 905 nm LED (switched on) at 22 °C. The NIR light is not visible to the naked eye but facile photoswitching is observable via formation of the blue *E*-**PBFT** isomer.

Incorporation of PBFT into transparent 2D and 3D materials and reversible information inscription

Commercially available candle gel (Rayher, art. no. 3130200) was used as convenient and transparent solid matrix for all experiments in Petri dishes and cubes. The candle gel (100 g) was heated to 95 °C until it was completely liquid. **PBFT** (5 mg, 4.57 μmol) was dissolved in CH_2Cl_2 (7 mL) and added carefully to the hot solution. The mixture was stirred until **PBFT** was distributed equally in the solution and CH_2Cl_2 was evaporated completely. The solution was immediately poured into either Petri dishes or commercially available acrylic plastic cubes and dried for at least 16 h before performing any experiments. Any possible airbubbles that formed during pouring could easily be removed by annealing the candle gel in a drying oven at 60 °C for several hours.

After preparation and heating the **PBFT** containing transparent materials, only the *E* isomer was present giving the material a green appearance. Inscription with NIR 850 nm light was done using a flash-light, which converted *E*-**PBFT** to *Z*-**PBFT** within the solid matrix. The corresponding color change to deep blue *Z*-**PBFT** lead to a strong color contrast. Photomasks were printed on overhead transparencies for colour laser printers and three printed transparencies were combined on top of each other to prevent light leaking. Deletion of information was done using a 625 nm LED in combination with either printed overhead transparencies or black cardboard cutouts. For 3D displays, inscription with 850 nm light was done at one face of the cube and deletion with 625 nm light was done either at one face at 90° to the inscription face or at both faces, each at 90° to the inscription face.

Notably, the thermal stability of the metastable *Z*-**PBFT** isomers is significantly improved within the solid matrix. This is most clearly seen when a mixture of **PBFT** and candle gel were heated to 97 °C and then irradiated at that high temperature with 850 nm light (Movie 11), which led to facile conversion to the blue *Z*-**PBFT** isomer. After switching off the light more than 30 s were needed to return the heated material to the *E*-**PBFT** isomeric state.

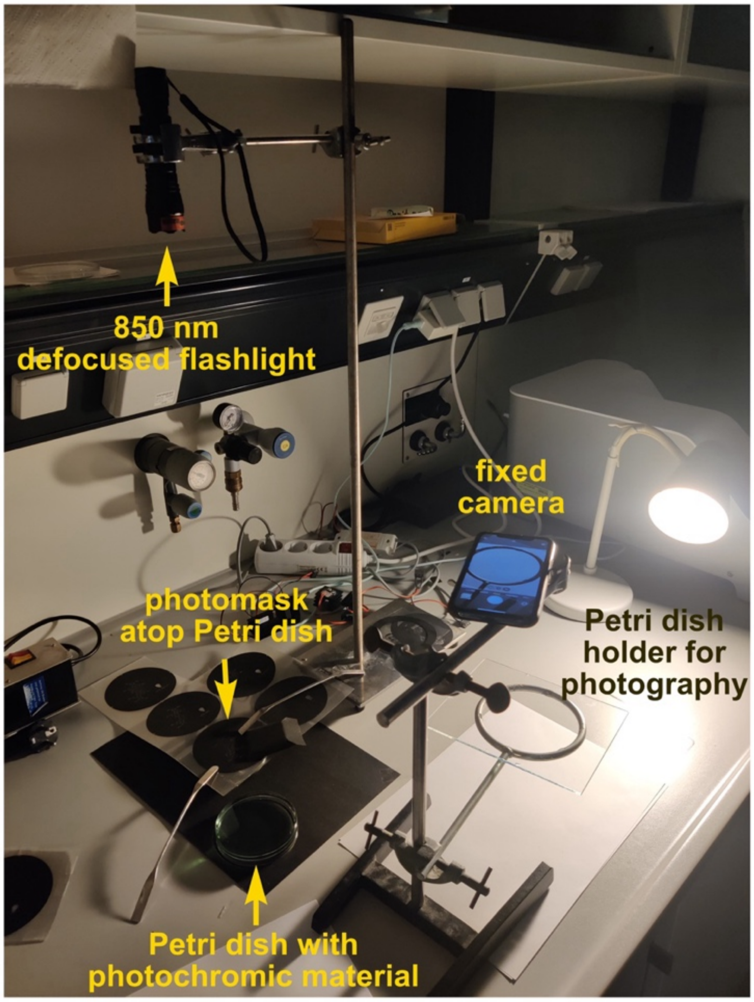

Figure S31 Irradiation setup for reversible information inscription with NIR and red light into a 2D material inside handheld Petri dishes.

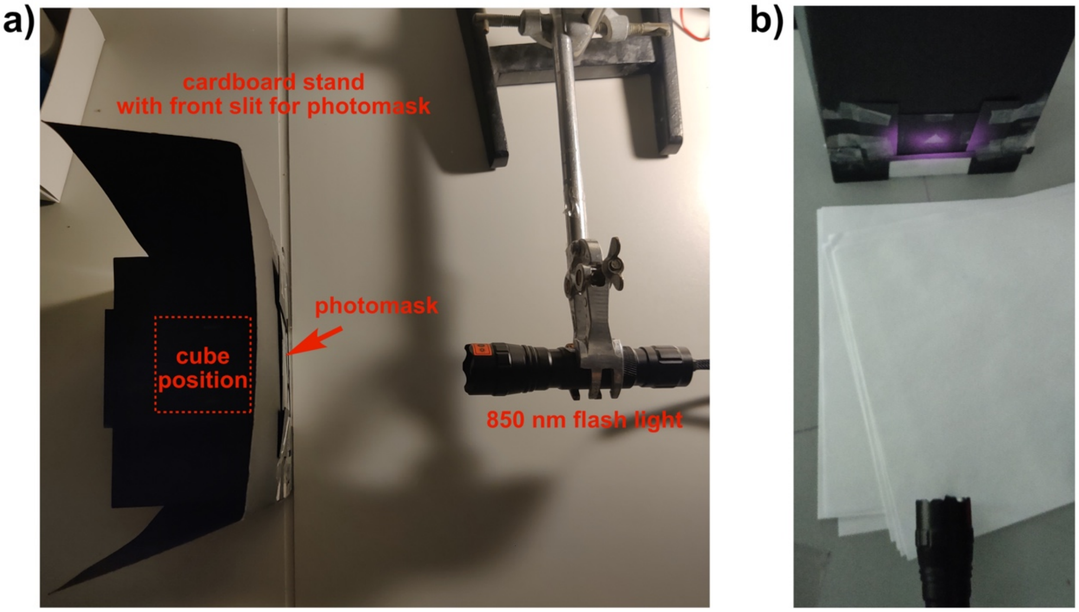

Figure S32 Irradiation setup for information inscription with 850 nm NIR light into handheld 3D cubes. a) General setup. b) Setup photographed during 850 nm irradiation showing the NIR light beam centered at the triangular shape of the photomask. The light is only visible through the smartphone camera.

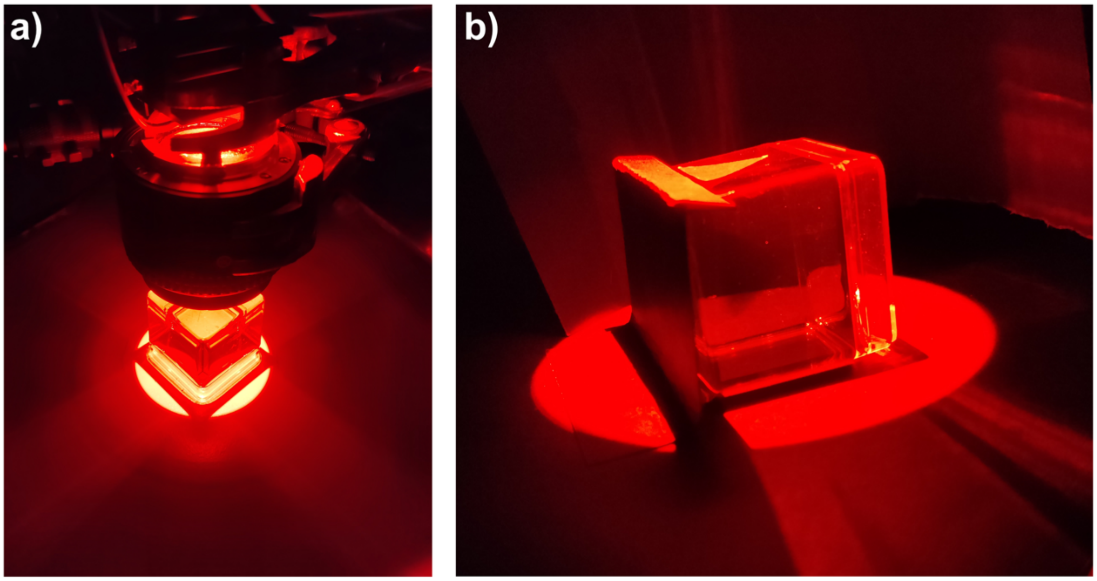

Figure S33 Irradiation setup for information deletion with 625 nm red light in handheld 3D cubes. a) The red light LED was focused through a 50 mm camera lens. b) Black photomask or cardboard cutouts were used to define non-deletion areas.

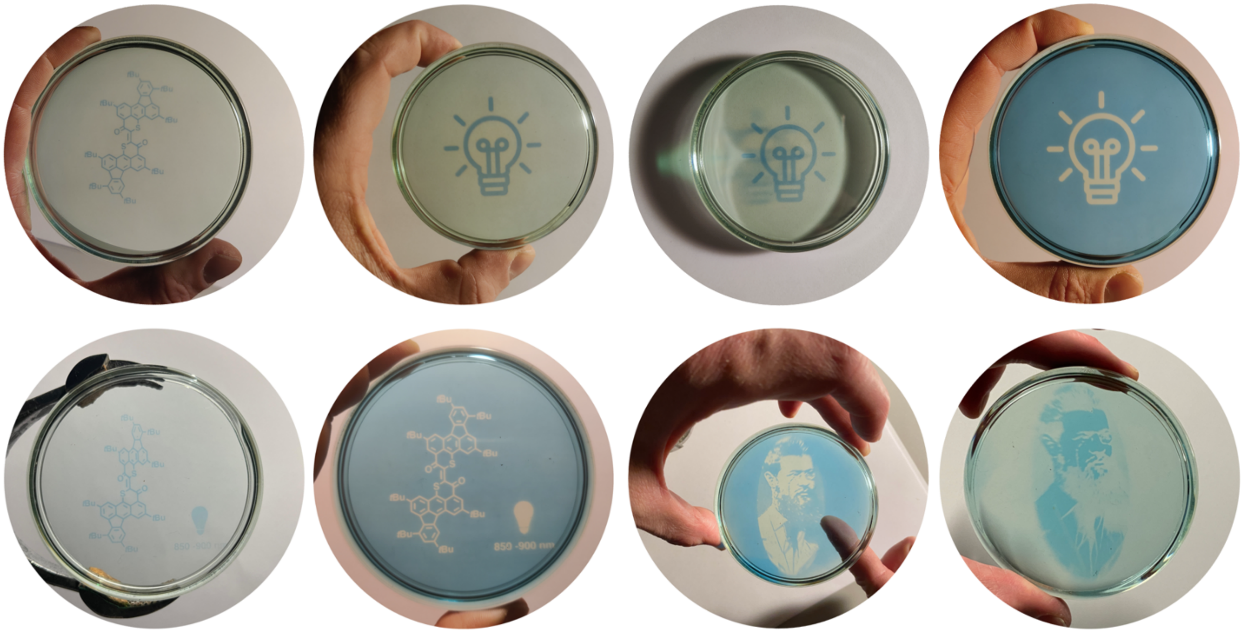

Figure S34 2D information inscription into transparent photochromic candle gel containing **PBFT** using NIR light.

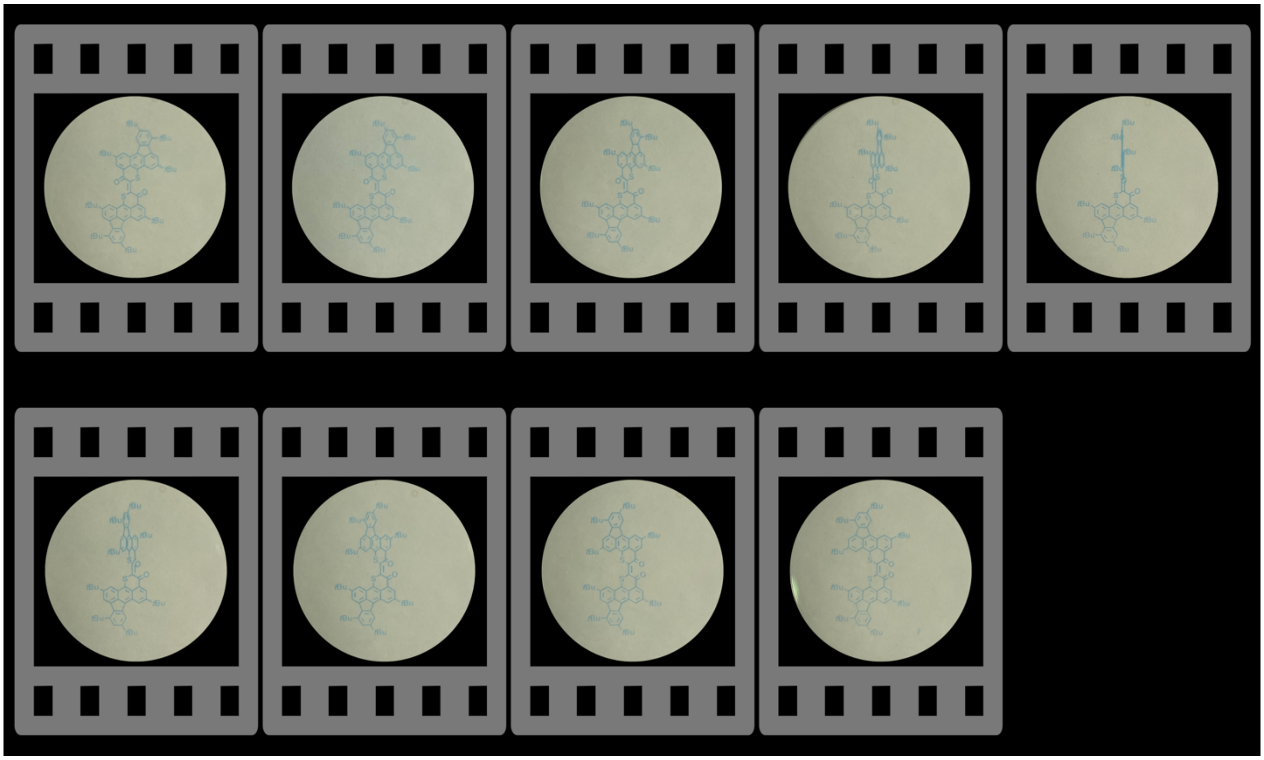

Figure S35 Individual movie frames of **PBFT** rotation repeatedly inscribed with 850 nm light into the same photochromic 2D material.

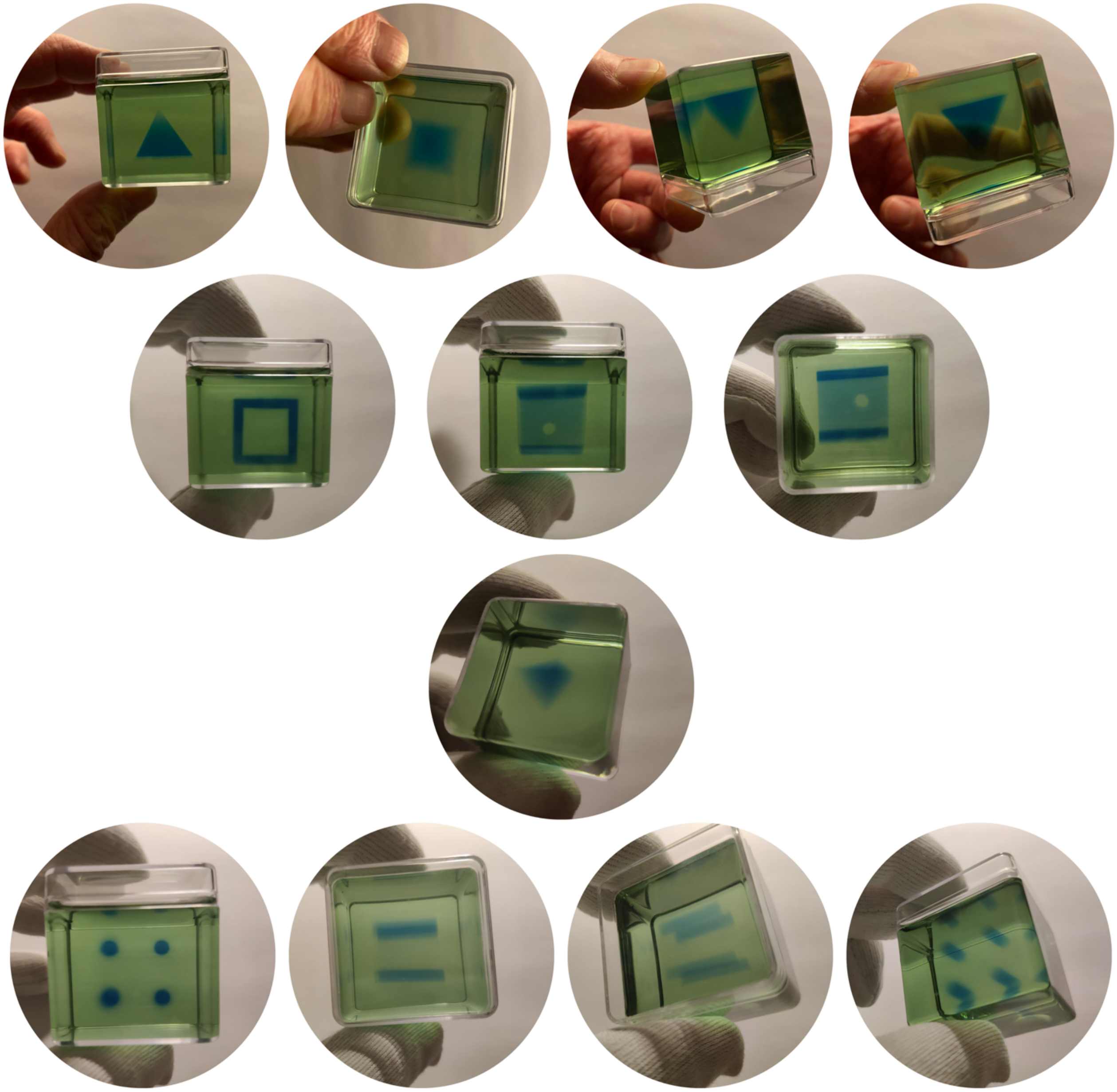

Figure S36 3D information inscription into acrylic plastic cubes containing transparent candle gel and **PBFT**. 850 nm NIR light was used for inscription and 625 nm light was used for deletion and shape definition. First row: 3D pyramid shape. Second row: 3D square tube with two circular openings at each side. Third row: 3D tetrahedron. Fourth row: 3D rod arrangement.

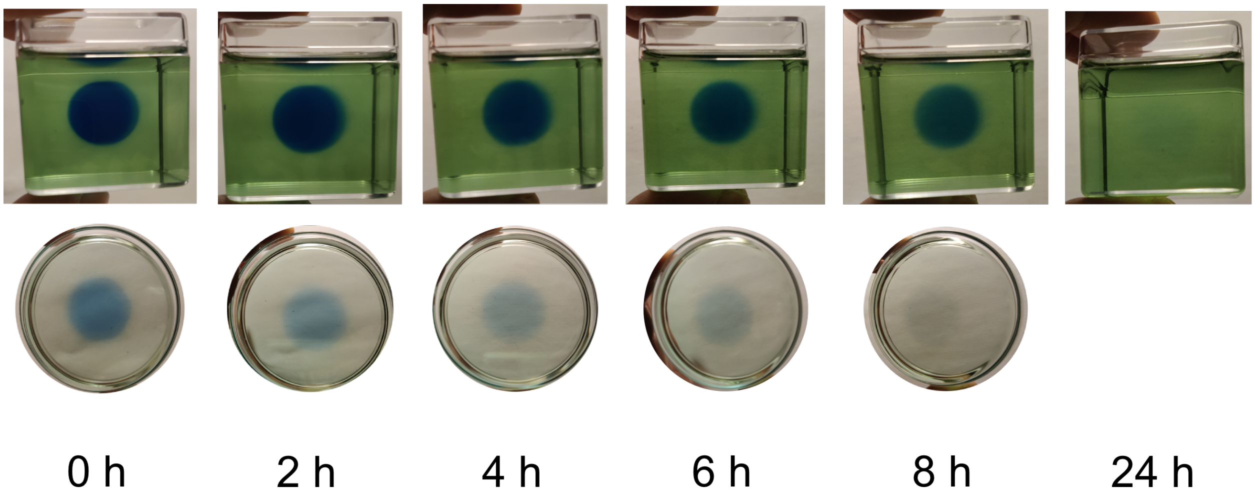

Figure S37 3D blue inscribed *Z* isomeric **PBFT** shows extended lifetime within the candle gel up to 24 h at ambient temperature. After inscription, the cube was kept in the dark at 22 °C for the indicated time.

To upscale the reversible 3D displays and to highlight their very good performance and easy preparation and handling, 12.5 cm x 12.5 cm x 12.5 cm transparent cubes containing ~1.5 L of the candle gel matrix and ~20 mg of **PBFT** were prepared. The cubes were prepared by following the same procedure as given above for their smaller counterparts.

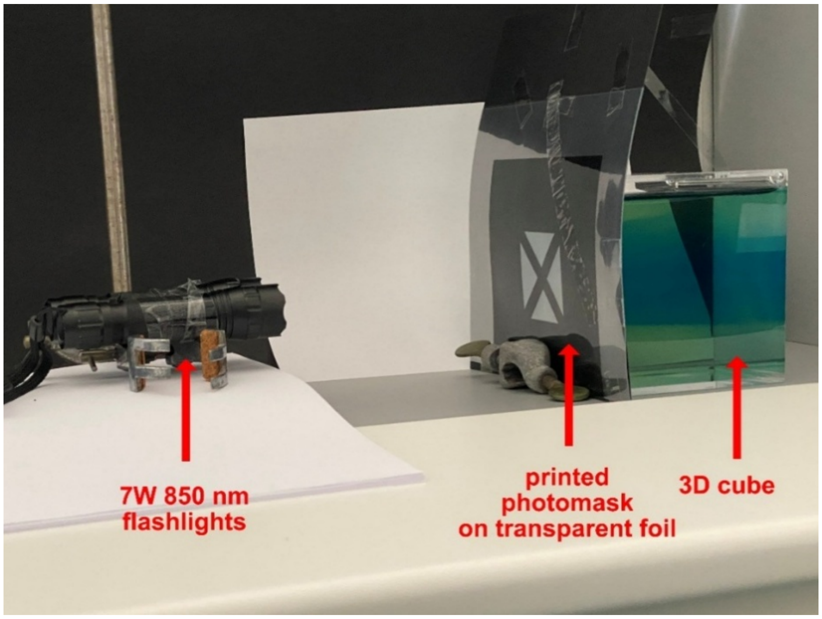

Figure S38 Irradiation setup for information inscription with 850 nm NIR light into large size 3D cubes containing 1.5 L of material. Two 7 W 850 nm flashlights were used irradiating through a laser-printed photomask to write information into the cube.

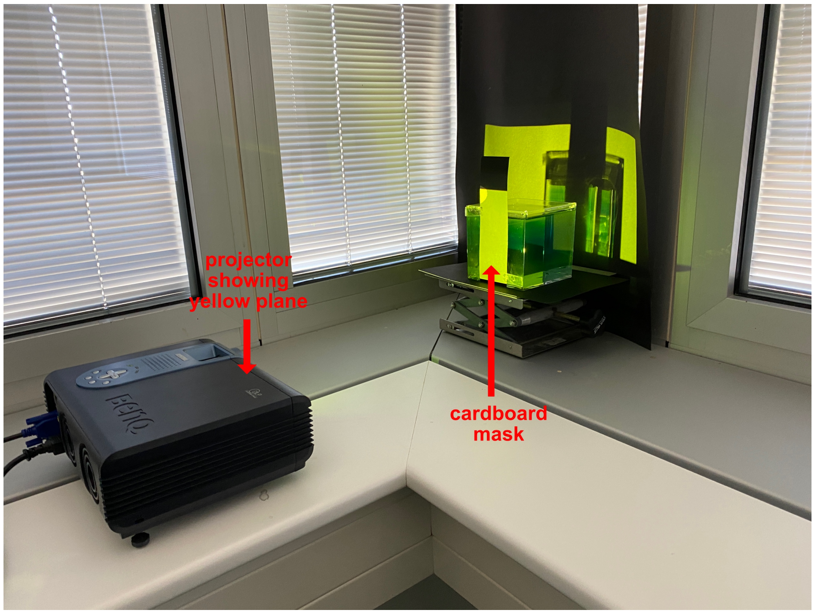

Figure S39 Irradiation setup for large-scale deletion of 3D information with yellow light using a regular projector and a cardboard cutout as inverse photomask.

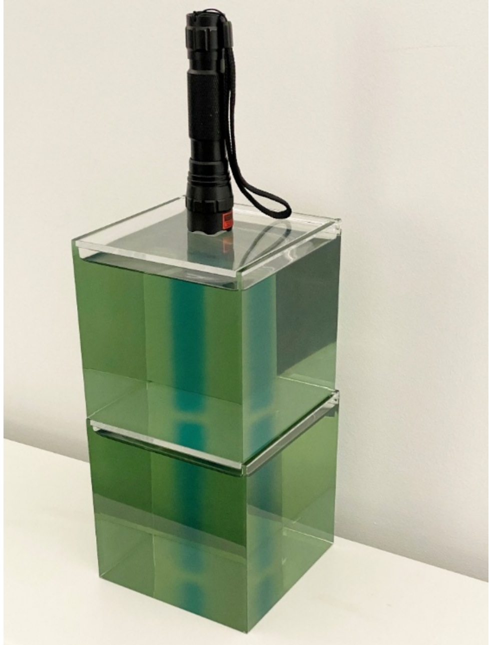

Figure S40 Two large sized cubes (12.5 cm x 12.5 cm x 12.5 cm) stacked on top of each other to test the penetration depth of NIR light. After 1 min of irradiation with an 850 nm flashlight, full penetration of both cubes is achieved.

NMR spectra

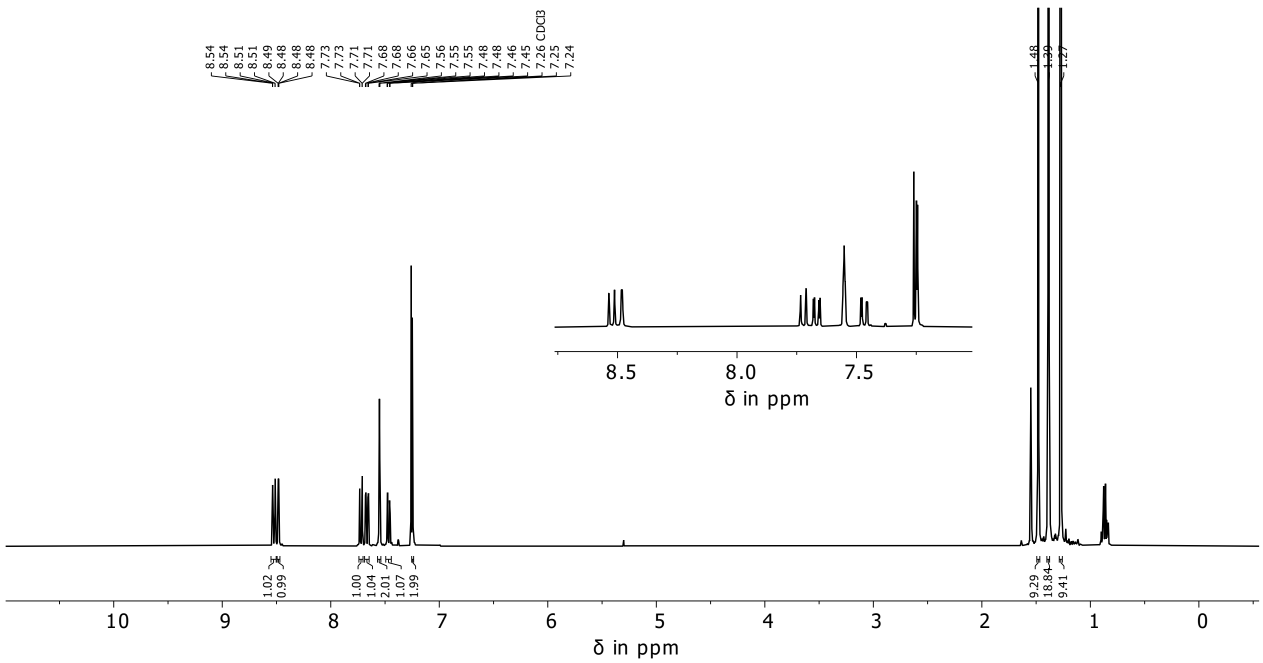

Figure S41 ^{1}H NMR spectrum of **2** (400 MHz, $CDCl_3$, 23 °C).

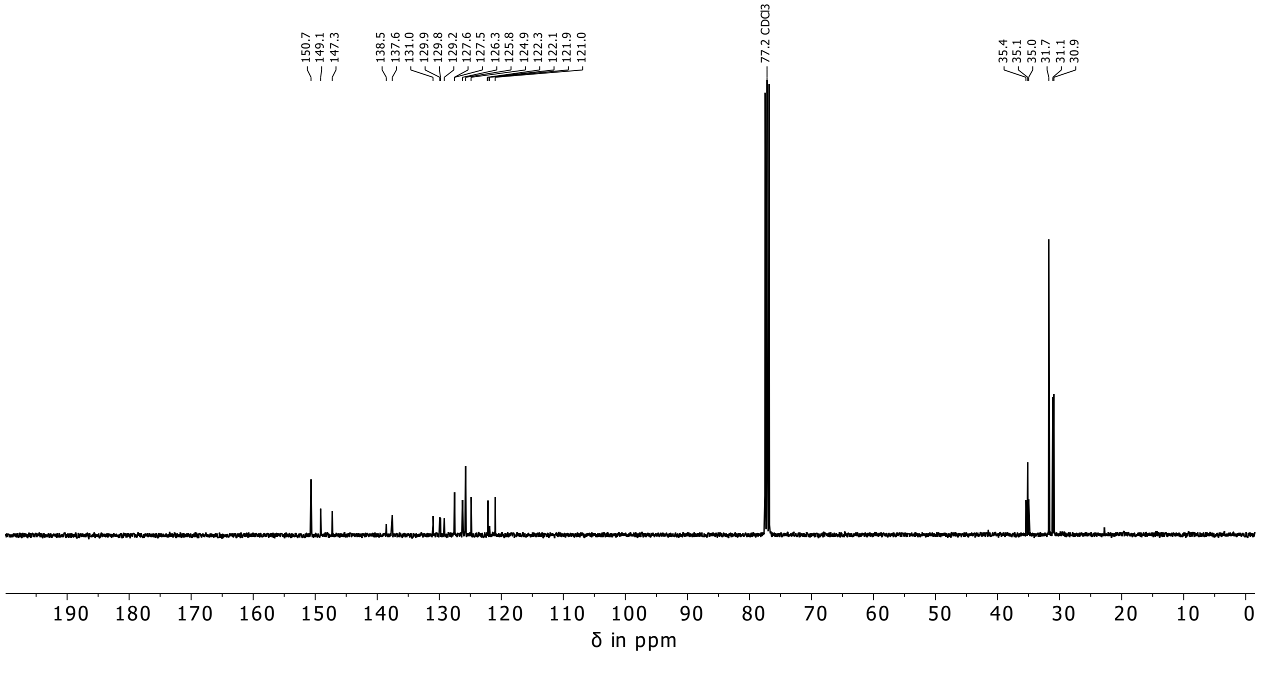

Figure S42 ^{13}C NMR spectrum of **2** (101 MHz, $CDCl_3$, 23 °C).

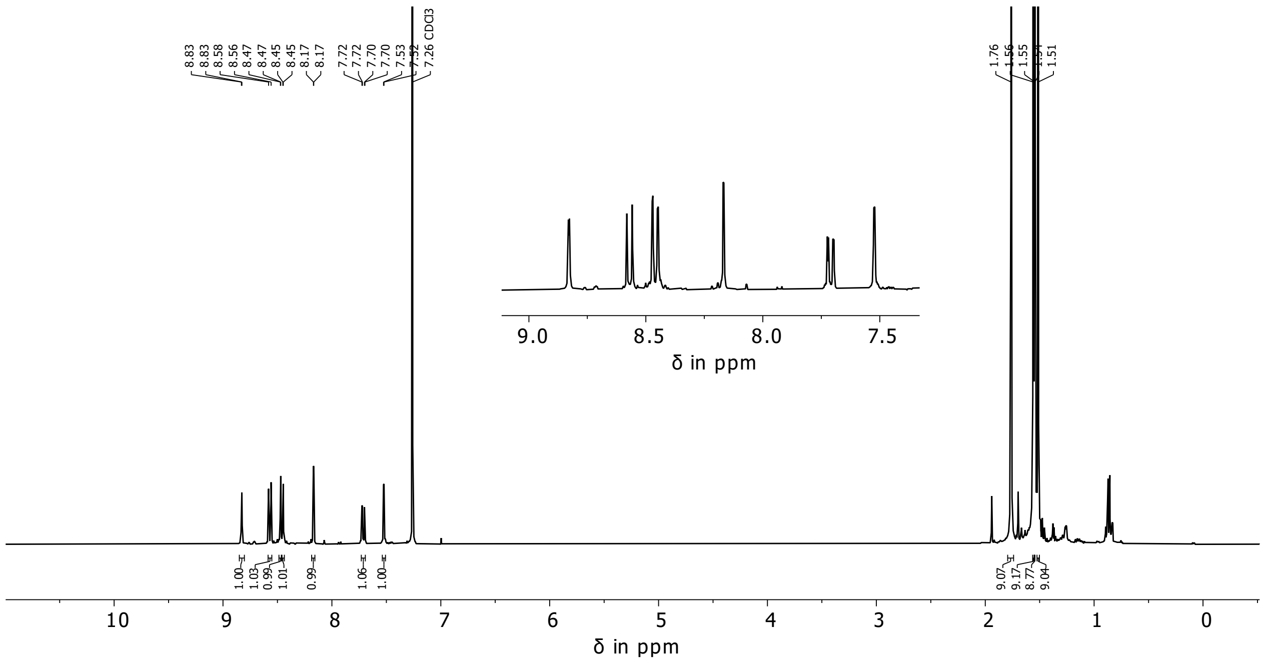

Figure S43 ^{1}H NMR spectrum of **3** (400 MHz, $CDCl_3$, 23 °C).

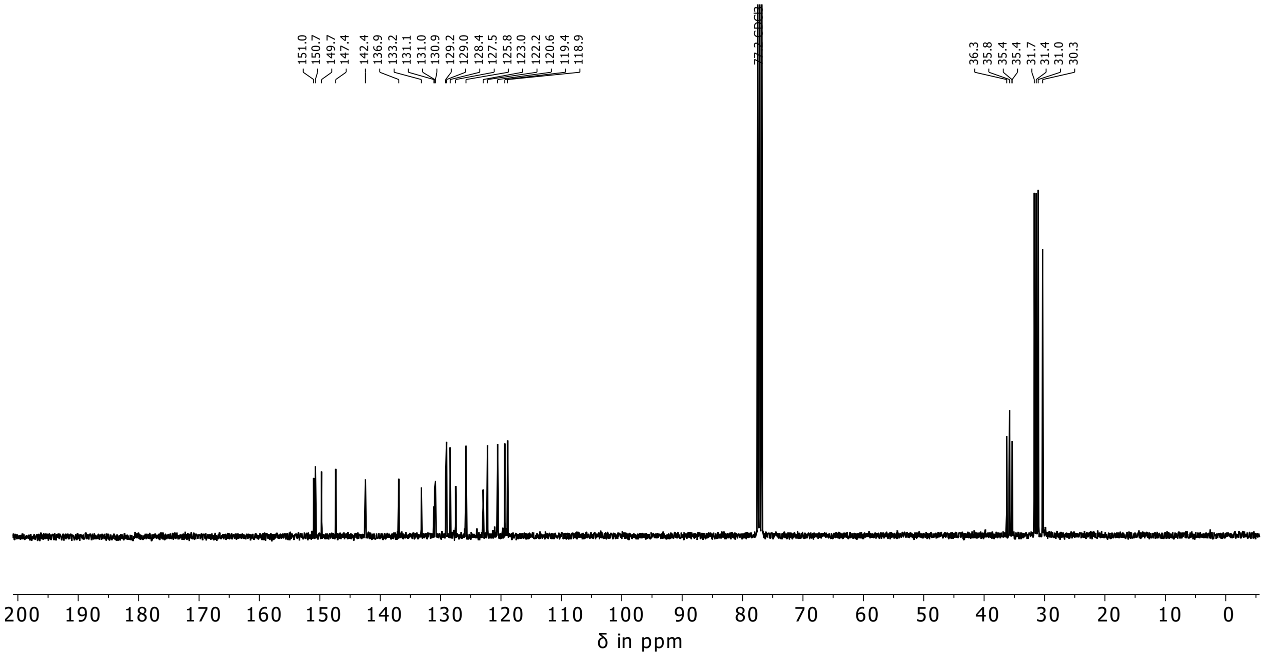

Figure S44 ^{13}C NMR spectrum of **3** (101 MHz, $CDCl_3$, 23 °C).

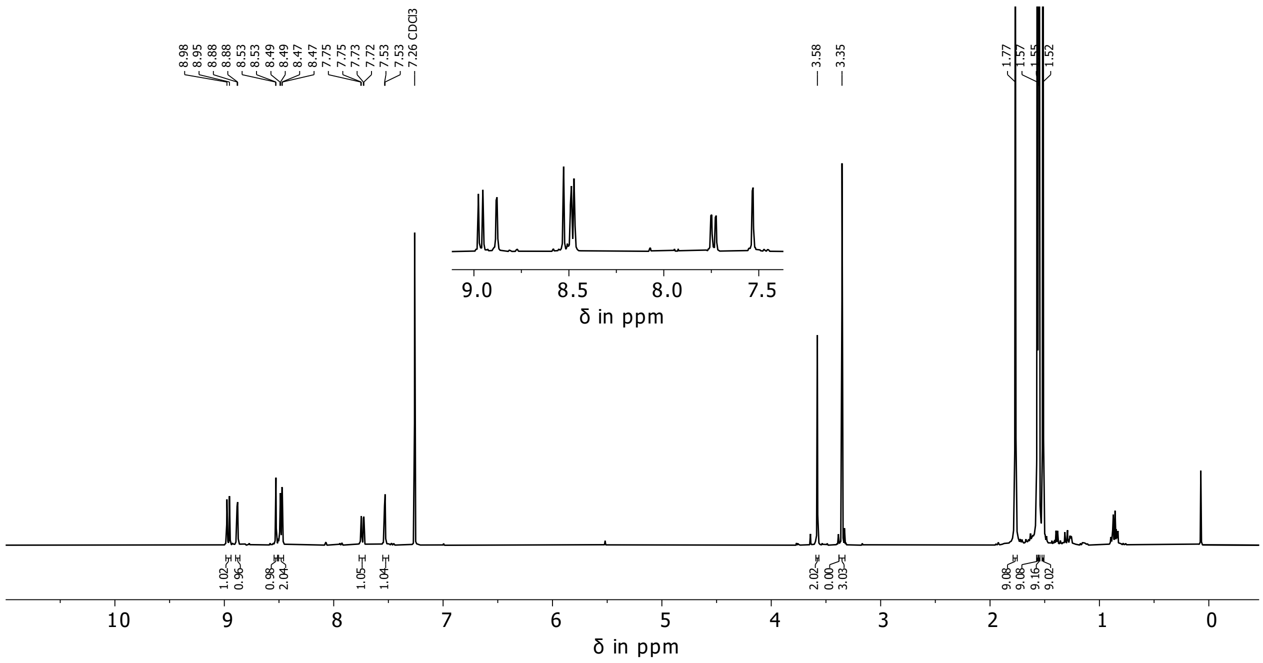

Figure S45 ^{1}H NMR spectrum of **4** (400 MHz, $CDCl_3$, 23 °C).

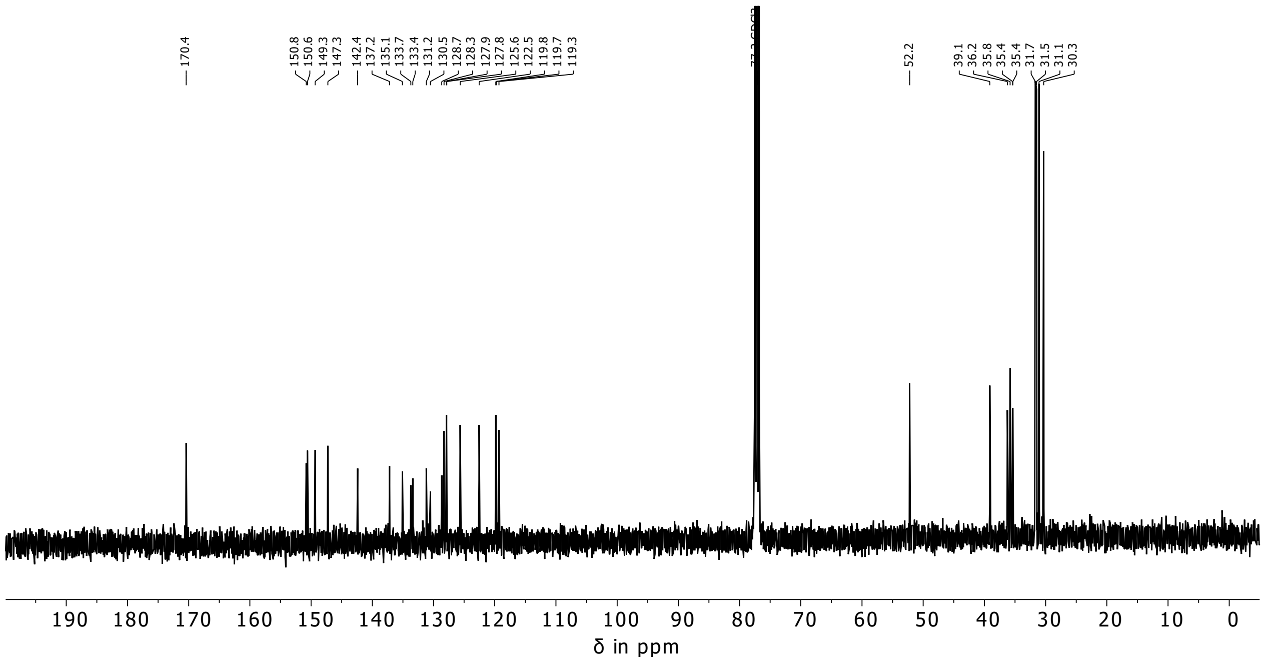

Figure S46 ^{13}C NMR spectrum of **4** (101 MHz, $CDCl_3$, 23 °C).

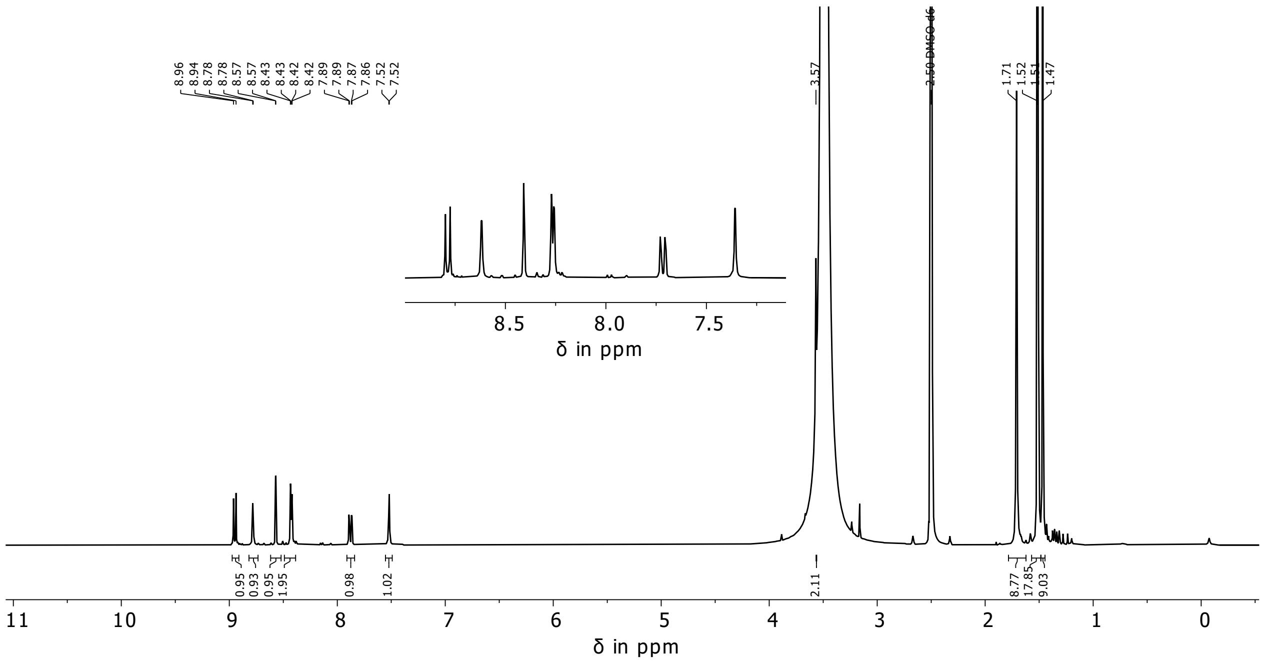

Figure S49 ^{1}H NMR spectrum of **5** (400 MHz, DMSO-d_6, 23 °C).

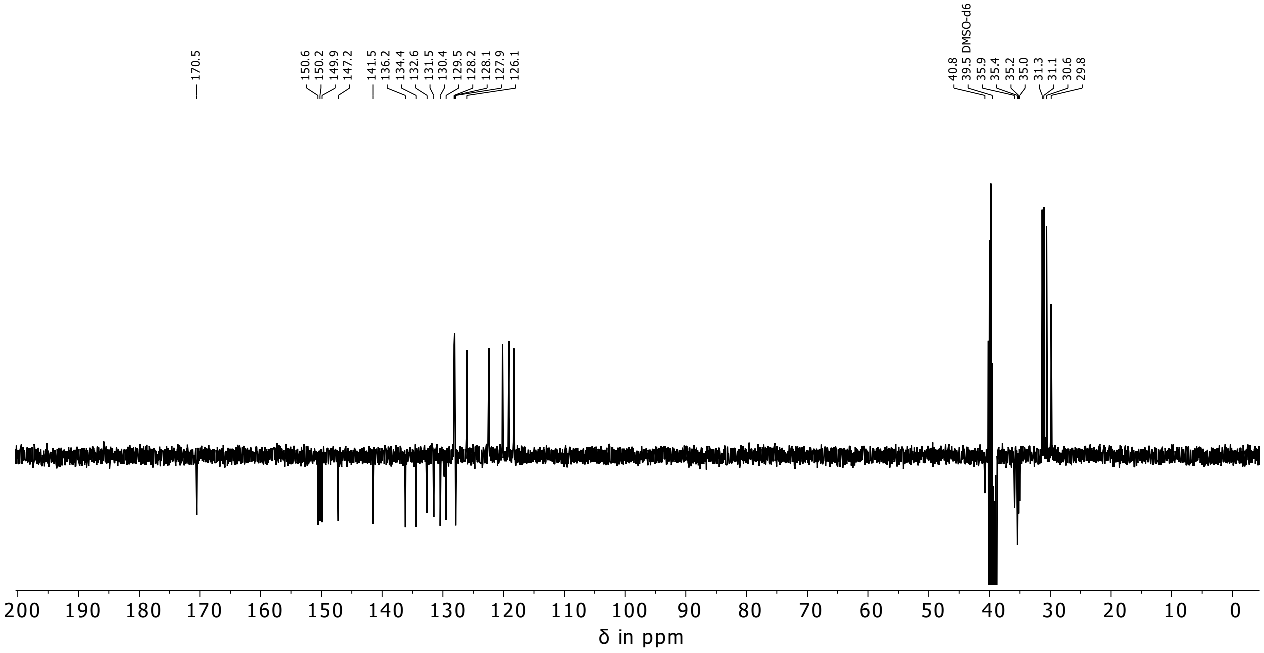

Figure S50 ^{13}C(DEPTQ) NMR spectrum of **5** (101 MHz, DMSO-d_6, 23 °C).

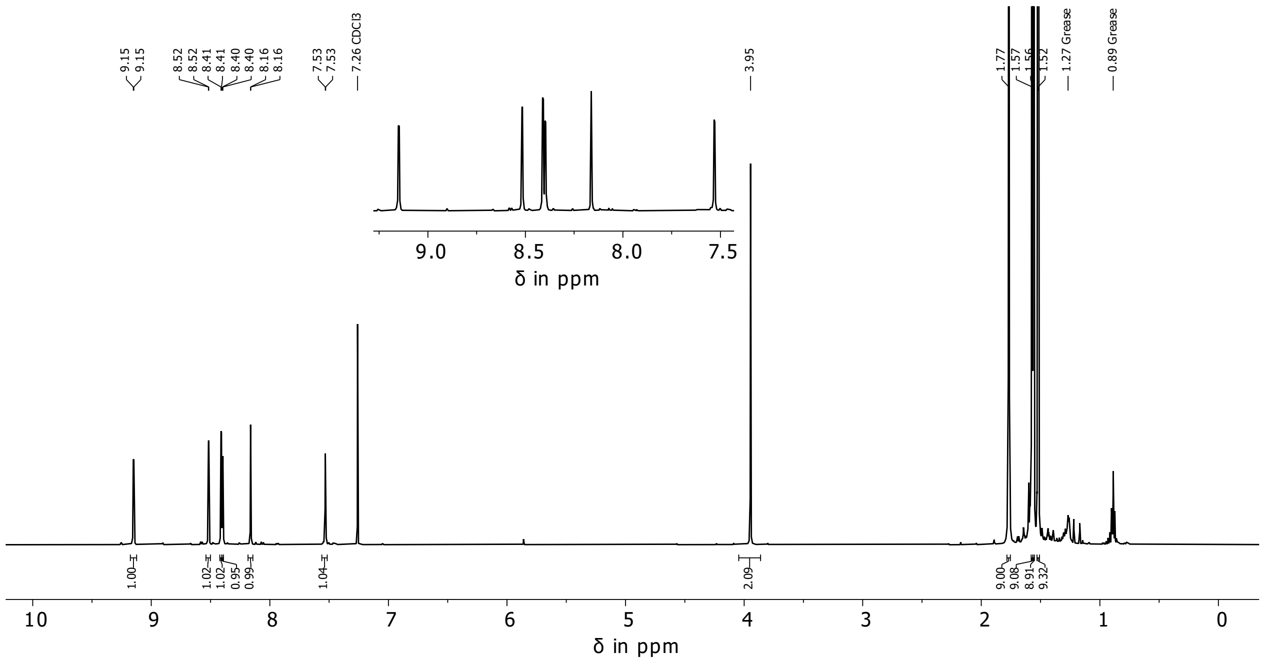

Figure S51 ^{1}H NMR spectrum of **6** (400 MHz, $CDCl_3$, 23 °C).

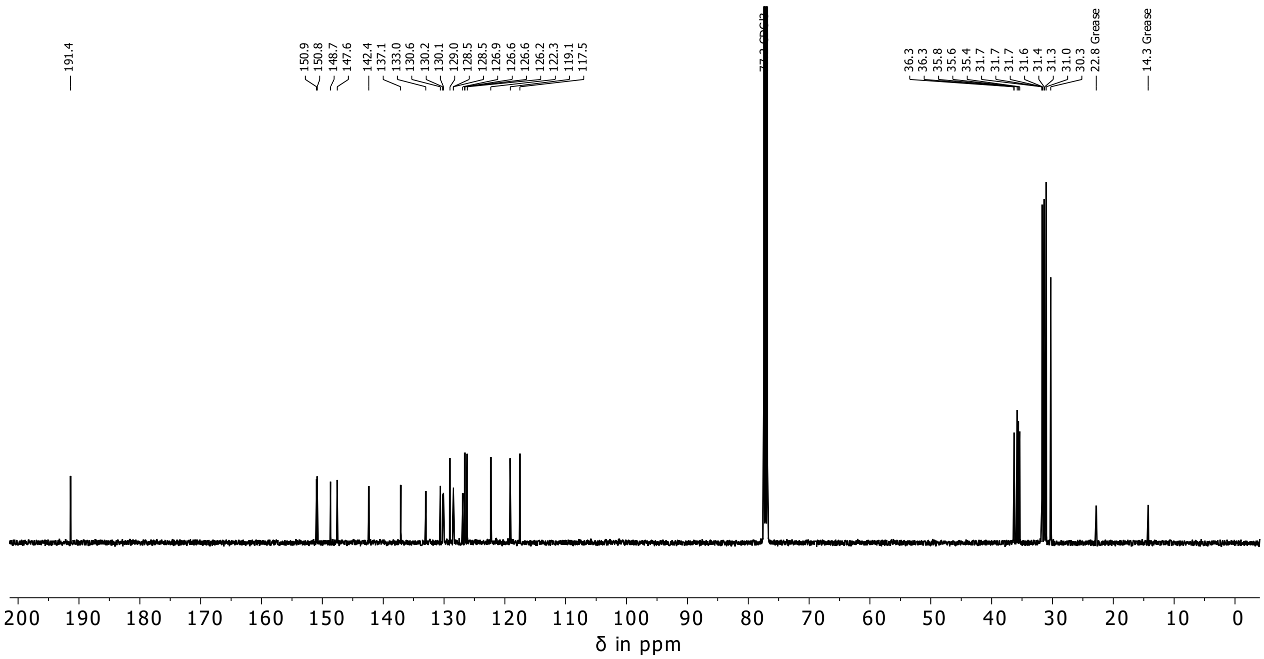

Figure S52 ^{13}C NMR spectrum of **6** (101 MHz, $CDCl_3$, 23 °C).

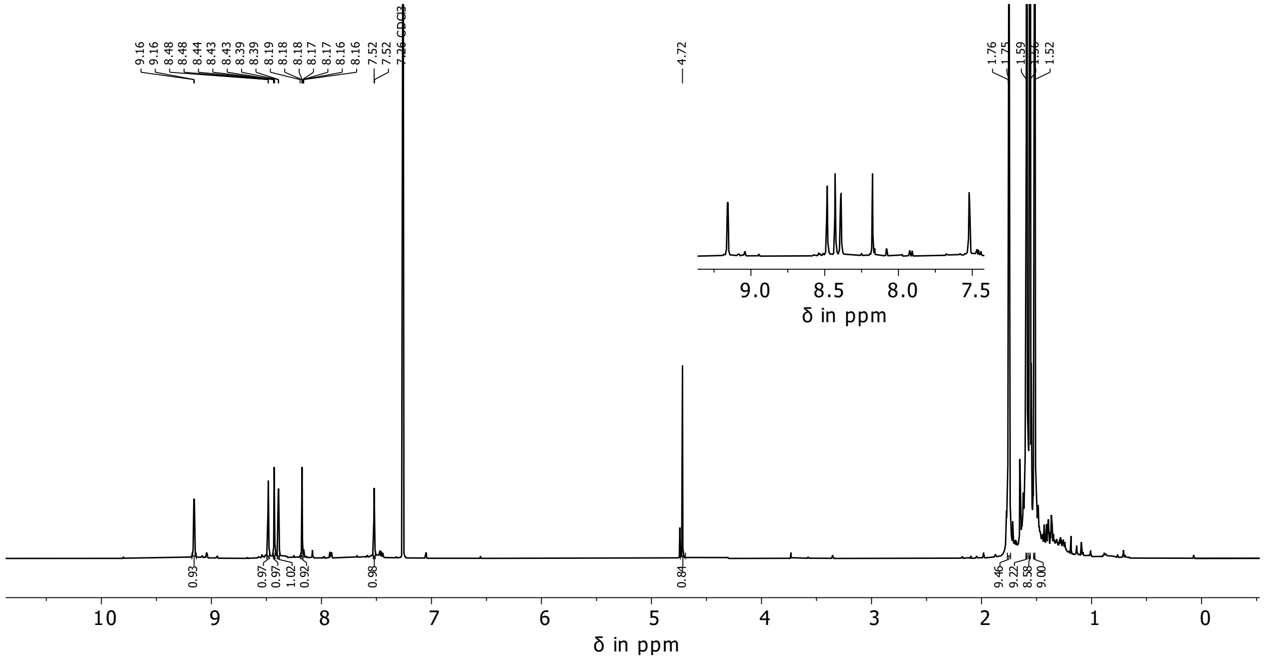

Figure S53 ^{1}H NMR spectrum of **7a** (400 MHz, $CDCl_3$, 23 °C).

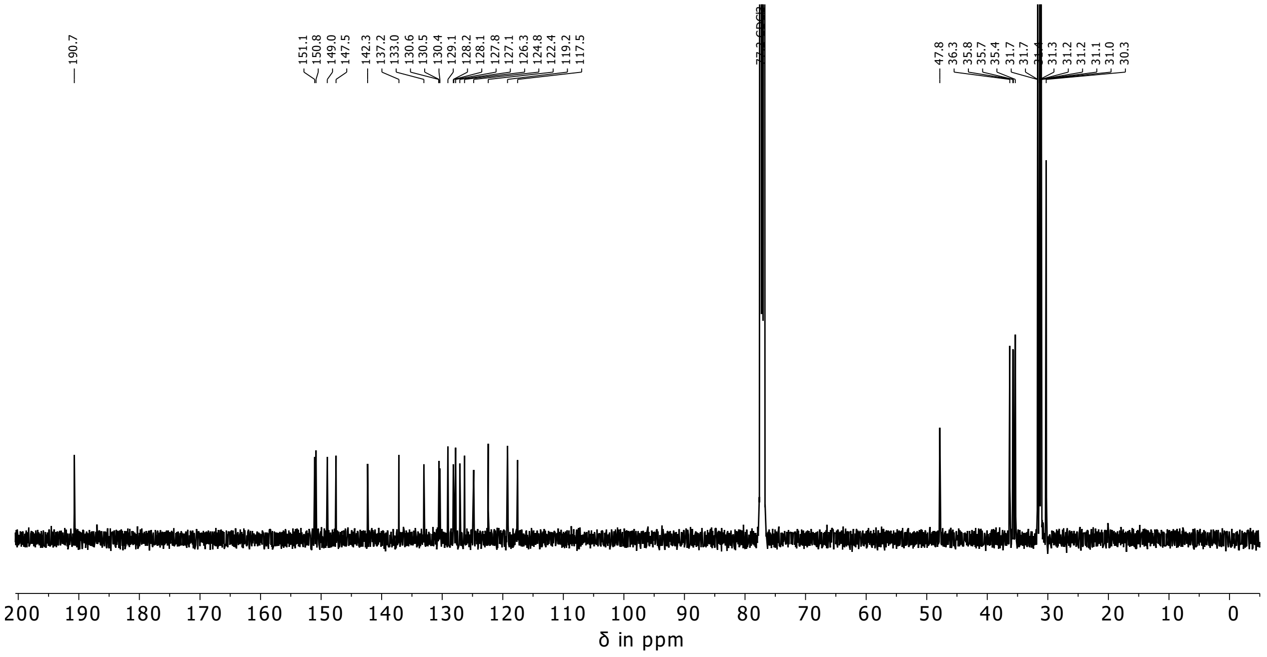

Figure S54 ^{13}C NMR spectrum of **7a** (101 MHz, $CDCl_3$, 23 °C).

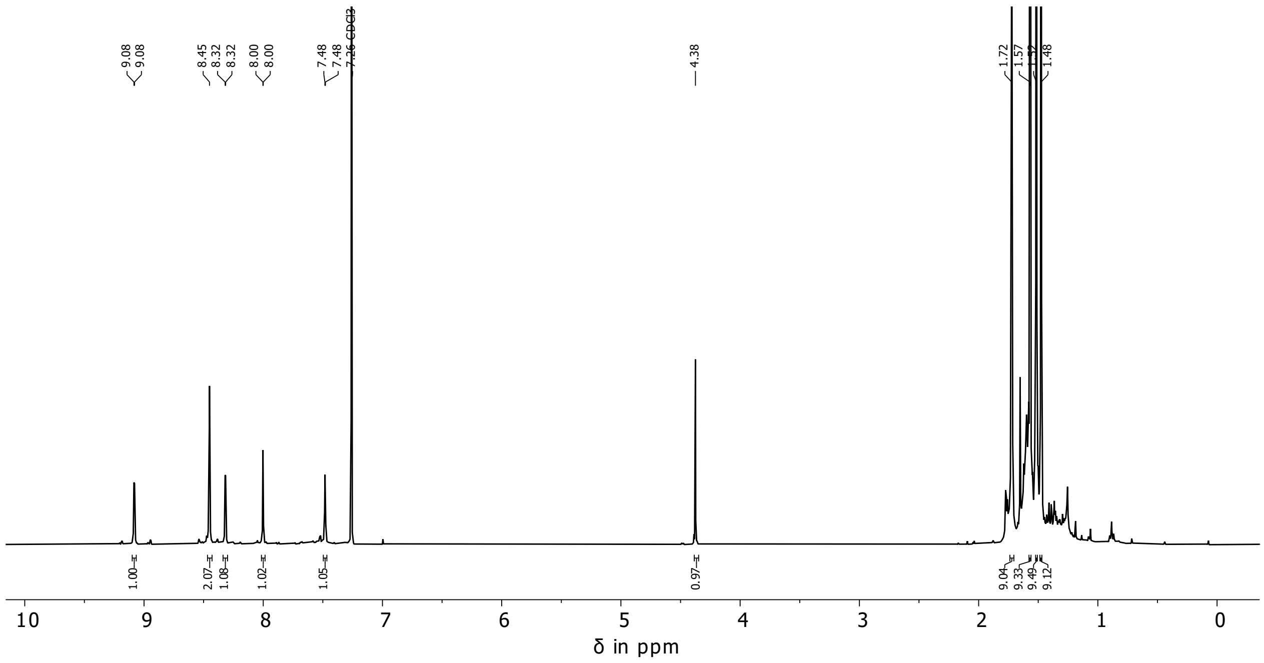

Figure S55 ^{1}H NMR spectrum of **7b** (400 MHz, $CDCl_3$, 23 °C).

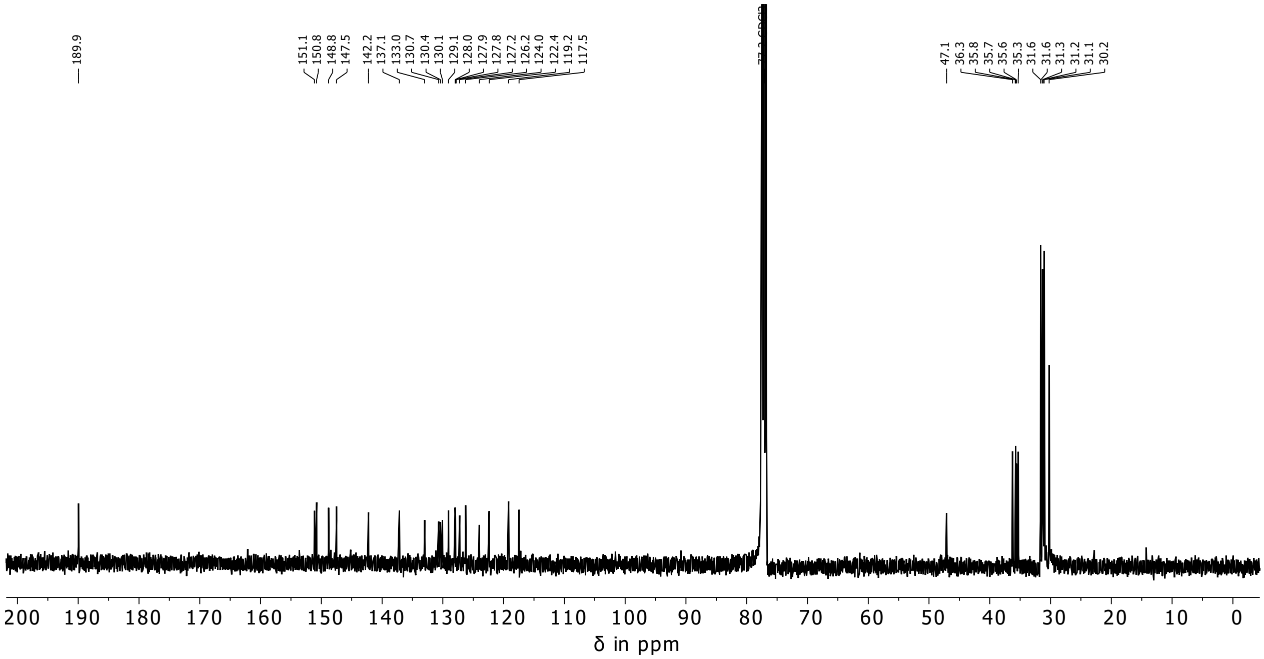

Figure S56 ^{13}C NMR spectrum of **7b** (101 MHz, $CDCl_3$, 23 °C).

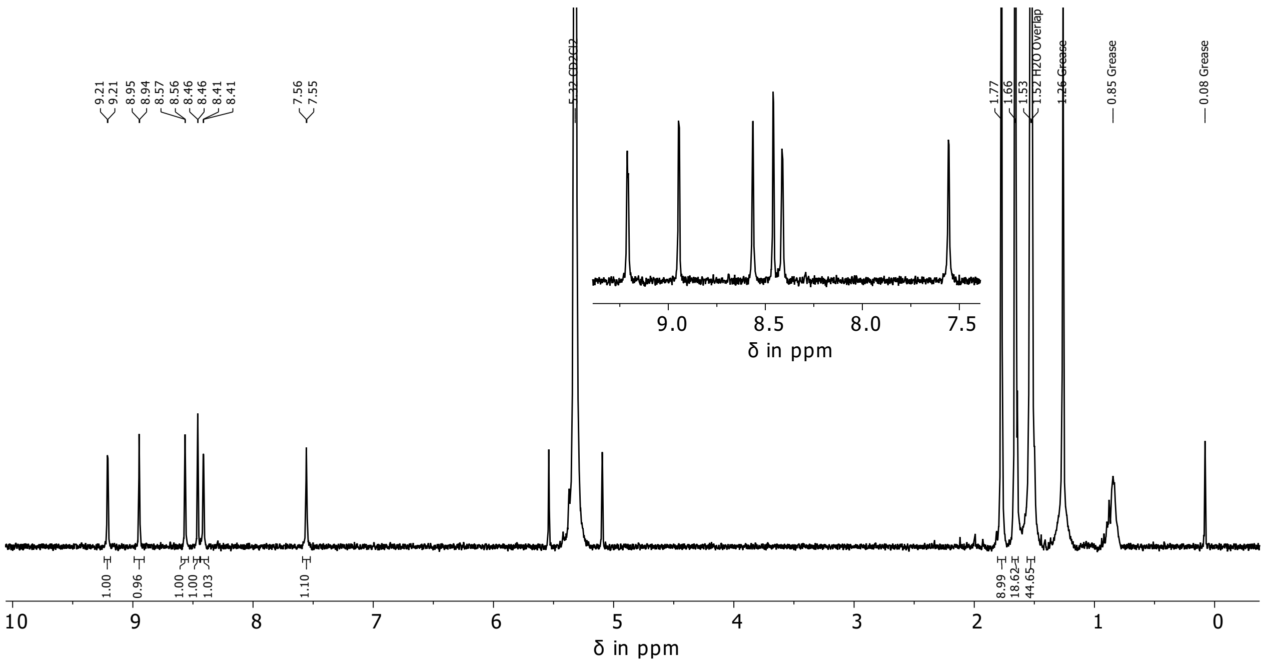

Figure S57 ^{1}H NMR spectrum of *E*-**PBFT** (400 MHz, CD_2Cl_2, 23 °C).

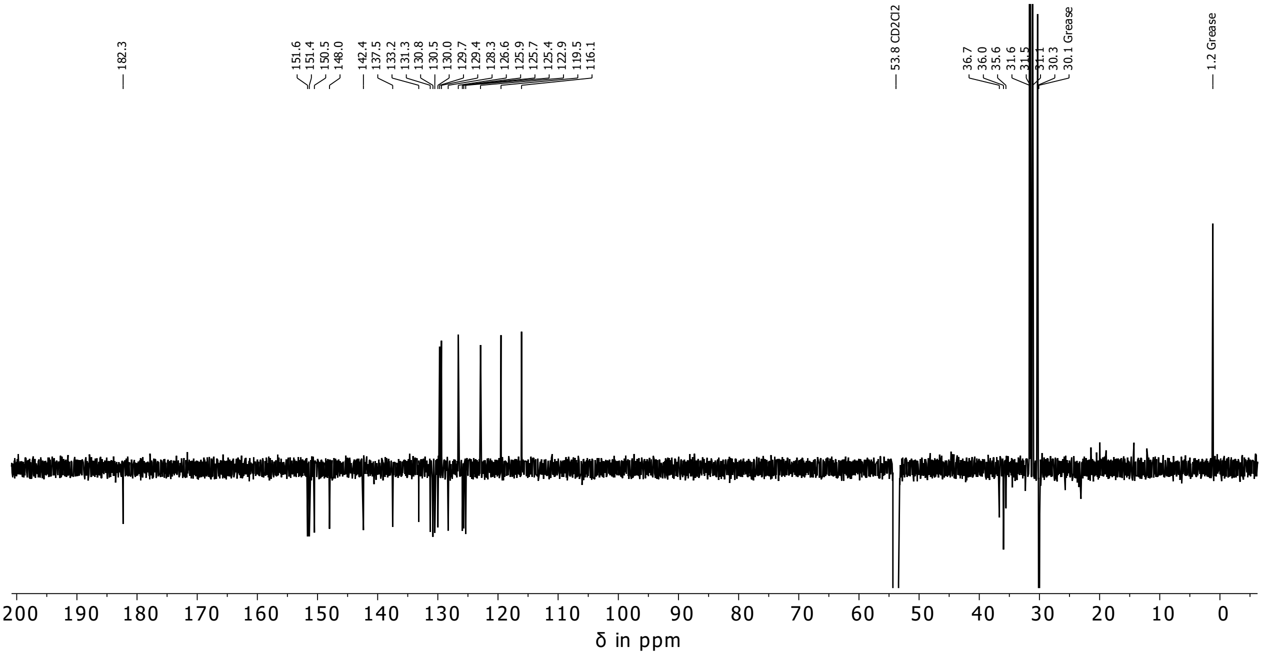

Figure S58 ^{13}C(DEPTQ) spectrum of *E*-**PBFT** (600 MHz, CD_2Cl_2, 23 °C).

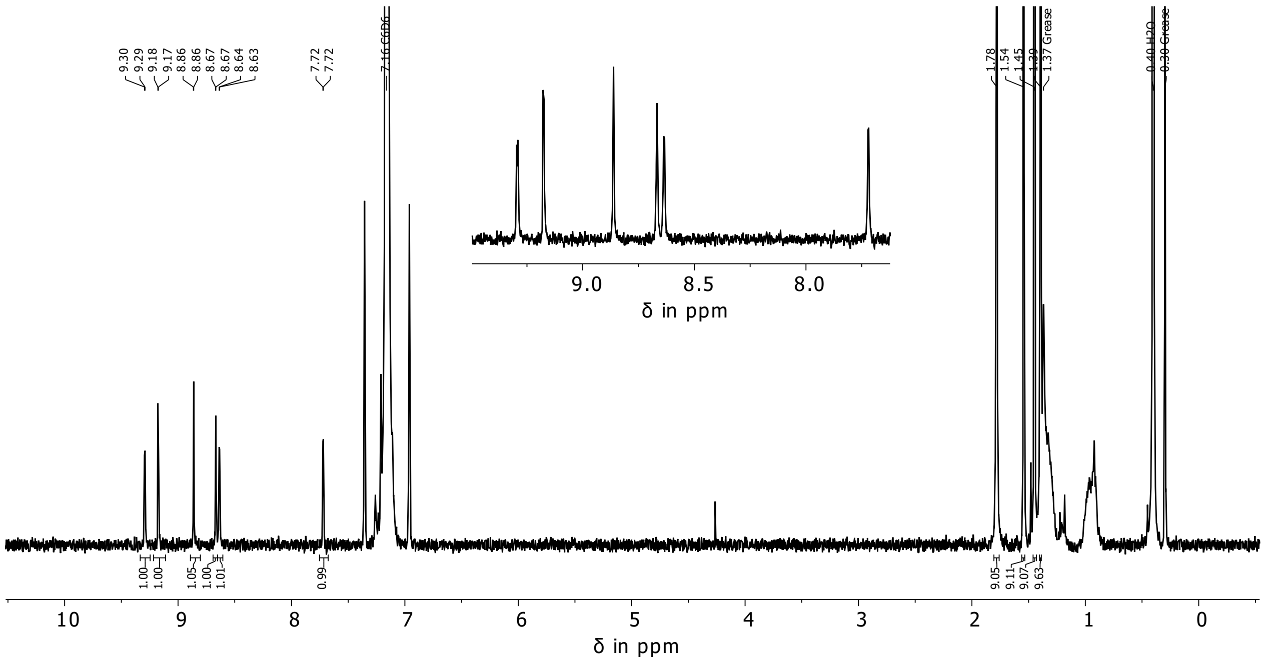

Figure S59 ^{1}H NMR spectrum of *E*-**PBFT** (400 MHz, C_6D_6, 23 °C). Substantial amount of additional grease stems from the NMR solvent.

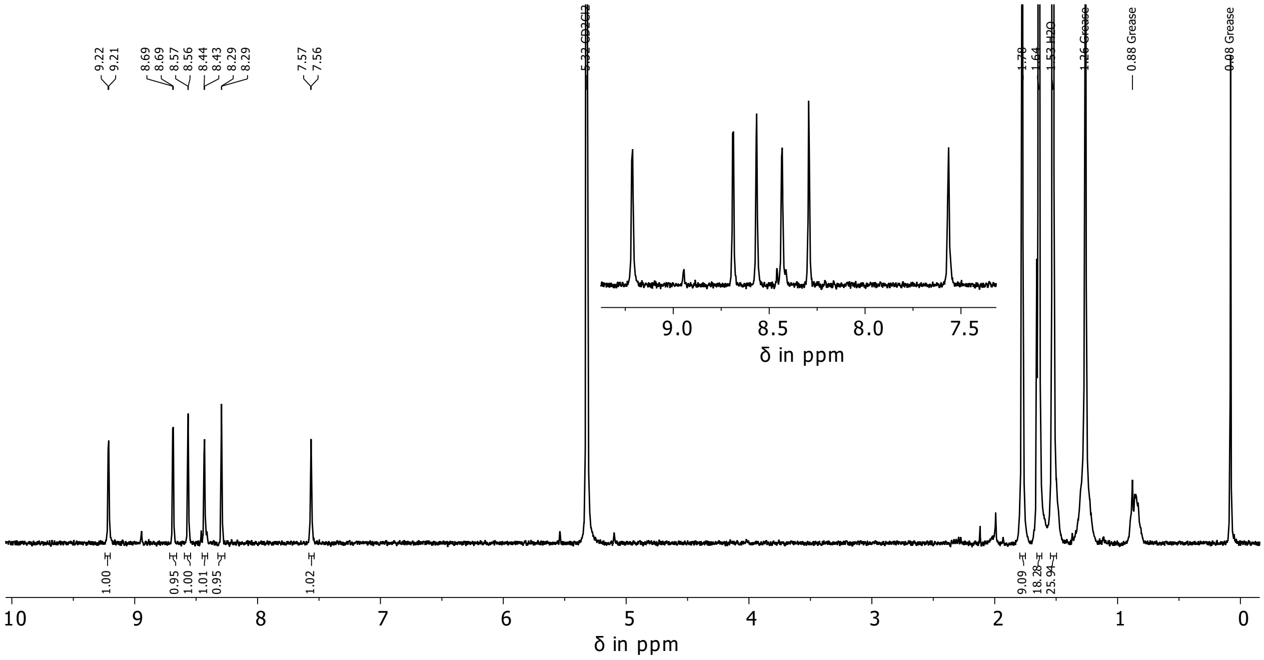

Figure S60 ^{1}H NMR spectrum of *Z*-**PBFT** (400 MHz, CD_2Cl_2, 23 °C).

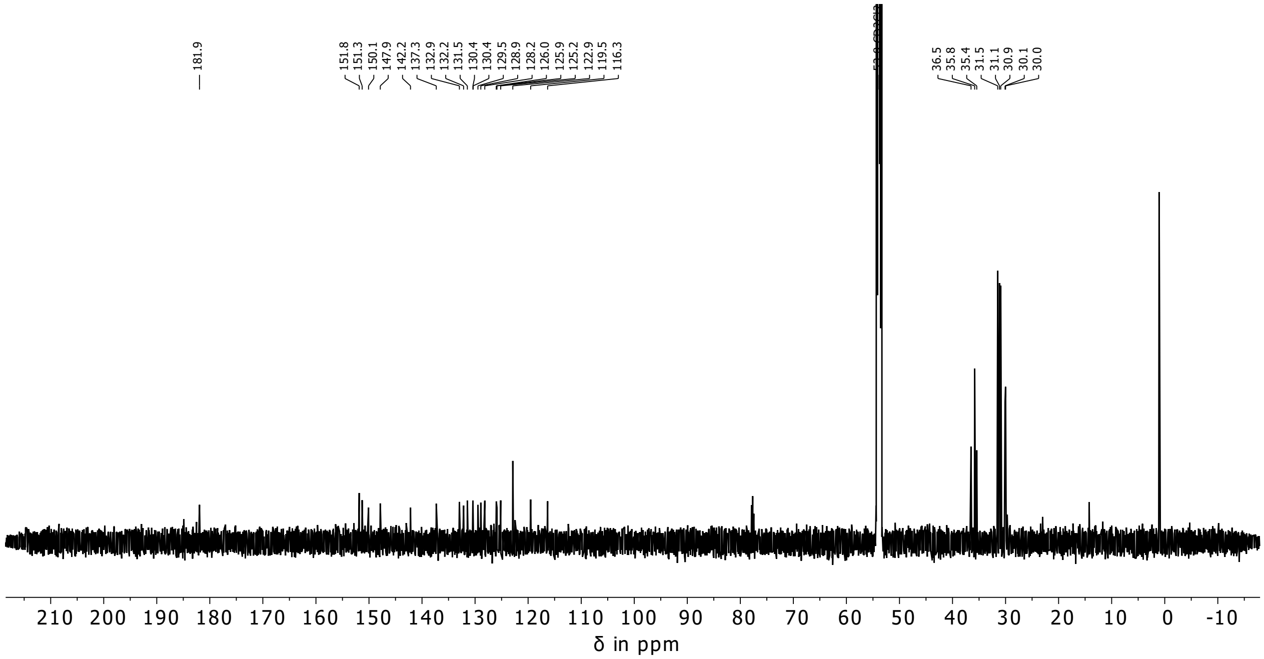

Figure S61 ^{13}C NMR spectrum of *Z*-**PBFT** (151 MHz, CD_2Cl_2, 23 °C).

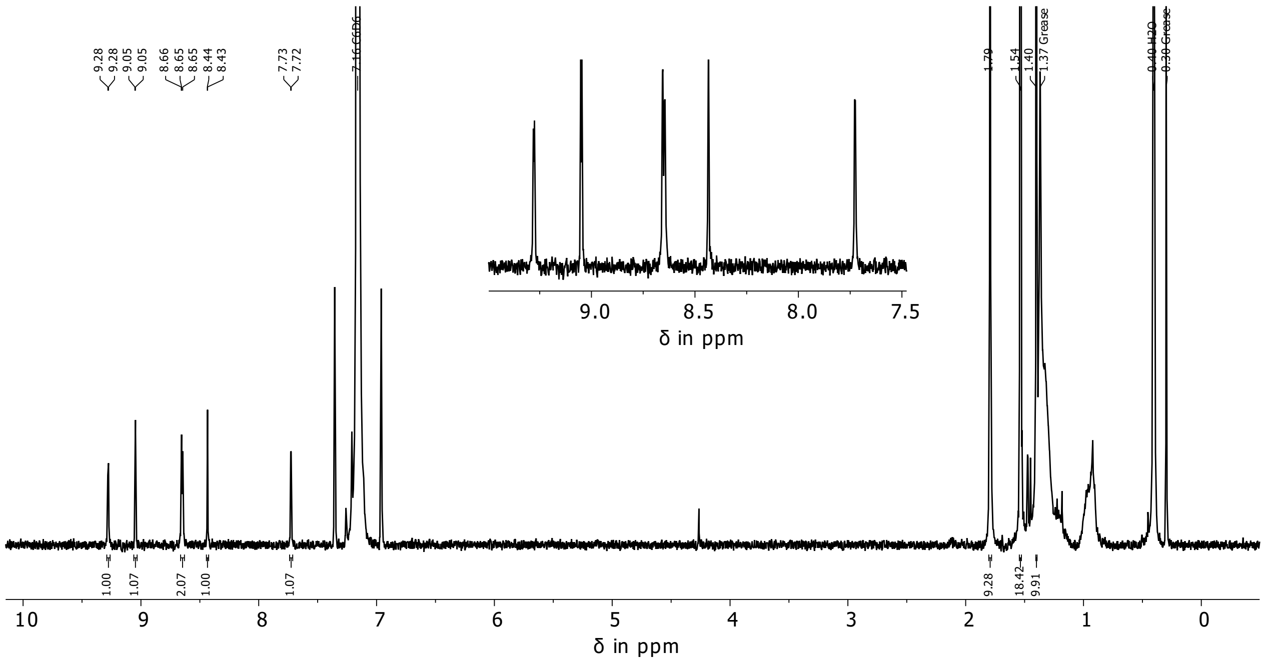

Figure S62 ^{1}H NMR spectrum of *Z*-**PBFT** (400 MHz, C_6D_6, 23 °C). Substantial amount of additional grease stems from the NMR solvent.

References

[S1] Huang, T.-H.; Lin, J. T.; Tao, Y.-T.; Chuen, C.-H. Benzo[*a*]Aceanthrylene Derivatives for Red-Emitting Electroluminescent Materials. *Chem. Mater.* **2003**, *15* (25), 4854–4862. https://doi.org/10.1021/cm034631e.

[S2] Toyota, S.; Ban, S.; Hara, M.; Kawamura, M.; Ikeda, H.; Tsurumaki, E. Synthesis and Properties of Rubicene-Based Aromatic π-Conjugated Compounds as Five-Membered Ring Embedded Planar Nanographenes. *Chem. Eur. J.* **2023**, *29* (49), e202301346. https://doi.org/10.1002/chem.202301346.

[S3] Itoh, T.; Mase, T. A General Palladium-Catalyzed Coupling of Aryl Bromides/Triflates and Thiols. *Org. Lett.* **2004**, *6* (24), 4587–4590. https://doi.org/10.1021/ol047996t.

[S4] Köttner, L.; Ciekalski, E.; Dube, H. *Peri* -Anthracenethioindigo: A Scaffold for Efficient All-Red-Light and Near-Infrared Molecular Photoswitching. *Angew. Chem. Int. Ed.* **2023**, *62* (52), e202312955. https://doi.org/10.1002/anie.202312955.

[S5] Henrieta Volfova, Qi Hu, Eberhard Riedle, *EPA Newsletter* **2019**, 51–69.
